# Local growth hormone promotes benign prostatic hyperplasia

**DOI:** 10.1172/jci.insight.206012

**Published:** 2026-07-28

**Authors:** Masaki Ryuzaki, Svetlana Zonis, Neil A. Bhowmick, Sandrine Billet, Saravana Kumar Kailasam Mani, Stephen J. Freedland, Hyung L. Kim, Vera Chesnokova, Shlomo Melmed

**Affiliations:** 1 Pituitary Center, Department of Medicine,; 2 Division of Medical Oncology, Department of Medicine, and; 3 Department of Urology, Cedars-Sinai Health Sciences University, Los Angeles, California, USA.

**Keywords:** Aging, Endocrinology, Mouse models

## Abstract

Locally produced nonpituitary growth hormone (npGH) promotes DNA damage accumulation and epithelial-mesenchymal transition (EMT) in aging human colon epithelium. GH receptor (GHR) and npGH are expressed in normal human prostate, and benign prostatic hyperplasia (BPH) prevalence increases with age. We hypothesized that local prostate GH action may promote EMT and contribute to BPH pathogenesis. We show here that the number of patients expressing npGH increases more than 10-fold after age 60, concordant with increased γH2AX, a marker of DNA damage, and EMT activation. GH-treated human primary prostate epithelial cells, normal prostate cells, and primary cell cultures derived from resected BPH specimens exhibited enhanced DNA damage and activated EMT, with induced TWIST2, suppressed E-cadherin, and increased Ki67, cell motility, and proliferation. In mice, prostate tissue adjacent to allografted GH-expressing fibroblasts showed increased γH2AX, TWIST2, and Ki67, along with morphological changes consistent with BPH. While GH and GH-induced IGF-1 both activated EMT, GH triggered DNA damage independently of IGF-1. These results elucidate what we believe to be a novel role for local npGH in aging prostate tissue, whereby npGH increases DNA damage and promotes EMT to enable a microenvironment favoring BPH development. Prostate GHR signaling may be an attractive therapeutic target for BPH.

## Abstract 2

Locally produced non-pituitary growth hormone (npGH) attenuates DNA repair and increases cell proliferation in the aging prostate, implicating npGH in age-associated benign prostatic hyperplasia development.

## Graphical abstract

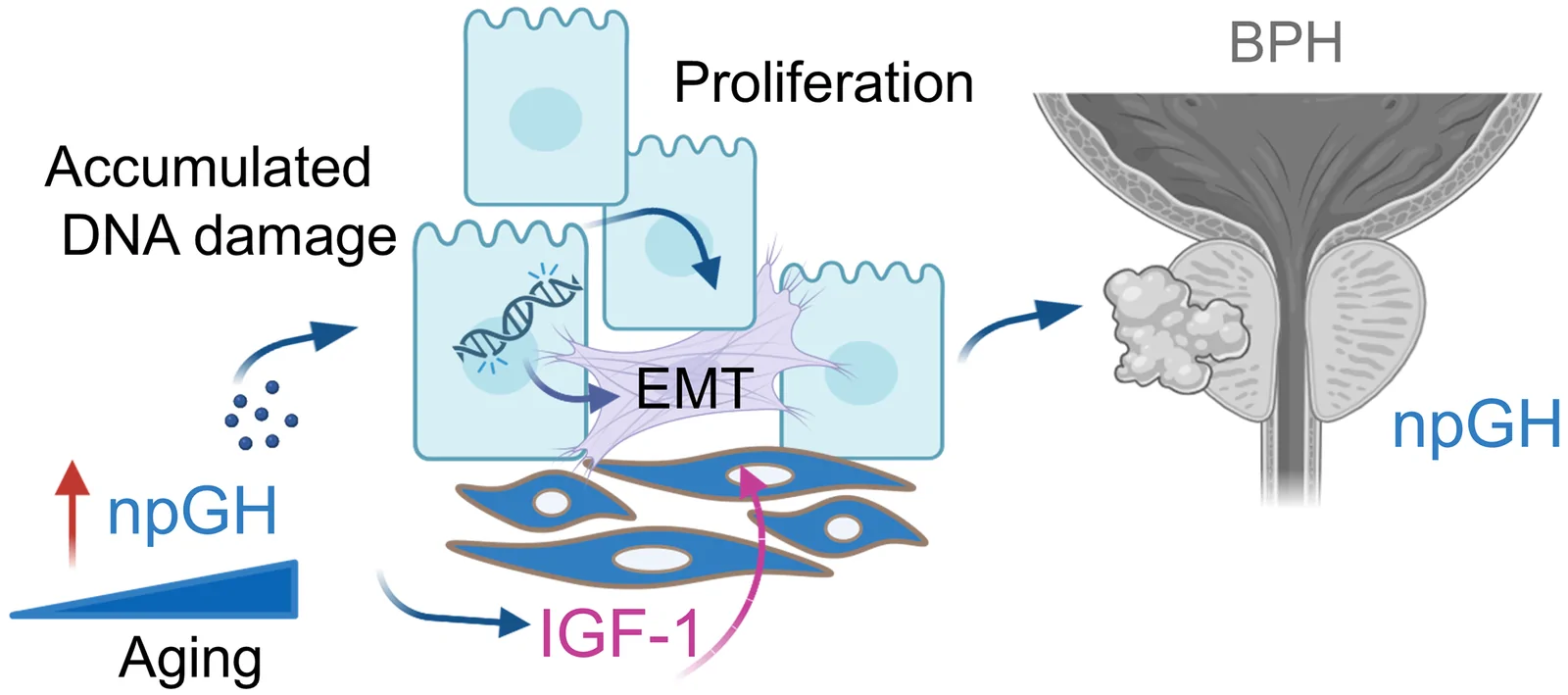

## Introduction

Growth hormone (GH) secreted by the anterior pituitary gland acts systemically by signaling through the ubiquitously expressed peripheral tissue GH receptor (GHR) (1). We have been studying locally produced non-pituitary GH (npGH) and showed its expression in human colon epithelium accumulates with DNA damage and aging (2). npGH elicits autocrine and paracrine actions to induce DNA damage accumulation by attenuating DNA repair via WT p53-inducible phosphatase 1 (Wip1), enabling a proneoplastic tissue microenvironment (3, 4). GHR and npGH are expressed in normal human prostate cells (5–7). While npGH is considered a protumorigenic factor in prostate tumors, its role in the benign tissue is not known. We therefore investigated npGH activity and paracrine GHR signaling in aging normal prostate tissue and in benign prostatic hyperplasia (BPH) marked by nodular epithelial and stromal fibroblastic proliferation in the prostate transitional zone (8). Although mechanisms underlying the pathogenesis of BPH are not fully understood (9–11), embryonic reawakening (12, 13), androgen/estrogen imbalance (9, 14), stromal-epithelial interactions (15), modulation of cell adhesion molecules (10), growth factors (16), increased TGF-β signaling (17), stem cell defects (18), and chronic inflammation (19, 20) have all been proposed.

BPH incidence increases with age, occurring in 20% of men more than 40 years of age, 70% of men over 60 years, and in 90% of men more than 80 years of age (8, 11, 21, 22). Although androgen levels decrease with age, the steroid persists as a major prostate growth regulator (23–25). Levels of circulating GH and its transcriptional target IGF-1 also decline markedly with age (26), yet in case-control studies, acromegaly patients with excess GH had prostate hypertrophy in men less than 40 years of age as well as those over 60 years (27, 28). Consistent with these observations, prostate volume is low in adults with GH deficiency (29) while GH replacement restores prostate size (30). Given these reports, and considering that both GH and IGF-1 are locally expressed in the prostate (7, 31, 32), we hypothesized that paracrine GH and IGF-1 actions may be involved in BPH pathogenesis.

Epithelial-mesenchymal transition (EMT) enables epithelial cells to acquire phenotypes with increased motility and invasiveness, resistance to apoptosis, and remodeled extracellular matrix (ECM) (33). EMT activation is associated with a loss of epithelial E-cadherin, required for cell-cell adhesion (34), and induction of mesenchymal markers, including transcription factors such as TWIST, SNAI1, SNAI2, ZEB1, and ZEB2 (35). Mechanisms triggering EMT are not fully understood, but DNA damage is a reported initiator (36–39). As we have shown that npGH induces TWIST2 and suppresses E-cadherin and the distorted ECM pathway in normal colon cells (40, 41), and as EMT is associated with BPH expansion (10, 42), we hypothesized that local GH/IGF-1 action in the prostate may promote EMT and contribute to BPH pathogenesis.

Our results show that age-associated DNA damage induces npGH in the aging human prostate, where it suppresses DNA repair pathways, thereby accelerating DNA damage accumulation. This, in turn, activates EMT in normal human prostate cells and in cell cultures derived from surgically resected BPH specimens. Furthermore, when GH-expressing fibroblasts were injected into the anterior prostate of C57BL/6 mice, tissue adjacent to the allografts exhibited elevated DNA damage, markers of EMT, and proliferation, as well as morphological changes consistent with BPH, all reflecting paracrine npGH activity. These results support our hypothesis that age-related prostate npGH induction may lead to BPH. We also showed that in the prostate GH signals to induce DNA damage independently of IGF-1 action, while effects of GH on EMT were partially IGF-1 dependent. The results suggested what we believe to be a novel role for local paracrine npGH signaling in regulating the prostate epithelial microenvironment and its involvement in pathogenesis of age-associated BPH.

## Results

### Prostate npGH expression increases with age concurrently with increased DNA damage.

**Figure 1:**
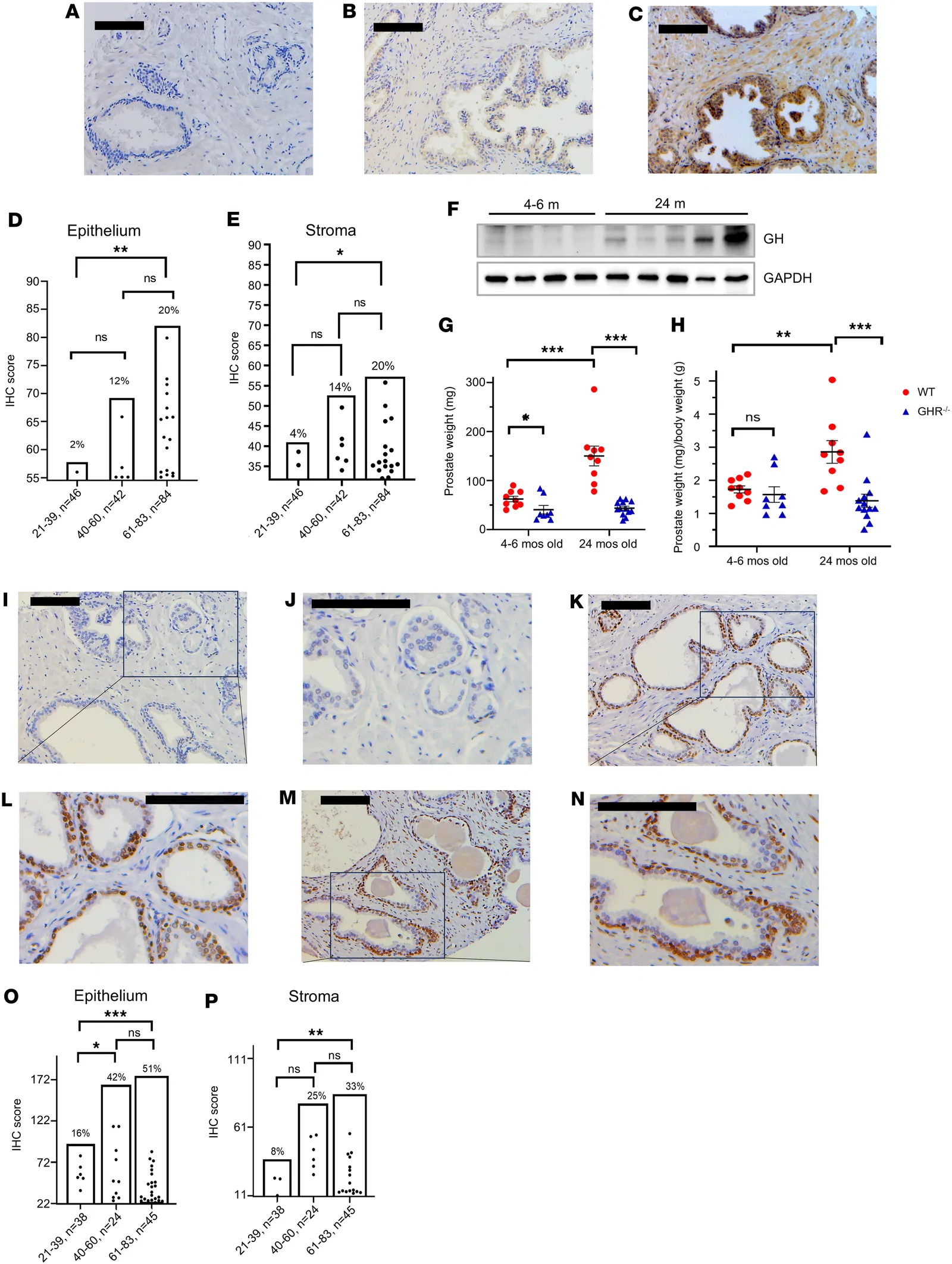
Prostate npGH and γH2AX expression increases with age. (**A**–**C**) Representative IHC images of GH expression (brown, cytoplasmic) in normal human prostate tissue specimens derived from individual patients aged (**A**) 35, (**B**) 47, or (**C**) 70 years. Scale bars: 100 μm. GH is expressed in both epithelial cells and in stroma in the aged group. (**D** and **E**) Graphs depict percentage of prostate tissue samples positive for GH in (**D**) epithelial cells and (**E**) stroma. IHC scores of ≥55 for epithelial cells and ≥32 for stroma were considered positive. Each dot represents an individual patient sample with positive GH expression. Results were analyzed by χ^2^ test, as shown in Supplemental Figure 1, A and B. (**F**) Western blot of GH expression in individual prostate tissues derived from C57BL/6 WT male mice aged 4–6 months and 24 months. ImageJ quantifications of Western blots are depicted in Supplemental Figure 1D. (**G**) Prostate weight and (**H**) prostate weight/body weight ratio in WT or *GHR^–/–^* mice aged 4–6 months and 24 months. (**I**–**N**) Representative IHC images of γH2AX expression (brown, intranuclear) in normal human prostate tissue specimens derived from patients aged (**I** and **J**) 28, (**K** and **L**) 40, or (**M** and **N**) 72 years. Magnification is ×10 in **I**, **K**, and **M**, and ×20 in insets in **J**, **L**, and **N**. Scale bars: 100 μm. (**O** and **P**) Graphs depict percentage of prostate tissue samples positive for γH2AX in (**O**) epithelial cells and (**P**) stroma. IHC scores of ≥22 for epithelial cells and ≥11 for stroma were considered positive. Each dot represents an individual patient sample with positive γH2AX. Results were analyzed by χ^2^ test, as shown in Supplemental Figure 1, E and F. *P < 0.05; **P < 0.01; ***P < 0.001.

Employing normal prostate tissue microarrays derived from young (21–39 years; *n* = 46), middle-aged (40–60 years; *n* = 42), and aged (61–83 years; *n* = 84) patients (Figure 1, A–C), we found that epithelial npGH expression increases with age. Cytoplasmic npGH was detected in 2% of samples derived from the young cohort, and in 12% and 20% of microarray samples derived from middle-aged and aged cohorts (*P* = 0.014435; Figure 1D and Supplemental Figure 1A; supplemental material available online with this article; https://doi.org/10.1172/jci.insight.206012DS1). Stromal npGH expression showed a similar age-dependent increase (*P* < 0.05; Figure 1, A–C, and E, and Supplemental Figure 1B) and correlated with epithelial npGH expression (*R*^2^ = 0.275, *P* < 0.0001; Supplemental Figure 1C). We found a similar age-associated increase in prostate npGH expression in WT C57BL/6 mice ages 4–6 months and 24 months (Figure 1F and Supplemental Figure 1D). These results confirmed that npGH expression appears to increase with aging in normal human and murine prostate tissue. While in WT mice prostate weight increased with age, in *GHR^–/–^* mice devoid of GHR signaling, prostate weight did not change with age, and the prostate weight/body weight ratio was significantly lower compared with aged WT mice (Figure 1, G and H).

As DNA damage induces npGH expression (2, 43), we examined expression of γH2AX, a DNA damage marker, in surgically resected human prostate tissue arrays (Figure 1, I–N, and Supplemental Figure 1, E and F). Epithelial γH2AX expression increased markedly with age (*P* = 0.003) and was detected in 16% of specimens in the young cohort (*n* = 38), and in 42% and 51% of normal prostate specimens derived from middle-aged and aged cohorts (*n* = 24 and *n* = 45, respectively; Figure 1O and Supplemental Figure 1E). Stromal γH2AX expression also increased with age (*P* = 0.02) (Figure 1P and Supplemental Figure 1F), and correlated with epithelial γH2AX expression (*R*^2^ = 0.3152, *P* < 0.001; Supplemental Figure 1G).

### Prostate cell DNA damage response triggers npGH, further enhancing DNA damage accumulation.

**Figure 2:**
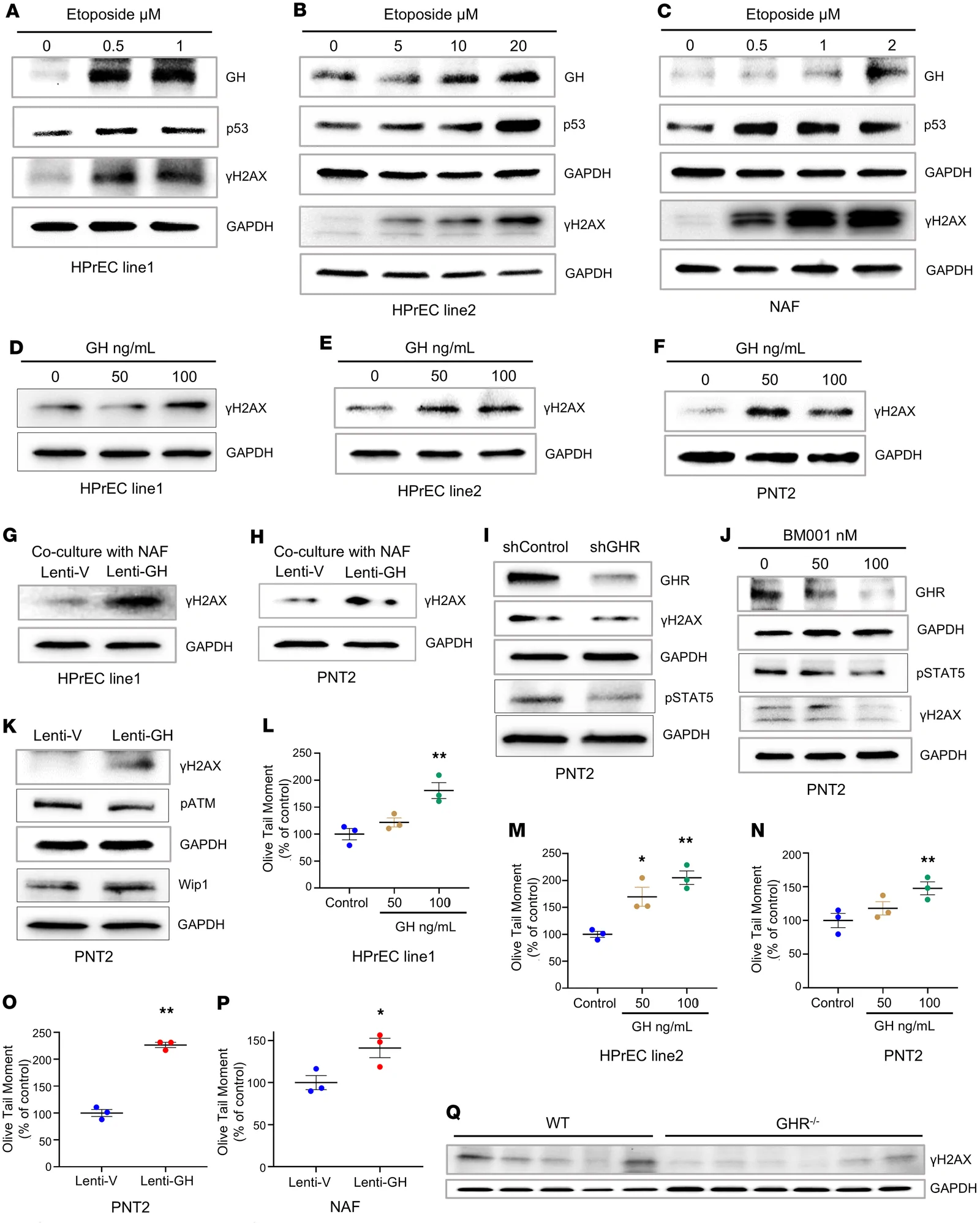
Prostate cell DNA damage response triggers npGH expression, further enhancing prostate DNA damage accumulation. (**A**–**C**) Western blots in (**A**) HPrEC line 1, (**B**) HPrEC line 2, and (**C**) NAFs treated with indicated doses of etoposide for 24 hours. (**D**–**F**) Western blots of (**D**) HPrEC line 1, (**E**) HPrEC line 2, and (**F**) PNT2 treated with indicated doses of GH and analyzed 24 hours later. (**G** and **H**) Western blots in (**G**) HPrEC line 1 and (**H**) PNT2 cocultured with lentiGH- or lentiV-infected NAFs for 24 hours. (**I**) Western blots in PNT2 infected with lentivirus expressing scramble shRNA (shControl) or GHR shRNA (shGHR) and analyzed 4 days later. (**J**) PNT2 treated with indicated doses of BM001 and analyzed 6 hours later. (**K**) Western blots in lentiGH- or lentiV-infected PNT2 and analyzed 4 weeks later. (**L**–**N**) Comet assay quantifying DNA damage in (**L**) HPrEC line 1, (**M**) HPrEC line 2, and (**N**) PNT2 treated with indicated doses of GH for 24 hours compared with control. (**O** and **P**) Comet assay quantifying DNA damage in (**O**) PNT2 and (**P**) lentiGH- or lentiV-infected NAFs and analyzed 4 weeks later. (**Q**) Western blot in prostate tissue derived from 24-month-old WT or *GHR^–/–^* mice. For **A**–**K**, representative blots from at least 3–4 independent experiments are shown. For **A**–**K**, ImageJ quantification of Western blots is depicted in Supplemental Figure 2, A–F, H–K, and M. For **L**–**Q**, 200–400 nuclei were analyzed in 3 separate experiments, each represented by 1 dot. Results shown are mean ± SEM. Results are shown as percentage of control, but statistical testing was performed on raw numbers. *P < 0.05, **P < 0.01 versus control by 2-way ANOVA followed by Tukey’s post hoc test to adjust for multiple comparisons (**L**–**N**) or 2-tailed Student’s *t* test (**O** and **P**). For **Q**, ImageJ quantifications of Western blots are depicted in Supplemental Figure 2P.

As both GH and DNA damage appeared to accumulate concurrently in aging human prostate cells, we considered whether activated DNA damage response triggers local prostate npGH, as was previously shown in colon cells (2, 44). We induced DNA damage in non-tumorous primary human prostate epithelial cells (HPrECs) derived from 2 separate young individuals, as well as in normal human prostate–associated fibroblasts (NAFs). Treatment with etoposide, a topoisomerase II inhibitor producing both single-strand and double-strand DNA breaks, induced DNA damage, as evidenced by increased levels of p53 and γH2AX, and also enhanced npGH expression in all prostate cell models tested (Figure 2, A–C, and Supplemental Figure 2, A–C), indicating that local prostate npGH expression is activated by DNA damage.

To elucidate endocrine GH action in prostate epithelial cells, we treated HPrEC line 1, HPrEC line 2, and the human prostate epithelial cell line PNT2 with GH (50 and 100 ng/mL) and demonstrated markedly increased γH2AX expression in all lines after 24 hours (Figure 2, D–F, and Supplemental Figure 2, D–F). To explore a paracrine mechanism for npGH action on prostate epithelial cells, human NAFs were infected with lentivirus expressing either hGH (lentiGH) or vector (lentiV) (Supplemental Figure 2G), then cocultured with either HPrEC line 1 or PNT2 for 24 hours. HPrEC line 1 or PNT2 exposed to paracrine GH expressed increased γH2AX levels compared with lentiV NAF cocultures (Figure 2, G and H and Supplemental Figure 2, H and I). We confirmed the specificity of paracrine npGH action by infecting PNT2 with lentivirus stably expressing short hairpin GHR (shGHR) and by blocking GHR synthesis with the small molecule inhibitor BM001. As GH action is mediated by STAT5, abrogation of GH signaling was also confirmed by decreased expression of p-STAT5, and attenuated γH2AX expression in both experimental models (Figure 2, I and J, and Supplemental Figure 2, J and K).

To investigate the mechanisms of npGH effects on DNA damage, PNT2 cells were infected with lentiGH or lentiV (Supplemental Figure 2L). Our earlier experiments show that GH induces DNA damage by activating Wip1, which, in turn, suppresses phosphorylation of ATM and p53, diminishing DNA repair pathway activity. Indeed, PNT2 cells infected with lentiGH exhibit increased Wip1 and decreased pATM, consistent with increased γH2AX expression (Figure 2K and Supplemental Figure 2M). GH also activated Wip1 and suppressed both pATM and p53 in HPrEC cells 3–6 hours after the treatment, preceding the effects on γH2AX (Supplemental Figure 2, N and O). DNA damage quantifications assessed by comet assay were consistent with elevated γH2AX expression observed by Western blot. Specifically, treatment with 100 ng/mL GH for 24 hours increased DNA damage approximately 80% in HPrEC line 1, approximately 100% in HPrEC line 2, and approximately 50% in PNT2 (Figure 2, L–N). PNT2 and NAFs infected with lentiGH similarly exhibited DNA damage accumulation (~130% and ~40%, respectively) compared with control (Figure 2, O and P).

In agreement with in vitro results, prostate tissue harvested from 24-month-old *GHR^–/–^* mice showed lower γH2AX expression, indicative of decreased DNA damage accumulation, compared with WT (Figure 2Q and Supplemental Figure 2P).

Overall, these results showed both endocrine and autocrine/paracrine GH act to enhance prostate epithelial cell DNA damage accumulation.

### Age-associated stimulation of prostate EMT and proliferation is mediated by npGH.

**Figure 3:**
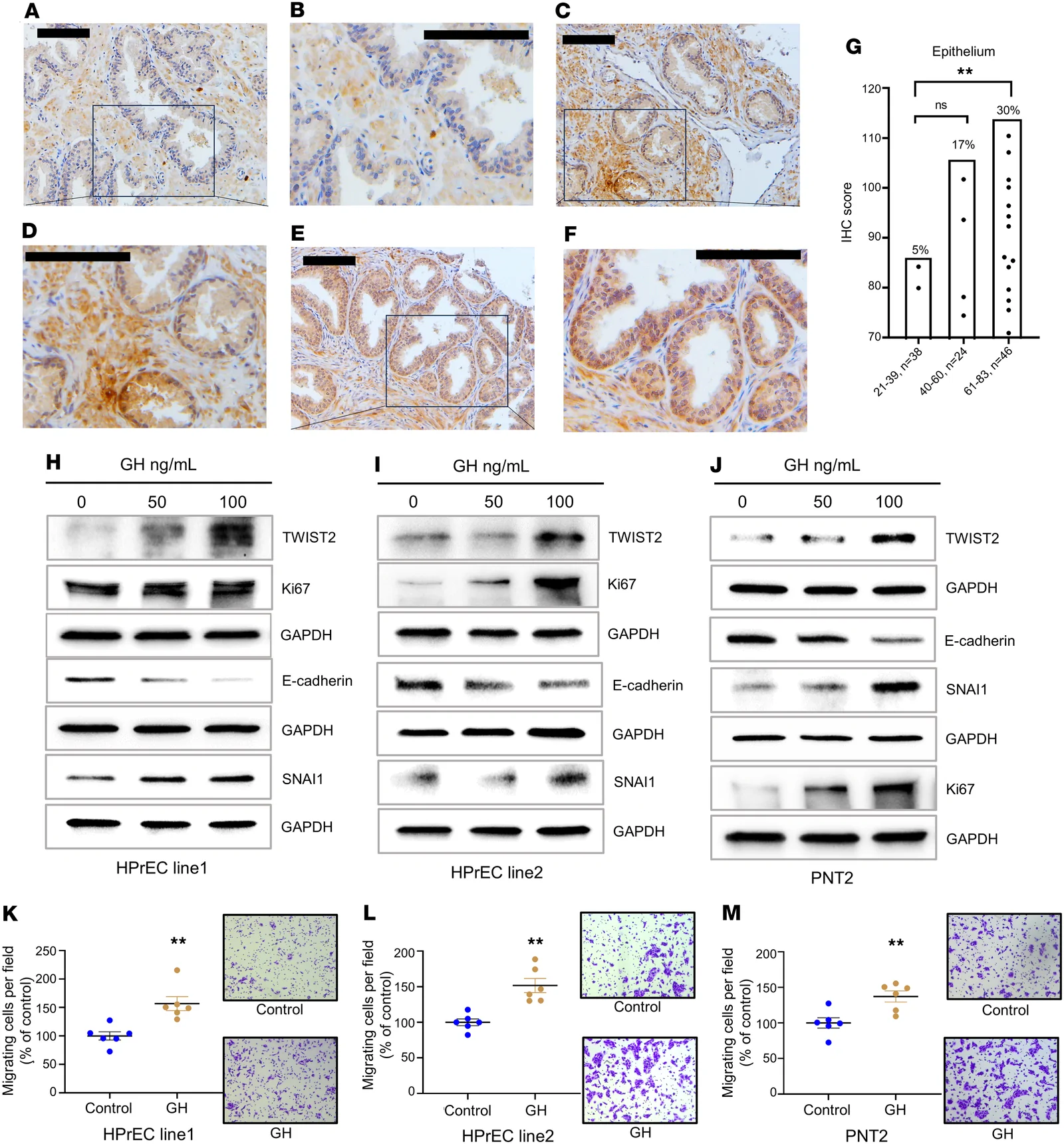
Age-associated stimulation of prostate EMT and proliferation are mediated by npGH. (**A**–**F**) Human prostate TWIST1/2 expression increases with age. Representative IHC images of TWIST1/2 expression (brown) in normal human prostate tissue specimens derived from patients aged (**A** and **B**) 30, (**C** and **D**) 45, and (**E** and **F**) 80 years. Magnification is ×10 in **A**, **C**, and **E**, and ×20 in insets in **B**, **D**, and **F**. Scale bars: 100 μm. (**G**) Graph depicts percentage of samples positive for TWIST1/2 in normal human prostate tissue. IHC scores of ≥70 were considered positive. Each dot represents an individual patient with positive TWIST1/2. **P < 0.01 by χ^2^ test, as shown in Supplemental Figure 3A. (**H**–**J**) Endocrine GH induces prostate cell EMT and proliferation. Western blot of EMT markers in (**H**) HPrEC line 1, (**I**) HPrEC line 2, and (**J**) PNT2. Cells were treated with indicated GH doses and analyzed 24 hours later. Representative blots from at least 2–4 independent experiments are shown. ImageJ quantification of Western blots is depicted in Supplemental Figure 3, C–E. For **H**, the Western blot membrane from Figure 2D was stripped and re-probed for TWIST2 and Ki67; the GAPDH loading control is the same. (**K**–**M**) Migration of (**K**) HPrEC line 1, (**L**) HPrEC line 2, and (**M**) PNT2 cells treated with GH. Cells were plated into migration chambers with GH (100 ng/mL) or left untreated (control); 24 hours later, cells were stained with crystal violet, imaged (magnification, ×100), and the number of migrated cells was assessed. Results shown are mean ± SEM. Each dot represents results of 3 separate experiments performed in duplicate. Results are shown as percentage of control, but statistical testing was performed on raw numbers. Results in **K**–**M** were analyzed by 2-tailed Student’s *t* test. **P < 0.01 versus control.

We next examined local npGH action on prostate cell EMT. In human prostate tissue samples obtained at surgical resection, immunohistochemical analysis showed that epithelial transcription factors TWIST1 and -2, important for triggering EMT, were induced with age (*P* = 0.012; Figure 3, A–F, and Supplemental Figure 3A). TWIST1 and -2 were detected in 5% of samples from the young cohort (*n* = 38), in 17% from the medium-aged (*n* = 24), and 30% of the aged cohorts (*n* = 46; Figure 3G and Supplemental Figure 3A). TWIST1/2 expression strongly correlated with local npGH expression in normal prostate epithelial cells (*R*^2^ = 0.248, *P* < 0.0001; Supplemental Figure 3B). In sum, these results suggest that age-associated increased prostate epithelial npGH expression occurs concurrently with EMT activation.

We next tested whether endocrine GH alters EMT in normal non-tumorous prostate epithelial cells. Treatment with GH induced TWIST2 and SNAI1 expression in both HPrEC cell lines and in PNT2 cells. GH also dose-dependently increased *TWIST1*, *TWIST2*, and *SNAI1* mRNA levels in PNT2 cells. By contrast, E-cadherin, required for cell-to-cell adhesion, was decreased by GH treatment, consistent with activated EMT, while Ki67, a marker of cell proliferation, was increased (Figure 3, H–J, and Supplemental Figure 3, C–F). Consistent with these results, we observed enhanced cell migration in both HPrEC lines and PNT2 (60%, 50%, and 30%, respectively) 24 hours after GH treatment (Figure 3, K–M). These changes in EMT markers and enhanced migration likely reflect mesenchymal differentiation in response to GH treatment of non-malignant prostate epithelia.

### Autocrine/paracrine GH induces prostate EMT and proliferation.

**Figure 4:**
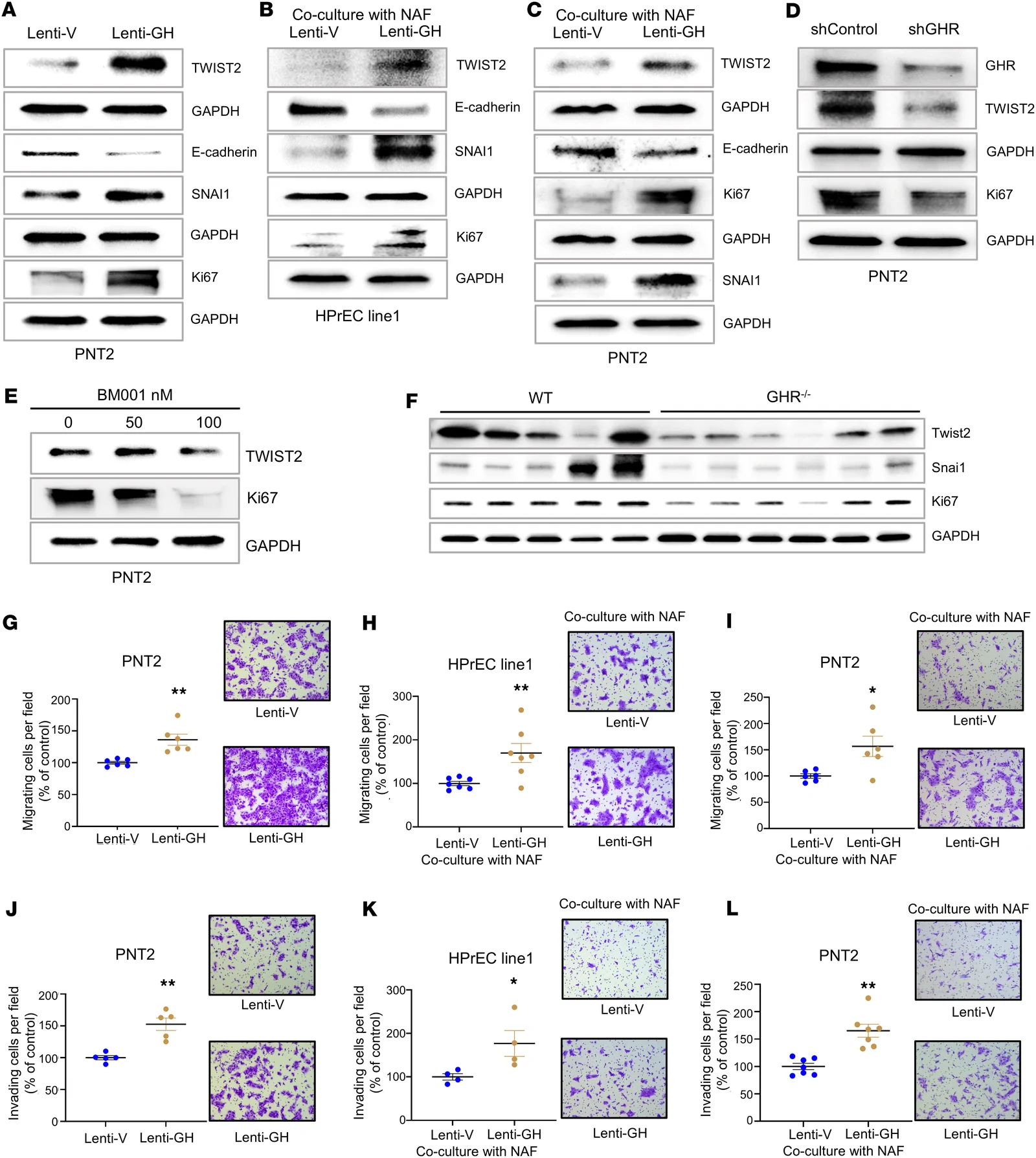
Autocrine/paracrine GH induces prostate EMT and proliferation. (**A**–**E**) Western blots of EMT marker expression in (**A**) PNT2 infected with lentiGH or lentiV and analyzed 4 weeks later, (**B**) HPrEC line 1 or (**C**) PNT2 cocultured for 24 hours with NAFs infected with lentiGH or lentiV; (**D**) PNT2 infected with lentivirus expressing control shRNA (shControl) or GHR shRNA (shGHR) and collected after 4 days; and (**E**) PNT2 treated with indicated doses of BM001 and analyzed 6 hours later. Representative blots from at least 3 independent experiments are shown. (**F**) Western blot of EMT marker expression in prostate tissue samples derived from 24-month-old WT (*n* = 5) and *GHR^–/–^* (*n* = 6) mice. For **A**–**F**, ImageJ quantification of Western blots is depicted in Supplemental Figure 4, A–E, and G. For **B**, the Western blot membrane from Figure 2G was stripped and re-probed for Ki67; the GAPDH loading control is the same. (**G**-**I**) Migration and (**J**-**L**) invasion of (**G** and **J**) PNT2 cells infected with lentiGH and lentiV and (**H** and **K**) HPrEC or (**I** and **L**) PNT2 cells co-cultured with lentiGH- or lentiV-infected NAF. Migrated or invaded cells were stained with crystal violet, imaged (magnification, ×100), and cells counted. Results shown are mean ± SEM/field. Each dot represents results of 2–3 separate experiments in duplicate or triplicate. Results are shown as percentage of control, but statistical testing was performed on raw numbers. Results were analyzed by 2-tailed Student’s *t* test. *P < 0.05, **P < 0.01 versus control.

To test whether autocrine/paracrine npGH exerts similar effects on EMT, PNT2 were infected with lentiGH or lentiV, and HPrEC line 1 and PNT2 cells were cocultured with lentiGH- or lentiV-infected NAFs. Induction of TWIST2 and SNAI1, and suppression of E-cadherin were observed in cells overexpressing GH (Figure 4A and Supplemental Figure 4A) as well as in prostate cells exposed to paracrine npGH (Figure 4, B and C, and Supplemental Figure 4, B and C). To confirm the specificity of paracrine npGH signaling, we treated PNT2 with lentivirus expressing shGHR or with the GHR synthesis blocker BM001. Both inhibitors of GH signaling blocked the induction of TWIST2 (Figure 4, D and E, and Supplemental Figure 4, D and E) and BM001 treatment suppressed TWIST2 mRNA expression concurrently with suppressed GHR mRNA expression (Supplemental Figure 4F). Similarly, 2-year-old *GHR^–/–^* mice devoid of GH signaling showed decreased prostate Twist2 and Snai1 (Figure 4F and Supplemental Figure 4G). Furthermore, in both HPrEC line 1 and PNT2 as well as in *GHR^–/–^* mice, while GH induced Ki67, the effect was blunted by blocking GHR signaling (Figure 4, A–F, and Supplemental Figure 4, A–G). We also observed enhanced migration and invasion of PNT2 cells infected with lentiGH versus lentiV (Figure 4, G and H), as well as in HPrEC line 1 (Figure 4, I and J) and PNT2 cells (Figure 4, K and L) cocultured with NAFs infected with lentiGH versus lentiV. Overall, the results indicate that autocrine/paracrine npGH induces EMT in prostate epithelium.

### IGF-1 induces EMT and proliferation, but attenuates DNA damage in human prostate cells.

**Figure 5:**
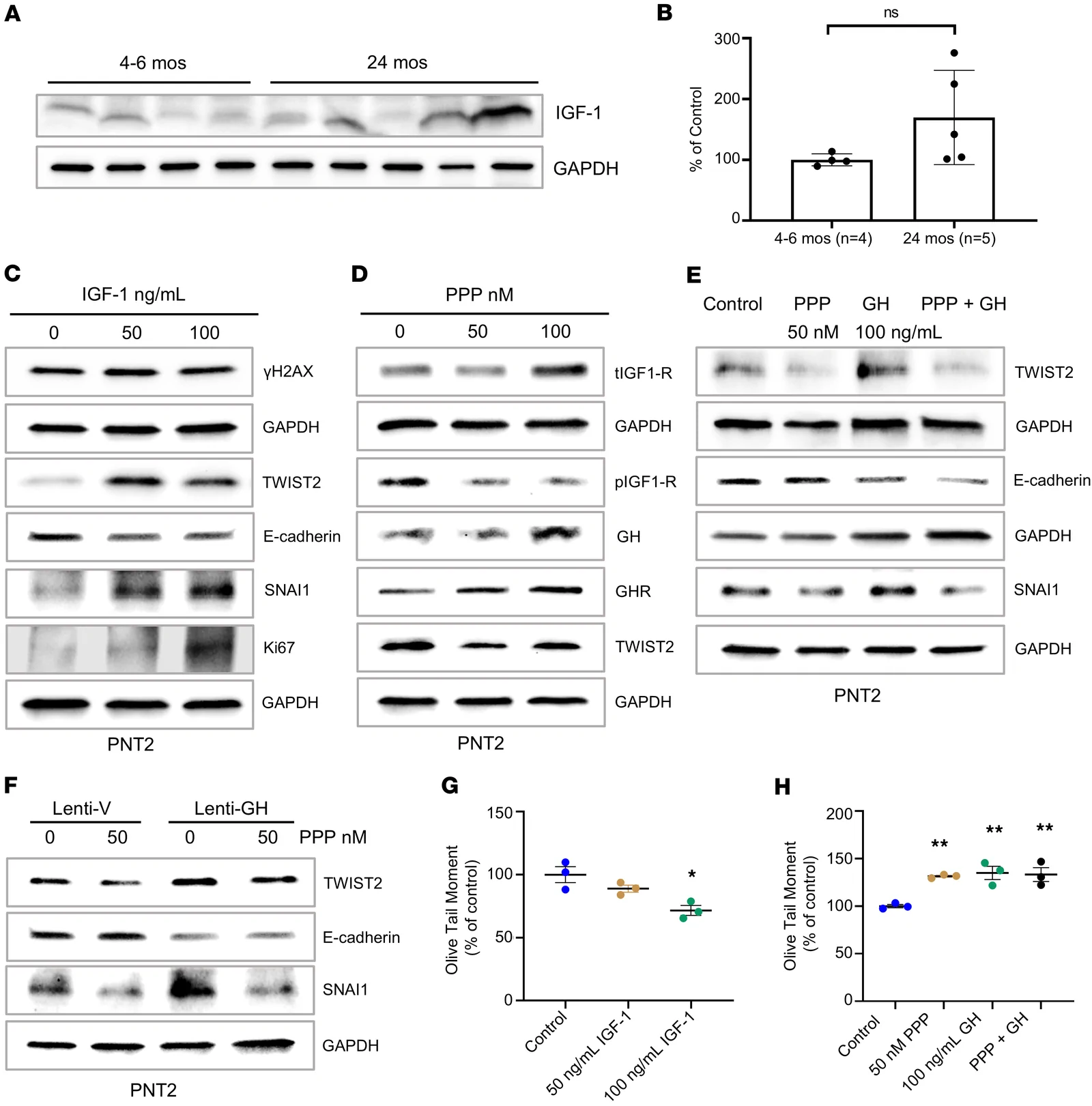
IGF-1 induces EMT and proliferation, but attenuates DNA damage. (**A** and **B**) IGF-1 expression in prostate tissue derived from 4- to 6-month-old and 24-month-old WT male mice. (**A**) Western blot. (**B**) ImageJ quantification of expression normalized to loading controls. Average measurements of four 4- to 6-month-old and five 24-month-old mice are shown. Each dot represents sample analysis derived from an individual animal. Results are shown as mean ± SEM for each group. Data are graphed as percentage of control, but statistical testing was performed on raw numbers. Differences were assessed with 2-tailed Student’s *t*-test. (**C** and **D**) Western blots of protein expression in PNT2 cells treated with indicated doses of (**C**) IGF-1 or (**D**) IGF-1R inhibitor PPP and analyzed 24 hours later. tIGF-1R, total IGF-1 receptor; pIGF-1R, phosphorylated IGF-1R. (**E**) Western blot of protein expression in PNT2 cells treated with 50 nM PPP, 100 ng/mL GH, or both. (**F**) Western blot of protein expression in PNT2 cells infected with lentiGH or lentiV, treated with 50 nM PPP after 4 weeks and analyzed 24 hours later. (**G** and **H**) Comet assay in PNT2 cells treated with indicated doses of (**G**) IGF-1 or (**H**) PPP, GH, or both for 24 hours. Results shown are mean ± SEM. Each dot in **G** and **H** represents a single experiment in which 300–400 nuclei were analyzed. Results are shown as percentage of control, but statistical testing was performed on raw numbers. Results were analyzed by 2-way ANOVA followed by Tukey’s multiple-comparison test. *P < 0.05, **P < 0.01 versus control. For **C**–**F**, representative blots of at least 3 experiments are shown. ImageJ quantification of protein expression is shown in Supplemental Figure 5, S–V.

To further understand mechanisms of GH action in prostate epithelium, we next assessed whether the effects were mediated through IGF-1 or directly through GH/GHR signaling. IGF-1 levels were not significantly increased with age in WT mice (Figure 5, A and B), whereas IGF-1 expression was induced in cells treated with etoposide or GH, in PNT2 cells overexpressing GH, and in HPrEC line 1 and PNT2 cells cocultured with GH-expressing NAFs (Supplemental Figure 5, A–R). To examine direct IGF-1 involvement in GH action, we treated PNT2 cells with IGF-1 or picropodophyllin (PPP), an inhibitor of IGF-1 receptor (IGF-1R) tyrosine autophosphorylation (45). IGF-1 markedly increased TWIST2 and SNAI1 expression and decreased E-cadherin expression. Although IGF-1 elevated Ki67, there was no impact on γH2AX expression (Figure 5C and Supplemental Figure 5S). As expected, PPP treatment of PNT2 cells suppressed IGF-1R phosphorylation, but expression of IGF-1R, GH, and GHR was induced, suggestive of a negative feedback autoregulatory loop. Suppression of IGF-1 signaling with PPP also markedly suppressed TWIST2 expression (Figure 5D and Supplemental Figure 5T). We next tested the effect of PPP on GH action in PNT2 cells. PPP treatment suppressed TWIST2 and SNAI1 and did not alter E-cadherin expression, whereas the addition of PPP to GH treatment suppressed GH-induced increases in TWIST2 and SNAI1, but did not reverse GH-induced suppression of E-cadherin (Figure 5E and Supplemental Figure 5U). Similar results were observed with PPP treatment of PNT2 cells infected with lentiV or lentiGH, as PPP suppressed TWIST2 and SNAI1 but had no effect on E-cadherin in lentiV-infected PNT2 (Figure 5F and Supplemental Figure 5V). In contrast to the demonstrated increase in DNA damage with GH treatment (Figure 2), comet assay in PNT2 cells showed a dose-dependent decrease in DNA damage over 24 hours of IGF-1 treatment (Figure 5G), consistent with the observation that IGF-1R signaling promotes DNA damage repair (46). As GH can also act through IGF-1R (47), we tested whether blocking this receptor affects GH-induced DNA damage accumulation. We found an approximately 30% increase in DNA damage in PNT2 cells treated with PPP, with GH, or with both PPP and GH (Figure 5H). Together, these results suggest that GH-induced local IGF-1 may at least partially activate EMT and increase proliferation. However, the DNA damage increase is mediated directly through GH/GHR signaling independently of IGF-1.

### GH suppresses DNA damage repair pathways in HPrECs.

**Figure 6:**
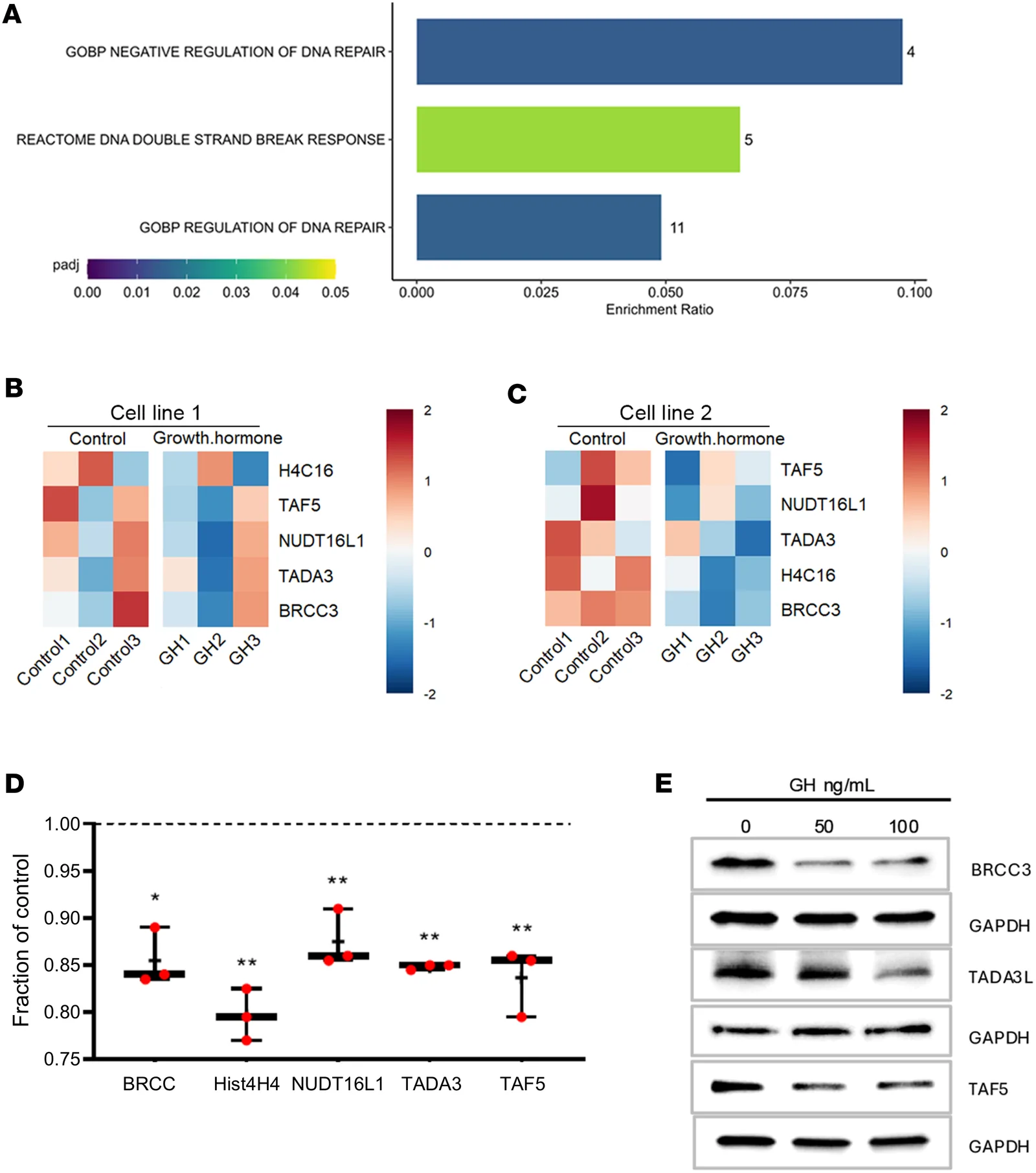
GH suppresses DNA damage repair pathways in HPrECs. RNA-seq conducted on RNA extracted from HPrEC line 1 or HPrEC line 2 with or without treatment of 100 ng/mL GH for 24 hours. Results from 3 independent experiments shown. (**A**) Bar plot showing DNA repair related pathways identified by over-representation analysis. The *x* axis (enrichment ratio) represents the ratio of differentially expressed genes (DEGs) within a specific pathway to the total number of genes annotated to that pathway. The number of DEGs within each pathway is given next to its respective bar. *P* value is indicated by color of the bar. (**B** and **C**) Heatmaps of 5 representative genes associated with the DNA damage repair pathway showing significant downregulation in response to GH treatment in (**B**) HPrEC line 1 or (**C**) HPrEC line 2. (**D**) Real-time PCR of mRNA expression of genes identified as significantly suppressed by RNA-seq in HPrEC line 2 treated with 100 ng/mL GH for 24 hours. Normalized PCR results are expressed as fraction of control taken as 1. The experiment was performed in triplicate. Differences were assessed with 2-tailed Student’s *t* test. *P < 0.05, **P < 0.01 versus control. (**E**) Western blot of DNA repair protein expression in HPrEC line 2 treated with indicated doses of GH for 24 hours. Representative blots from at least 3 independent experiments are shown. ImageJ quantification is depicted in Supplemental Figure 6.

Next, employing RNA-seq and subsequent bioinformatics analysis, we found that several signaling pathways were altered and genes differentially expressed in both HPrEC line 1 and line 2 treated with 100 mg/mL GH for 24 hours (Supplemental Figure 6, A and B). We then sought to identify regulators of DNA damage repair in HPrECs exposed to GH (Figure 6, A–C). Specifically, BRCA1/BRCA2-containing complex subunit 3 (BRCC3) (*P* = 0.029 in line 1 and *P* = 0.043 in line 2), TAF5 (*P* = 0.001 in line 2), H4C16 (*P* = 0.018 in line 2), NUDT16L1 (*P* = 0.016 in line 2), and TADA3 (*P* = 0.021 in line 2), all pivotal for DNA damage repair (48–54), were downregulated by GH treatment. Real-time PCR confirmed RNA-seq results (Figure 6D). Furthermore, Western blot analysis showed suppressed BRCC3, TADA3L, and TAF5 protein expression with GH treatment (Figure 6E and Supplemental Figure 6C). These findings define a potential mechanism underlying GH-induced prostate cell DNA damage accumulation.

### npGH, γH2AX, and TWIST1/2 expression is increased in both aged normal human prostate tissue and in BPH.

**Figure 7:**
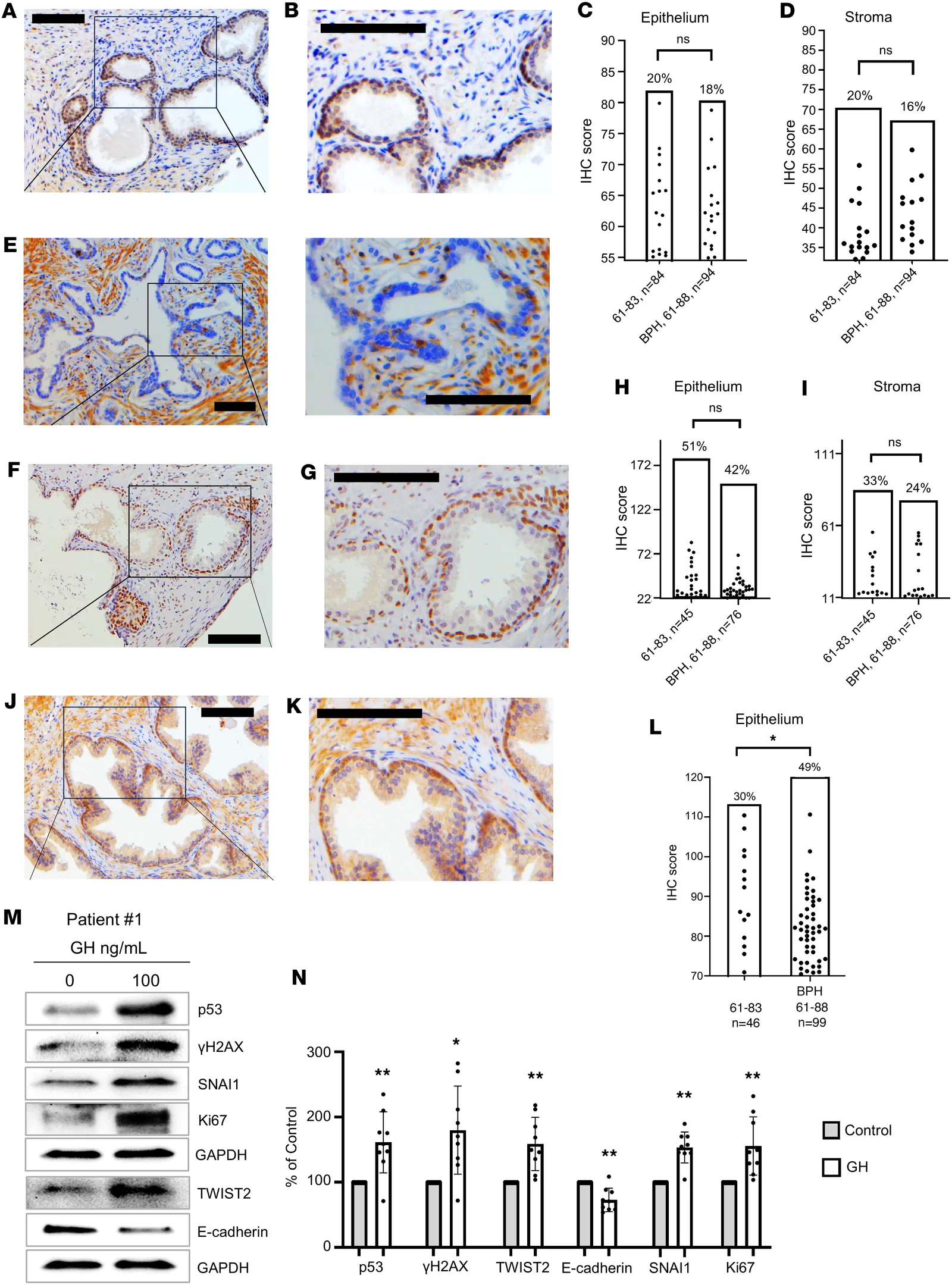
npGH, γH2AX, and TWIST1/2 expression is increased in aged normal human prostate tissue and in BPH. (**A** and **B**) Representative IHC images of GH expression (brown) in BPH specimens from a 68-year-old patient. (**C** and **D**) Percentage of GH-positive normal prostate or BPH (**C**) epithelium and (**D**) stroma. Scores of ≥55 for epithelium and ≥32 for stroma were considered positive. Each dot represents 1 patient. Results were analyzed by χ^2^ test, as shown in Supplemental Figure 7, A and B. (**E**) Representative IHC images of intranuclear p-STAT expression (brown) in BPH specimen. (**F** and **G**) Representative IHC images of γH2AX expression (brown) in BPH specimen from a 64-year-old patient. (**H** and **I**) Percentage of γH2AX-positive normal prostate or BPH (**H**) epithelium and (**I**) stroma. Scores of ≥22 for epithelium and ≥11 for stroma were considered positive. Each dot represents 1 patient. Results were analyzed by χ^2^ test, as shown in Supplemental Figure 7, D and E. (**J** and **K**) Representative IHC images of TWIST1/2 expression (brown) in BPH specimen from a 70-year-old patient. Magnification, ×10 (**A**, **F**, and **J**) and ×20 (**B**, **G**, and **K** insets). Scale bars: 100 μm. (**L**) Percentage of TWIST1/2-positive normal prostate or BPH samples. Scores of ≥70 were considered positive. Each dot represents 1 patient. Results were analyzed by χ^2^ test, as shown in Supplemental Figure 7H. (**M** and **N**) DNA damage, EMT, and proliferation protein expression in primary human BPH cultures treated overnight with 100 ng/mL GH. (**M**) Western blot in cells from a single patient. (**N**) ImageJ quantification of protein expression normalized to loading controls in cells from 9 patients. Each dot represents 1 patient. Results are shown as mean ± SEM. Results are shown as percentage of control, but statistical testing was performed on raw numbers. Results were analyzed by 2-tailed Student’s *t* test. *P < 0.05, **P < 0.01 versus control. Patient characteristics are shown in Table 1.

We showed that both epithelial and stromal npGH, γH2AX, and TWIST2 all markedly increase with age (Figure 1, A–C, and I–N, and Figure 3, A–F). As both EMT and DNA damage accumulation contribute to pathogenesis of age-related BPH (10, 42), we examined whether local npGH induction of DNA damage and EMT enables BPH development. Comparing commercially available BPH tissue microarrays with normal prostate tissue, we found epithelial BPH npGH expression was similar to npGH expression in the aged normal prostate (Figure 7, A–C, and Supplemental Figure 7A). Stromal npGH expression in BPH also was not different from the aged normal prostate cohort (Figure 7, A, B, and D, and Supplemental Figure 7B). npGH abundance in BPH epithelium correlated with abundance in stromal fibroblasts (*R*^2^ = 0.2751, *P* < 0.0001; Supplemental Figure 7C). To confirm that GH in BPH is active, we immunostained BPH samples with anti–p-STAT5 antibodies (Figure 7E) and found that in BPH epithelial GH positively correlates with p-STAT5 expression (*R*^2^ = 0.1186, *P* = 0.0076, Supplemental Figure 7K). Assessment by immunohistochemistry (IHC) revealed similar frequency of epithelial and stromal γH2AX expression in BPH samples and aged normal prostate (Figure 7, F–I, and Supplemental Figure 7, D and E). BPH epithelial γH2AX correlated with stromal γH2AX expression (*R*^2^ = 0.3954, *P* < 0.0001; Supplemental Figure 7F). However, BPH epithelial TWIST1/2 expression markedly increased compared with aged normal cohorts (*P* < 0.05; Figure 7, G–L, and Supplemental Figure 7H). Importantly, γH2AX in BPH epithelium also correlated with epithelial npGH expression (*R*^2^ = 0.1305, *P* = 0.0049; Supplemental Figure 7G) and epithelial TWIST1/2 expression correlated with epithelial GH (*R*^2^ = 0.0875, *P* = 0.007) and with γH2AX (*R*^2^ = 0.06291, *P* = 0.0422; Supplemental Figure 7, I and J). Taken together, these results indicated that npGH, DNA damage, and EMT are similarly induced in BPH and in the normal aged cohort and that in BPH both γH2AX and TWIST2 are connected to npGH.

### GH induces DNA damage, EMT, and proliferation in cultured human BPH cells.

We next generated primary cultures derived from 9 freshly resected human BPH surgical specimens (Table 1). We first confirmed that GH is induced in BPH tissue compared with HPrECs derived from young healthy individuals. Indeed, GH mRNA levels were markedly higher in all BPH specimens (Supplemental Figure 8A). BPH cultures were treated overnight with 100 ng/mL GH (Figure 7, M and N). p53 and γH2AX were induced by GH in all but one patient, suggesting GH induced DNA damage. Furthermore, expression of TWIST2 and SNAI1 was enhanced, whereas E-cadherin expression decreased with GH treatment, indicative of EMT activation. GH also induced Ki67 expression, a marker of proliferation. Thus, these results support our hypothesis that GH may act to facilitate BPH growth.

### Endocrine/paracrine GH induces changes consistent with BPH.

**Figure 8:**
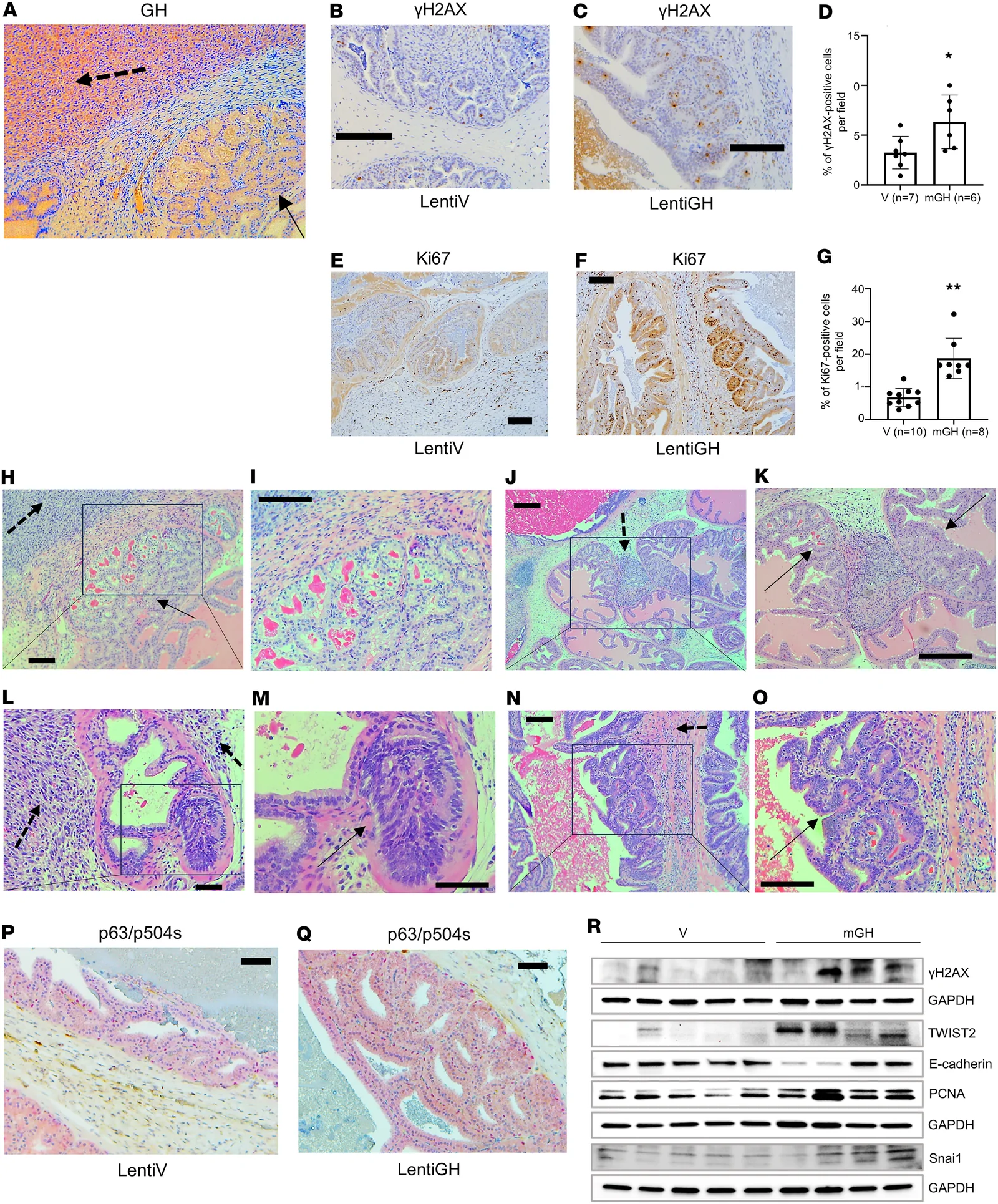
Endocrine/paracrine GH induces changes consistent with BPH. Murine anterior prostate glands in 8-week-old C57BL/6 mice were injected with 6 × 10^5^ mouse prostate fibroblasts infected with lentivirus expressing mouse GH (mGH) or empty vector (V) and euthanized after 2 months. (**A**) Representative IHC image of GH expression (brown) in allograft tumor (dashed arrow) and epithelial hyperplasia focus (solid arrow) in close proximity to GH-secreting allograft tumor. (**B** and **C**) Representative IHC images of γH2AX expression (brown) in the prostate in close proximity to allograft tumors expressing (**B**) lentiV or (**C**) lenti-mGH. (**D**) Percentage of cells positive for γH2AX detected by IHC. (**E** and **F**) Representative IHC images of Ki67 expression (brown) in the prostate in close proximity to allograft tumors expressing (**E**) lentiV or (**F**) lenti-mGH. (**G**) Percentage of cells positive for Ki67 detected by IHC. γH2AX staining in **D** and Ki67 staining in **G** are presented as average percentage of positively stained anterior prostate epithelial cells from 5–7 images per individual mouse. Results are presented as mean ± SEM. Differences were assessed with 2-tailed Student’s *t* test. *P < 0.05, **P < 0.01 versus control. (**H**–**O**) Representative H&E images of prostate tissue from mice injected with lenti-mGH fibroblasts. Magnification, ×10 (**H**, **J**, **L**, and **N**) and ×20 (**I**, **K**, **M**, and **O** insets). Hyperplastic (BPH) areas are marked by solid arrows and allograft tumor areas are marked by dashed arrows. Scale bars: 50 μm. (**P** and **Q**) Representative double-staining image of p63 (red) and p504s (brown) in a prostate specimen injected with fibroblasts infected with (**P**) lentiV or (**Q**) lenti-mGH imaged at ×10. Scale bars: 50 μm. BPH is positive for p63 but negative for p504s. Stromal brown cells depicting lymphocytes positive for p504s served as a positive control. (**R**) Western blot of dorsolateral prostate γH2AX, EMT markers, and PCNA. ImageJ quantification is shown in Supplemental Figure 9G.

To assess the role of age-associated npGH induction in promotion of BPH development, both anterior prostate glands of 8-week-old syngeneic C57BL/6 mice were allografted with 6 × 10^5^ lentivirus-infected mouse fibroblasts expressing either mouse GH (mGH) or control vector suspended in Matrigel (experiment 1) or in collagen (experiment 2). In experiment 1, after 8 weeks, palpable allografts in 8 of 9 mice injected with mGH-expressing fibroblasts were observed, compared with only 5 of 11 mice in the control group. In experiment 2, after 6 weeks, 4 of 11 mice injected with mGH-expressing fibroblasts formed palpable allografts, compared with no palpable grafts in the control group. Serum GH did not rise appreciably in allografted mice in experiment 1 (data not shown), but 4 mice with palpable allografts in experiment 2 had elevated serum GH concentrations (261–790 ng/mL). We confirmed strong immunoreactive mGH expression in all allografts tested (Figure 8A). Expression of both γH2AX (*P* = 0.0274; Figure 8, B–D) and Ki67 (*P* < 0.0001; Figure 8, E–G) was markedly increased in prostate tissue adjacent to grafts expressing mGH compared with vector control. In 6 of 9 animals in experiment 1 (Figure 8, H–O) and in 4 of 11 animals in experiment 2 (Supplemental Figure 9, A–D), we observed epithelial phenotypic features consistent with BPH in tissue adjacent to mGH-expressing fibroblasts. Prostate epithelial hyperplasia was not observed in prostate tissue adjacent to grafts expressing lentiV in control mice in either experiment.

8-Hydroxy-2′-deoxyguanosine (8-OH-dG), a marker of oxidative stress, has been shown to be induced in BPH (55–59). We also found that 8-OH-dG expression is enhanced in BPH lesions in mice injected with lenti-mGH–expressing fibroblasts (Supplemental Figure 9, E and F).

Human BPH and normal prostate, but not prostate cancer, shows positive nuclear p63 staining (60) and negative p504s (also called AMACR) staining (61, 62). Double staining with both antibodies revealed negative p504s and positive p63 staining, suggesting the hyperplastic lesions with mGH-expressing fibroblasts were consistent with a pathologic diagnosis of BPH (Figure 8, P–Q). Furthermore, Western blot analysis of the dorsolateral prostate carefully dissected to isolate prostate tissue adjacent to mGH-expressing or control fibroblasts showed that paracrine npGH emanating from the allografts increased DNA damage, as evidenced by increased γH2AX expression, and activated EMT, as evidenced by elevated TWIST1/2 and SNAI1 expression and reduced E-cadherin. In addition, epithelial proliferation in tissue adjacent to mGH-expressing fibroblasts was increased, as evidenced by PCNA activation (Figure 8R and Supplemental Figure 9G). In sum, the in vivo mouse studies reflected endocrine/paracrine GH-mediated prostate DNA damage and EMT that engendered epithelial changes consistent with the development of BPH.

## Discussion

The results show a heretofore unappreciated mechanism whereby age-related autocrine/paracrine local npGH induction mediated by increased DNA damage promotes BPH development.

Pituitary GH is involved in prostate development (63) and cell proliferation (6). Indeed, the prostate of transgenic mice universally overexpressing bovine GH is larger in weight compared with WT (64, 65). Although circulating GH declines with age in humans (66–69), we showed that age-associated DNA damage triggers npGH expression in both HPrECs and fibroblasts. In turn, GH induction further resulted in accumulation of DNA damage. These observations were confirmed by demonstrating increased local npGH and induced DNA damage in mice and in both normal aged prostate glands as well as in BPH tissue.

Our in vitro experiments here and our earlier observations in non-prostate tissue (2, 44, 70) show that DNA damage induces GH expression. Although in this study we compare tissue from 2 different zones, peripheral in “normal” samples and transitional in BPH samples, it is likely that mechanisms for GH induction are similar and GH detected in BPH is also induced by DNA damage.

Furthermore, murine prostate tissue exposed to paracrine GH emanating from GH-expressing fibroblast allografts exhibited increased DNA damage in vivo, while prostate DNA damage was attenuated in aged *GHR^–/–^* mice. These results are in agreement with our previous observations that no DNA damage accumulation was observed in the colon of *GHR^–/–^* mice (40). The role of DNA damage in the pathogenesis of human BPH has not been reported previously, although activation of DNA repair has been shown to yield age- and p53-dependent mouse prostate cell hypertrophy resembling human BPH (71). The results suggest that, by inducing DNA damage response, GH may facilitate a microenvironment favoring BPH development in older patients.

Analysis of RNA-seq in primary HPrECs treated with GH showed that genes comprising DNA repair pathways were suppressed. We validated these results using RT-PCR, and show that GH downregulates genes including *BRCC3*, which regulates DNA damage repair (72); *TADA3*, which enhances p53 stability (49); *NUDT16L1*, also known as Tudor-interacting repair regulator (TIRR), which restricts 53BP1 access to DNA breaks and its association with p53 (50, 51); *TAF5*, which increases transcription of p53 target genes involved in DNA damage repair (73); and *H4C16*, whose impairment leads to defective DNA repair (74, 75). We further validated these changes by showing decreased expression of BRCC3, TADA3L, and TAF5 proteins on Western blot in response to GH treatment. Together, these changes likely result in GH-induced defective DNA repair that enables DNA damage accumulation. These results are consistent with our earlier observations that both paracrine and endocrine GH attenuated DNA damage repair in 3-dimensional human intestinal organoids, leading to DNA damage accumulation (2, 3, 43). These results also suggest what we believe to be a novel mechanism for impaired prostate DNA repair caused by npGH and identify the prostate gland as a target for GHR signaling in aging normal tissue.

Oxidative stress is increased in BPH (55–59). Furthermore, ARR2PB-Nox4 mice with high levels of oxidative stress develop changes consistent with BPH (76), and in our experiments we also found increased 8-OH-dG expression in BPH lesions. Reactive oxygen species (ROS) generated by oxidative stress can induce both single-strand (SSB) and double-strand (DSB) DNA breaks (77, 78), and repair by both homologous recombination and non-homologous end joining create oxidative stress and ROS (79). It is likely that GH, as a metabolic hormone, increases both SSB/DSB and ROS. Therefore, increased γH2AX seen in our experiments likely reflects a cumulative picture of DNA damage.

The results suggest that DNA damage in aging tissue may induce GH consistent with our observations that etoposide significantly upregulates GH. Our previous results suggest that p53 induced in response to DNA damage activates the GH promoter (42). Furthermore, we also show that by inducing Wip1 and suppressing ATM phosphorylation, GH indeed suppresses DNA repair, resulting in unrepaired DNA damage accumulation. These findings are in agreement with our earlier finding in normal colon cells (2, 43). Therefore, the results imply that GH, induced in aging tissue by DNA damage, may lead to DNA damage accumulation and potentially chromosomal instability, exacerbating age-related genomic stress.

By inducing DNA damage, GH may activate prostate EMT. GH action on EMT has been documented in cancer models (80–83) and in non-tumorous renal podocytes (84), as well as in colon epithelial (40), corneal (85), and endothelial (86) cells. EMT is also activated by DNA damage (36, 38, 39), as well as by chromosome instability (87). γH2AX and TWIST1/2 expression, localized in the same area of normal prostate acini, were upregulated by both endocrine and local autocrine/paracrine GH, leading to E-cadherin suppression. It is likely that increased motility of prostate cells treated with GH, overexpressing npGH, or subjected to paracrine npGH results from induced EMT in the aging prostate.

EMT activation is a feature of prostate cancer (88) as well as other cancer types (89). However, EMT may also play a role in BPH pathogenesis (10, 42). EMT expression patterns were identified in human BPH epithelial tissue and in rat BPH and linked to luminal cell de-differentiation and new acini formation (90). In multilayered BPH ducts, E-cadherin is downregulated, whereas p-SMAD, SNAI1, and SNAI2 are induced (42). In our experiments, murine prostate tissue adjacent to GH-secreting allografts also showed activated EMT markers and decreased E-cadherin expression in agreement with our earlier studies (41, 70). The results shown here are consistent with previous reports that prostate DNA damage leads to EMT development, and our experiments show that both DNA damage and EMT are increased in BPH.

The question of whether EMT is involved in BPH development remains unclear. EMT may not result in a single mesenchymal state, but rather in a spectrum of intermediate states with degrees of epithelial and mesenchymal features (91). It is likely that in this cell-specific environment, EMT activation triggers cell proliferation and genomic instability, but not necessarily resulting in a fully mesenchymal phenotype. Another example of EMT activation in a benign tumor is pituitary adenoma, which almost never progresses to malignancy (89). Thus, our hypothesis is that EMT induced by GH and DNA damage may also be associated with BPH development, although this activation almost never leads to malignant transformation.

Our results suggest that aging of the normal non-cancerous prostate is associated with local npGH induction, enabling a pro-proliferative microenvironment. In our experimental models, we observed that both endocrine and local autocrine/paracrine GH increase normal prostate epithelial cell Ki67 or PCNA, which were inhibited by blocking GH signaling. Both endocrine and local IGF-1 may regulate prostate growth (63, 92, 93). We found that both endocrine and paracrine/autocrine GH stimulated local IGF-1 in normal prostate epithelial cells. Others have shown that IGF-1 suppresses EMT (94), whereas we found that both GH and IGF-1 triggered EMT. Importantly, unlike the effects of direct GH action, we show that IGF-1 significantly reduces DNA damage, likely resulting from its involvement in DNA repair (46). We therefore interpret these results as suggesting that GH regulation of prostate EMT is at least partially mediated by induced local IGF-1, yet GH also acts independently to activate the DNA damage response and promote BPH development in the aging prostate gland. Our results also show that in both normal aging prostate and in BPH, GH and γH2AX are equally induced compared to tissue derived from younger individuals, while TWIST2 is higher in BPH specimens. This reflects the effects of GH on DNA damage assessed by γH2AX, while TWIST2, associated with EMT, can be triggered by both GH and GH-induced IGF1. As 80%–90% of men after 70 years develop BPH, there is a strong possibility that GH, involved in both processes, plays a role. The results showing statistically significant correlations between GH and TWIST2, GH and γH2AX, and TWIST2 and γH2AX in BPH support our hypothesis.

We also cannot exclude that GH may activate the prolactin (PRL) receptor, due to conserved GHR homology (95, 96). Local PRL signaling has been suggested in BPH pathogenesis (97, 98), and PRL overexpression in the prostate (98), or experimental hyperprolactinemia (99), may lead to prostate hypertrophy. As we found that aged *GHR^–/–^* mice with normal PRL levels are characterized by a small prostate with low DNA damage, while WT mice exhibit age-associated increase in prostate weight, it is likely that our results could be attributed to GHR signaling.

There are several limitations of this study. We compared HPrECs derived from young donors to cells derived from BPH donors, and the history of these cells and growth conditions could be different. However, the consistent finding of increased GH protein expression in aging prostates as compared with young individuals supports our conclusion that GH is induced in aging prostate tissue and in BPH. We also appreciate the fact that using transplantation of GH-expressing fibroblasts may not fully replicate human BPH, as mouse pathology is not fully reflective of human BPH.

We show here that local paracrine npGH is induced in the aged prostate, enabling DNA damage accumulation and EMT activation to facilitate BPH development. The results demonstrate stronger expression of GH/γH2AX/TWIST2 in subgroups of aging individuals, which could have a prognostic value at biopsy. In addition to currently available therapies for BPH, use of a GHR antagonist, currently approved for acromegaly (100), may be a viable option for further clinical study.

## Methods

### Sex as a biological variable.

Our study exclusively examined male human cells and male mice because the prostate gland exists only in males.

### Mice.

*GHR^–/–^* mice were purchased from The Jackson Laboratory and heterozygous mice were used for breeding and backcrossed with C57BL/6 WT mice at least 6 times. WT and *GHR^–/–^* males were obtained from the same litters, and 4- to 6-month old and 24-month-old males used for experiments as indicated. Eight-week-old male C57BL/6 mice used for allograft studies were purchased from The Jackson Laboratory.

### Cells and treatments.

PNT2 (Sigma-Aldrich, 95012613, lot 15I070), an immortalized normal prostate epithelial cell line, was grown in RPMI 1640 (Gibco, 11875-093) with 10% FBS (GeminiBio, 100-106) and Antibiotic Antimycotic solution (Corning, 30-004-CI). HPrEC line 1 (ATCC, PCS-440-010), primary human non-tumorous prostate epithelial cells from a 24-year-old white male, was grown in Prostate Epithelial Cell Basal Medium (ATCC, PSC-440-030) with the Prostate Epithelial Cell Growth Kit (ATCC, PSC-440-040) (Full Prostate Media). HPrEC line 2 (Applied Biological Materials Inc., T4078, lot PL0211) from an unidentified donor was grown in Prigrow X Series Medium with supplements (Applied Biological Materials Inc., TM4078) with Antibiotic Antimycotic solution (Full Prigrow X Media). Flasks were pretreated with ECL Cell Attachment Matrix (Millipore, 08-110). NAFs were isolated from a prostatectomy specimen at Cedars-Sinai under institutional approval, as described previously (101).

Normal mouse fibroblasts were generated from the prostate of an 8-week-old WT C57BL/6 mouse, as described previously (102). Fibroblasts were grown in DMEM/F12 media (Corning, 10-090-CV) with 10% FBS, Antibiotic Antimycotic solution, and 10 μg/mL bovine insulin (Sigma-Aldrich, T6634; Full DMEM/F12 Media) and used in the first 10 passages.

For etoposide and BM001 treatments, cells were plated in appropriate Full Media. Etoposide (Millipore, E1383) was prepared as a 50 mM DMSO stock solution. BM001, developed and gifted by Specs Compound Handling, Zoetermeer, and University Medical Center Utrecht, was prepared as a 10 mM stock solution in DMSO (103). Control cells were treated with appropriate DMSO dilutions.

For GH treatments, cells were plated in Full Media, and changed the following day for media without Growth Kit or FBS, but with 0.1% BSA and indicated concentrations of recombinant human GH (R&D Systems, 1067-GH), reconstituted in PBS containing 0.1% BSA. Unlike circulating GH, which, during peak secretion, can reach 30–75 ng/mL (104, 105), GH in the medium decays rather rapidly (106). Therefore, higher doses were necessary to maintain adequate GH concentrations, as reported by others. Cells were treated with 50 or 100 ng/mL GH, both well-validated doses for in vitro experiments (107–113).

### BPH cell culture.

Primary BPH cell cultures were derived from 9 freshly isolated surgically resected human BPH specimens (Table 1). Tissue was dissociated in DMEM/F12 media with a mix of 1 mg/mL Collagenase (Sigma-Aldrich, C2674), 25 μg/mL hyaluronidase (Sigma-Aldrich, H3506), and DNase1 (1:1000; Zymo Research, E1009-A). Tissue was chopped and incubated at 37°C for 1.5 hours. Cell suspension was filtered through a 70 μm cell strainer (Falcon, 352350), rinsed, and plated in full Prostate Epithelial Cell Medium with 10 nM Rock inhibitor (Tocris, 1254) into a 24-well plate pretreated with ECL Cell Attachment Matrix. Medium was changed the following day to include 0.1% BSA but without the Growth Kit, and 100 ng/mL GH was added overnight as indicated.

### Mouse allografts.

Eight-week-old male C57BL/6 mice were used for prostatic orthotopic grafting, as described previously (101). Briefly, 6 × 10^5^ normal mouse prostate fibroblasts infected with lenti-mGH or lentiV as control were suspended in 50 μL Matrigel (experiment 1, Corning, 354234) diluted with PBS (1:1), or in 50 μL of type I collagen (experiment 2), and then injected into the anterior prostate lobes. Eight weeks (experiment 1) or 6 weeks (experiment 2) after the injection, allografts had created palpable masses, mice were sacrificed, and prostate tissue was analyzed. Earlier sacrifice in experiment 2 prevented excessive growth of the allografts, allowing dissection and collection of normal dorsolateral prostate tissue devoid of, but adjacent to, lenti-mGH or lentiV allografts for protein isolation. The remaining allografted prostate tissue was fixed in 10% buffered formalin for histological analysis.

### Cocultures.

See Supplemental Methods.

### Constructs and transfections.

Lentiviral particles expressing hGH (pLV-EF1p-hGH1-IRES-eGFP-WPRE) and respective control lentiviral particles (pLV-EF1p-mCherry-IRES-eGFP-WPRE), as well as murine GH (EF1-luc2-GH-Ubic) and vector (EF1-luc2-Ubic) were generated at the Regenerative Medicine Institute at Cedars-Sinai. Lentiviral particles expressing GHR shRNA (h) (sc-40015-V) and control shRNA (sc-108080) were purchased from Santa Cruz Biotechnology.

Cells were plated and the following day, 50 MOI of lentiviral particles were added together with 8 μg/mL polybrene (Santa Cruz Biotechnology, sc-134220). Medium was changed overnight, and cells split 48 hours later.

### Protein analysis.

For Western blot analysis, cells were either lysed in RIPA buffer (Cell Signaling Technology, 9806S) with protease inhibitors (MilliporeSigma, P8340), or isolated from TRIzol (Ambion, 15596018) and dissolved in 1% SDS (Molecular Research Center, Inc). Membranes were incubated overnight with antibodies, followed by corresponding secondary antibodies (anti-mouse IgG HRP linked from Amersham, NXA931; anti-rabbit HRP linked from Amersham, NA934; or anti-goat HRP linked from Jackson ImmunoResearch, 805-035-180). A Hikari signal enhancer kit was used (Nacalai, NU00102) to detect low-abundance proteins.

The following primary antibodies were used: GAPDH from Santa Cruz Biotechnology (catalog sc-32233) or from Cell Signaling (catalog 2118); β-actin from Sigma-Aldrich (catalog A1978); E-cadherin and IGF-1 from Cell Signaling Technology (catalog 3195 and 73034, respectively); TWIST2 from Santa Cruz Biotechnology (catalog sc-81417) or from Lifespan Biosciences (catalog LS-C416907); SNAI1 from R&D Systems (catalog AF3639) or LSBio (catalog LS-C176686); GH from R&D Systems (catalog AF 1067) or Lifespan Biosciences (catalog LS-B4199); GHR from Santa Cruz Biotechnology (catalog sc-137185), Abcam (catalog ab134078), or R&D Systems (catalog AF 1210); p53 from R&D Systems (against total protein, catalog AF 1355); γH2AX (p-histone H2AX, Ser139) from Cell Signaling Technology (catalog 9718); p-ATM (Ser1981, catalog 13050) and p-STAT5 (Tyr694, catalog 9359) from Cell Signaling Technology; Wip1 from Cell Signaling Technology (catalog 11901), Ki67 from Abcam (catalog ab15580); PCNA from Santa Cruz Biotechnology (catalog sc-53407); TAFIIp100 (TAF5) from Santa Cruz Biotechnology (catalog sc-376932); TADA3L from Santa Cruz Biotechnology (catalog sc-166119); and BRCC3 from Cell Signaling Technology (catalog 18215).

### Immunohistochemistry.

Human prostate tissue microarrays T191b, BNS19011, PR1002, PR1921c, and PR804 were purchased from TissueArray.com. All analyzed samples were marked “hyperplasia,” “normal,” or “adjacent normal.” “Normal” prostate tissue was obtained from autopsy of healthy individuals with information about prostate zone not available; “adjacent normal” samples were taken from at least 1.5 cm away from the border of a cancerous lesion and were most likely from the peripheral zone. Microarrays for age-associated changes ProsCA-1, ProsCA-2, ProsCA-3, and ProsCA-4 were generated at Cedars-Sinai Medical Center by the pathologist exclusively from normal non-tumorous peripheral zone tissue adjacent to cancer.

Staining of human tissue for GH (LS Bio, LS-B4199; 1:150), TWIST1/2 (Invitrogen, PA5-143850; 1:2000), γH2AX (Ser139) (Cell Signaling Technology, 9718; 1:500), and p-STAT5 (Tyr694) (Cell Signaling Technology, 9359; 1:100) was performed using an ImmPRESS Amplified Polymer Kit Peroxidase (Anti-Rabbit) (Vector Laboratories, MP-7601). Antigen retrieval for GH and γH2AX IHC was performed in citrate buffer, pH 6.0, and for TWIST1/2 and p-STAT5 in Tris-EDTA buffer, pH 9.0 (Abcam, ab93684).

Semiquantitative IHC analysis was performed using ImageJ (NIH). Staining intensity was determined as optical density (OD) of relevant areas with subtraction of OD of negative control (staining with omitted primary antibody), multiplied by the fraction of positive cells, and a score assigned to each sample. Human placenta was the positive control for GH and TWIST1/2 staining, and human tonsils was the positive control for γH2AX. Samples were considered positive if the staining intensity score was 40% or higher for prostate epithelium and 25% or higher for stroma compared with positive controls of GH and TWIST1/2 staining. For TWIST1/2 IHC, we included the cytoplasmic and whole-cell staining pattern for analysis. For γH2AX staining, samples were considered positive if the score was 20% or higher for prostate epithelium and 10% or higher for stroma compared with the positive control. Between 3 and 5 fields of several images were measured, and average values calculated.

IHC was performed on formalin-fixed, paraffin-embedded tissue derived from mouse prostate glands allografted with prostate fibroblasts overexpressing mGH or vector. Antibodies used for mouse IHC staining were goat anti-GH from R&D Systems (catalog AF1067; 1:25), Ki67 from Abcam (catalog ab15580; 1:1500), and γH2AX (Ser139) from Cell Signaling Technology (catalog 9718; 1:2000).

Double staining with p63 (Santa Cruz Biotechnology, sc-8343; 1:200) and P504S antibodies (Santa Cruz Biotechnology, sc-81710; 1:200) was performed using an ImmPRESS Duet Double Staining HRP/AP Polymer Kit (anti-mouse, brown; anti-rabbit, magenta) from Vector Laboratories (MP-7724-15).

Staining for 8-OH-dG (Bioss, BD10069184; 1:400) was performed as follows: after deparaffinization, tissue was permeabilized with 1% Triton X-100 for 30 minutes, washed, treated with 100 μg/mL RNase A (Promega, A797C) at 37°C for 1 hour, washed again and treated with 10 μg/mL of Proteinase K (Qiagen, 19131) for 30 minutes at 37°C, then treated with 4N HCL at room temperature for 7 minutes. After blocking in 2.5% horse serum, tissue was stained with anti–8-OH-dG antibodies diluted in 2.5% horse serum overnight at 4°C and detected using an ImmPRESS-AP horse anti-rabbit IgG Polymer Detection Kit (magenta, Vector Laboratories, MP-5401).

### Comet assay.

DNA damage in individual cells was quantified using a Comet Assay kit (OxiSelect, STA350) according to the manufacturer’s manual. Single-cell alkaline electrophoresis was performed for 30 minutes at 1 volt/cm. DNA damage was quantified by ImageJ as the percentage of DNA in the tail of the entire cell DNA (intensity of staining), multiplied by the length of the tail (Olive tail moment = tail DNA% × tail moment length).

### Cell migration and invasion assays.

See Supplemental Methods.

### Real-time PCR.

See Supplemental Methods.

### RNA-seq analysis.

RNA-seq was performed at the Cedars-Sinai Genomics Core. Cells derived from 2 separate lines (HPrEC line 1 and line 2) were treated with GH for 24 hours as described above. RNA-seq libraries were generated from RNA extracted using the RNeasy Micro kit (Qiagen) from cells, each with 3 biological replicate pairs comparing untreated and GH-treated conditions. Libraries were constructed using a strand-specific paired-end protocol following Illumina’s guidelines and sequenced on a NovaSeq X Plus platform with a read length of 2 × 100 bp. Sequencing reads were aligned to the human reference genome (GRCh38.113) using the STAR aligner ((https://github.com/alexdobin/STAR). Raw gene-level counts were quantified and normalized using the DESeq2 package in R (https://www.r-project.org/). Normalized counts were log-transformed, and fold changes calculated between GH-treated and untreated conditions for each replicate pair. Statistical significance was assessed using 2-sided Welch’s *t* tests, and *P* values adjusted for multiple comparisons using the Benjamini-Hochberg method to control the false discovery rate.

### Pathway enrichment analysis.

Over-representation analysis was performed using the clusterProfiler R package. Input gene lists consisted of genes that were consistently upregulated or downregulated in response to GH treatment across all replicate pairs in both cell lines. Enrichment analysis was conducted against multiple gene set collections from MSigDB (https://www.gsea-msigdb.org/gsea/msigdb), including Hallmark pathways, KEGG, Reactome, WikiPathways, and Gene Ontology categories: Biological Process, Cellular Component, and Molecular Function.

### Statistics.

Results are expressed as mean ± SEM. Comparison of means between 2 groups was performed by 2-tailed Student’s *t* test. For continuous data for 3 or more groups, 2-way ANOVA followed by Tukey’s test was used to correct for multiple group comparisons. Associations between categorical variables were compared using simple linear regression and χ^2^ tests. GraphPad Prism v8 software was used for analysis. A *P* value of less than 0.05 was considered significant.

### Study approval.

Generation of primary cell cultures obtained from human patient donors with BPH at Cedars-Sinai was approved by the Cedars-Sinai Medical Center Institutional Review Board (IRB 20577). Written informed consent was received from each patient donor prior to participation. Animal procedures were performed according to an approved protocol from the Cedars-Sinai Medical Center Institutional Animal Care and Use Committee (IACUC 007440 and 009252).

### Data availability.

Values for all data points in graphs are reported in the Supporting Data Values file. RNA-seq data are deposited in the NCBI GEO repository with accession number GSE298387.

## Author contributions

VC and SM developed the hypothesis and designed the research. MR, SZ, NAB, VC, and SM wrote the manuscript. MR, SZ, and SB conducted experiments. MR, SZ, SKKM, NAB, SJF, HLK, VC, and SM analyzed, discussed, and interpreted the data. VC and SM coordinated and directed the project. All authors approved the submitted manuscript.

## Conflict of interest

The authors have declared that no conflict of interest exists.

## Funding support

This work is the result of NIH funding, in whole or in part, and is subject to the NIH Public Access Policy. Through acceptance of this federal funding, the NIH has been given a right to make the work publicly available in PubMed Central.

- NIH grant R01DK113998 (to SM).
- Doris Factor Molecular Endocrinology Laboratory at Cedars-Sinai (to SM).

## Supplementary Material

Supplemental data

Unedited blot and gel images

Supporting data values

## Figures and Tables

**Table 1:**
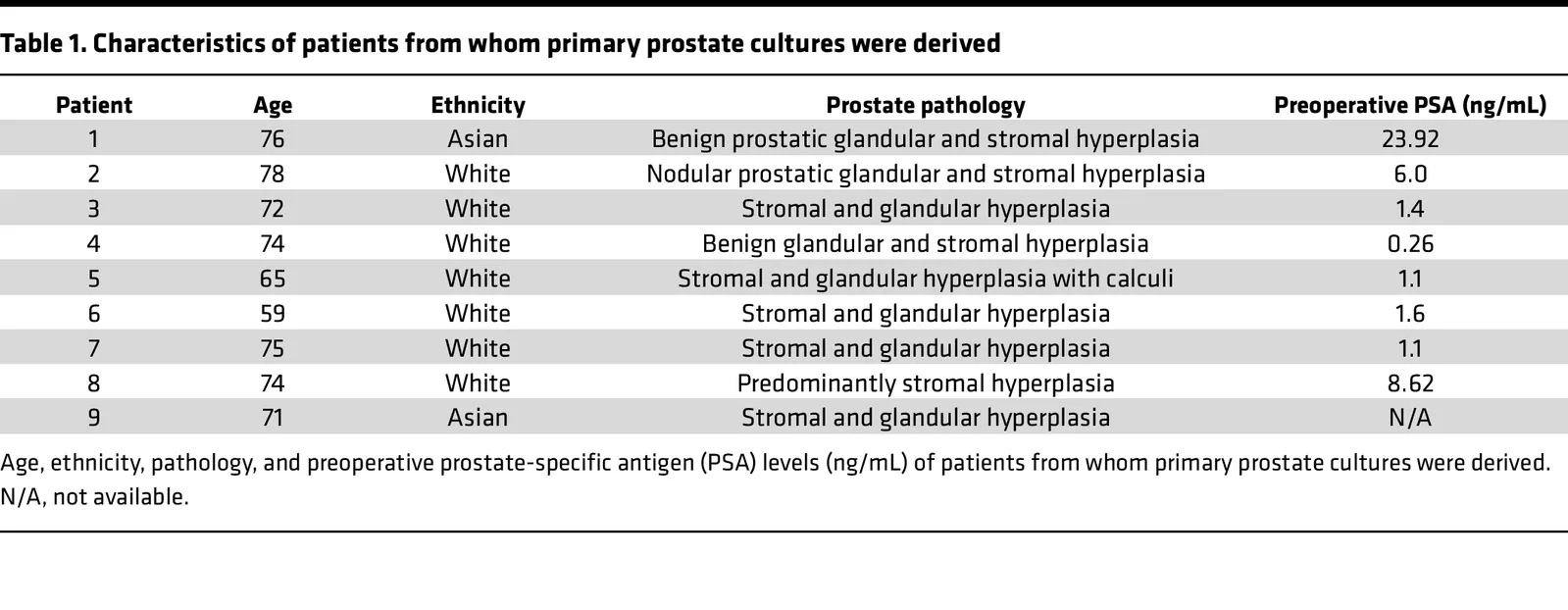
Characteristics of patients from whom primary prostate cultures were derived

## Version 1 07/28/2026

In-Press Preview

## Version 2 09/08/2026

Electronic publication
