# Supplementary material for: Local growth hormone promotes benign prostatic hyperplasia
Source: JCI Insight. 2026 Jul 28;11(17):e206012. doi: 10.1172/jci.insight.206012 (PMC13564083; doi:10.1172/jci.insight.206012)
Supplement: Supplementary file 1 [file jciinsight-11-206012-s160.pdf]

Local Growth Hormone Promotes Benign Prostatic Hyperplasia
Supplemental Materials

Supplemental Methods

Supplemental Figures

Supplemental Methods

Co-cultures

For co-culture of HPrEC line 1 or PNT2 with lentiGH- or lentiV-infected NAF, 3×10^5 fibroblasts per well were plated into a 6-well plate in complete DMEM/F12. The following day, fibroblast medium was changed to serum free DMEM/F12 supplemented with 0.1% BSA. Simultaneously, 5×10^5 HPrEC line 1 or PNT2 cells were plated in a separate plate in complete medium in 0.4 µm Transwell Permeable 24 mm Inserts (Costar, cat#3412). After 24 hours, medium was changed to include 0.1% BSA without Growth Kit or FBS, and inserts were placed into wells with fibroblasts. After 24 hours of co-culture, cells were collected. Medium hGH was measured by ELISA (ALPCO, cat#25-HGHHU-E01).

*Cell Migration and Invasion Assay*s

For migration or invasion experiments, 1×10^5 PNT2 lentiGH or lentiV cells were plated in 200 µL serum free RPMI1640 medium with 0.1% BSA into Transwell Migration Chambers (Corning, cat#354578, pore size 8 M) or into Corning BioCoat Growth Factor Reduced Matrigel Invasion Chambers (Corning, cat#354483). Chambers were placed into 750 µL of full RPMI1640 medium. After 24 hours, cells were fixed in 70% Ethanol, stained with 0.1% Crystal Violet, and non-migrated/non-invaded cells removed from the chambers with cotton swabs. Images were taken at 100x magnification (at least 6 images from each chamber), and migrated or invaded cells counted.

In co-culture experiments, 1×10^5 NAF lentiGH or lentiV cells/well were plated into the bottom of 24-well plates in 1 mL full DMEM/F12 medium. After 2 days, Transwell Migration or Invasion chambers were placed into these plates together with 1×10^5 intact PNT2 or HPrEC line 1 cells in 200 µL serum-free RPMI1640 or Prostate medium without Growth Kit but with 0.1% BSA.

In experiments with GH treatments, 1×10^5 HPrEC line 1, HPrEC line 2, or PNT2 cells were plated into migration chambers in 200 µL of appropriate media without Growth Kit or FBS but with 0.1% BSA. Chambers were placed inside 24-well plates with 1 mL full medium, appropriate for each cell type, and with 100 ng/mL human GH. The number of migrated or invaded cells was analyzed 24 hours later.

Real-Time PCR

Total RNA was isolated with TRIzol (Ambion, cat#15596018) followed by RNAeasy mini Kit (Qiagen). cDNA was synthesized from 1 µg purified RNA by the SuperScript II First-Strand cDNA synthesis system (Thermo Fisher). Quantitative PCR was performed in 20 µL reactions using IQ SYBR Green Master Mix in BioRad IQ5 instrument (BioRad Laboratories). The following PrimePCR Assays for human mRNA were used: actin, GAPDH, TWIST2, BRCC3, Hist4H4, NUDT16L1, TADA3, and TAF5 (all from BioRAD, cat#10025636), TWIST1, SNAI1, GHR and hGH1 (all from Qiagen, cat#PPH02132A, PPH02459B, PPH02641A, and PPH005778B, respectively). Experiments were performed in triplicate. Relative mRNA quantities in experimental samples were determined by CFX Maestro 1.0 software expressed in arbitrary units as fold-difference from control.

A

	GH in epithelium		
	positive	negative	total
21-39 yrs	1	45	46
40-60 yrs	5	37	42
61-83 yrs	17	67	84
Total	23	149	172
$\chi^2(2, N=172)=8.4761$, $p=0.014435$			

B

	GH in stroma		
	positive	negative	total
21-39 yrs	2	44	46
40-60 yrs	6	36	42
61-83 yrs	17	67	84
Total	25	147	172
$\chi^2(2, N=172)=6.0$, $p=0.049$			

C

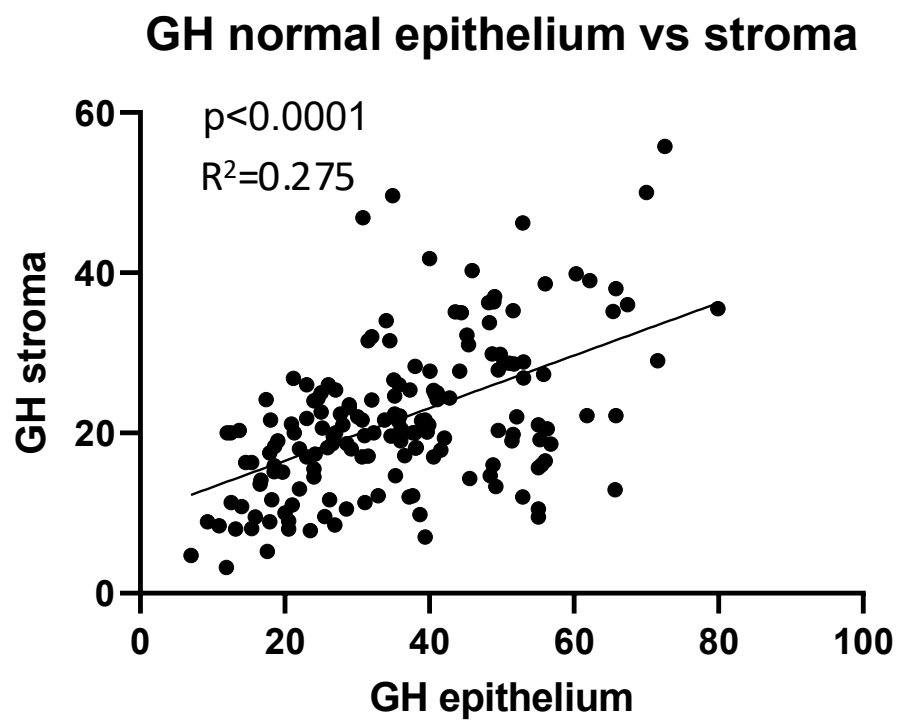

D

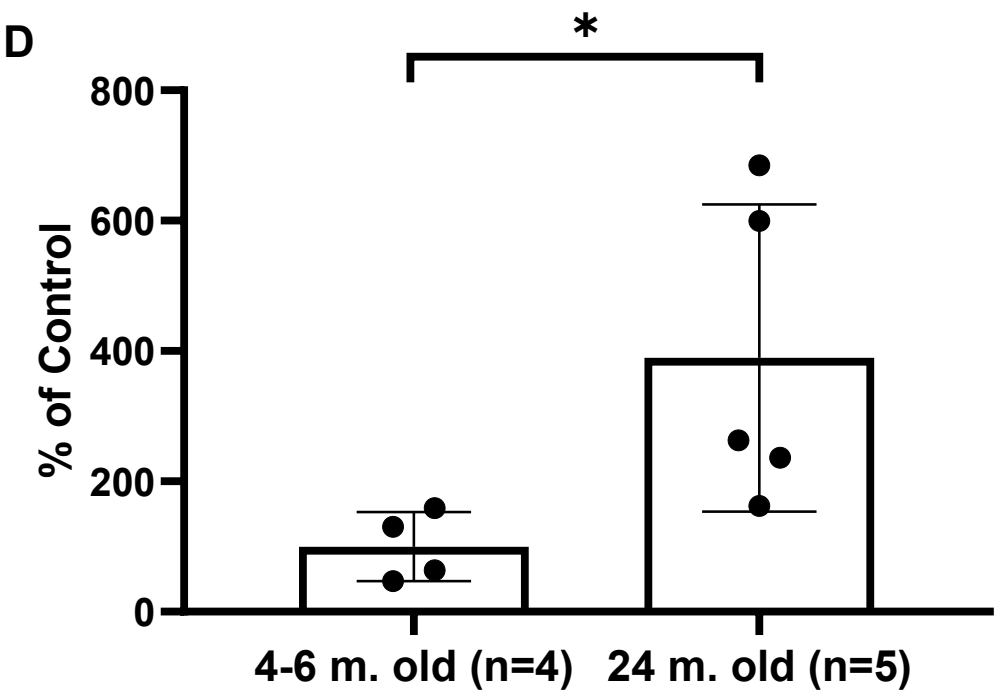

E

	γH2AX in epithelium		
	positive	negative	total
21-39 yrs	6	32	38
40-60 yrs	10	14	24
61-83 yrs	23	22	45
Total	39	68	107
$\chi^2(2, N=107)=11.4604$, $p=0.003246$			

F

	γH2AX in stroma		
	positive	negative	total
21-39 yrs	3	35	38
40-60 yrs	6	18	24
61-83 yrs	15	30	45
Total	24	83	107
$\chi^2(2, N=107)=7.7802$, $p=0.020444$			

G

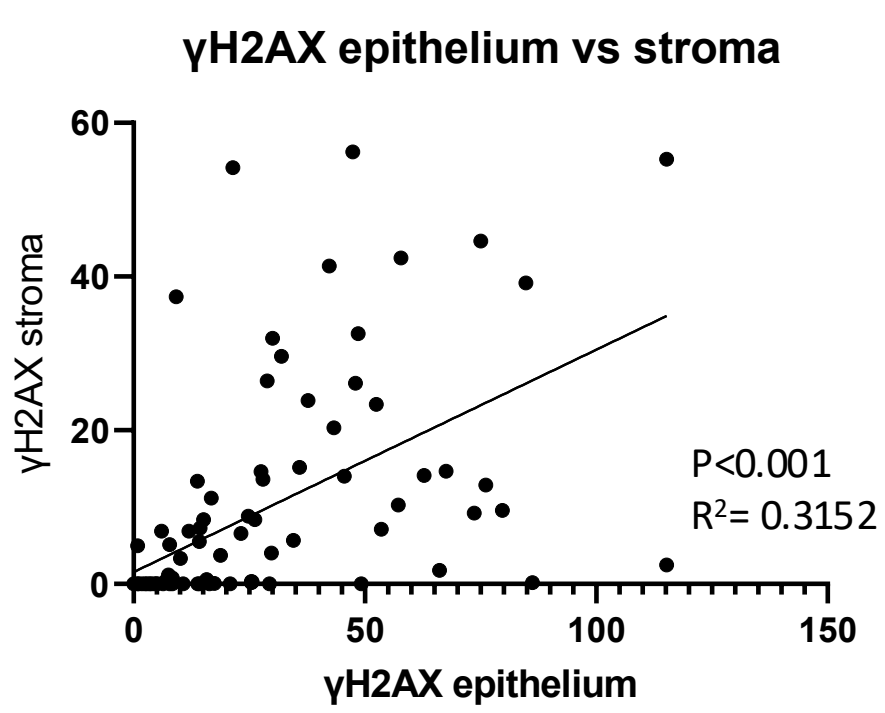

Supplemental Figure 1. Prostate npGH expression increases and DNA damage accumulates with age. (A-B) Number of prostate specimens **(A)** epithelial cells and **(B)** stroma scored positive or negative for GH expression in each age group. Differences between groups assessed by chi-squared test. **(C)** Correlation between epithelial and stromal GH expression in normal prostate tissue analyzed by simple linear regression. **(D)** ImageJ quantification of GH expression normalized to loading controls. Each dot represents individual 4-6-month-old or 24-month-old WT male mouse. Results are shown as mean ± SEM for each age group. Data are graphed as percent of control, but statistical testing was performed on raw numbers. Differences were assessed with 2-tailed t test. *$p<0.05$. **(E-F)** Number of prostate specimens **(E)** epithelial cell and **(F)** stroma scored positive or negative for γH2AX expression staining in each age group. Differences between groups assessed by chi-squared test. **(G)** Correlation between epithelial and stromal γH2AX expression in normal prostate tissue analyzed by simple linear regression.

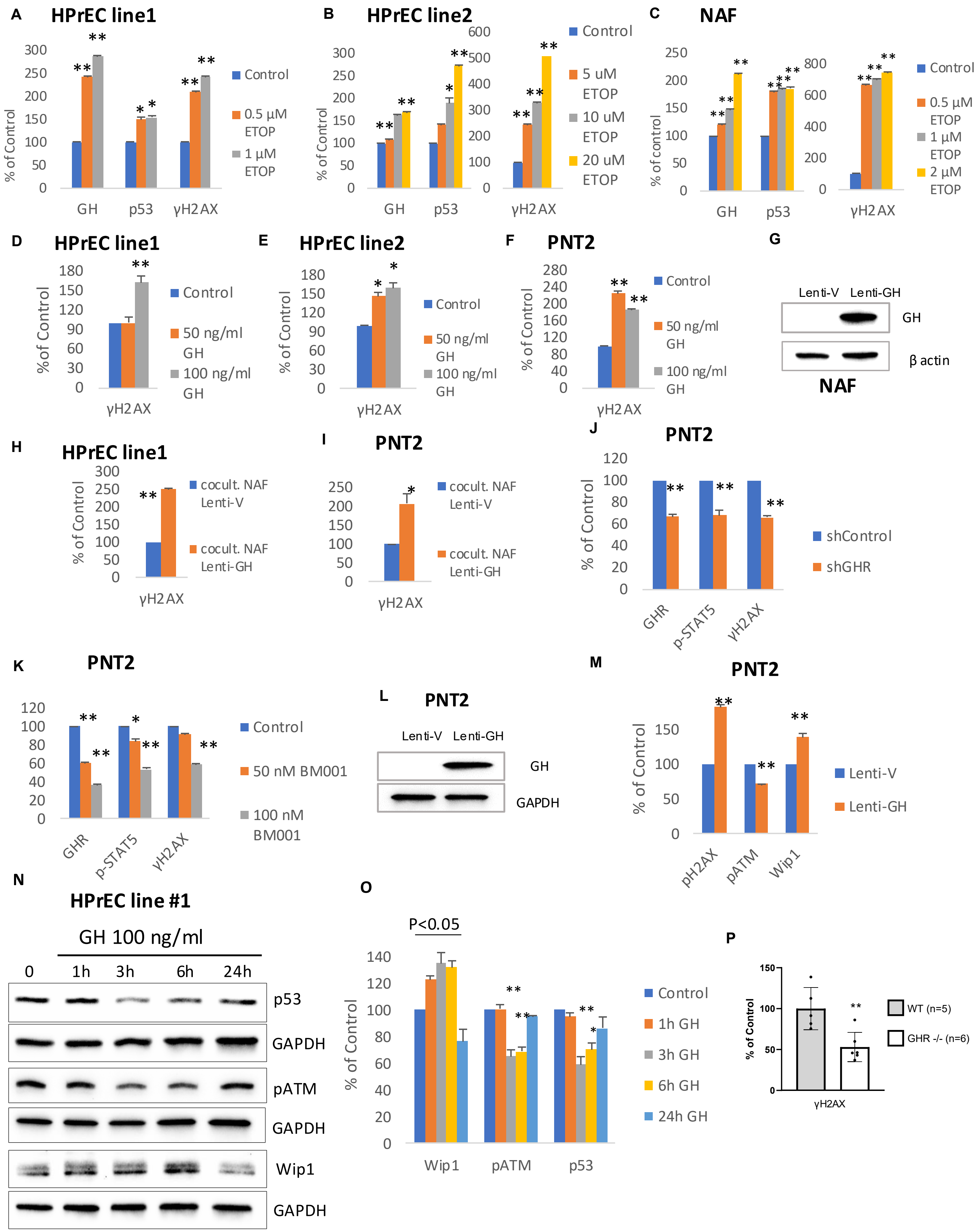
A
HPrEC line1
% of Control
Control
0.5 µM ETOP
1 µM ETOP
GH
p53
γH2AX
B
HPrEC line2
% of Control
Control
5 uM ETOP
10 uM ETOP
20 uM ETOP
C
NAF
% of control
Control
0.5 µM ETOP
1 µM ETOP
2 µM ETOP
D
HPrEC line1
Control
50 ng/ml GH
100 ng/ml GH
E
HPrEC line2
F
PNT2
G
Lenti-V
Lenti-GH
GH
β actin
NAF
H
HPrEC line1
cocult. NAF Lenti-V
cocult. NAF Lenti-GH
I
PNT2
J
PNT2
shControl
shGHR
GHR
p-STAT5
K
PNT2
Control
50 nM BM001
100 nM BM001
L
PNT2
GAPDH
M
PNT2
pH2AX
pATM
Wip1
N
HPrEC line #1
GH 100 ng/ml
0
1h
3h
6h
24h
O
P<0.05
1h GH
3h GH
6h GH
24h GH
P
WT (n=5)
GHR -/- (n=6)

Supplemental Figure 2. Prostate cell DNA damage triggers npGH, which further enhances prostate DNA damage accumulation. (A-F) ImageJ quantification of protein expression normalized to loading controls. **(A)** HPrEC line 1, **(B)** HPrEC line 2, and **(C)** NAF were treated with indicated doses of etoposide (ETOP) for 24 h.. **(D-F)** Cells were treated with indicated GH doses for 24 hours**.** (**D**)HPrEC line 1, **(E)** HPrEC line 2, and **(F)** PNT2. For D-F, experiments were repeated 4 times. **(G)** NAF were infected with lentiGH or lentiV and analyzed 4 weeks later. Western blot confirming GH expression in lentiGH infectants. **(H,I)** Cells were co-cultured with NAF infected with lentiGH or lentiV for 24 hours. ImageJ quantification of γH2AX expression normalized to loading controls in **(H)** HPrEC line 1 and **(I)** PNT2. In (**H**), experiments were repeated 2 times; (**I**), experiments were repeated 6 times. **(J)** ImageJ quantification of protein expression in PNT2 infected with lentivirus expressing control shRNA (shControl) or GHR shRNA (shGHR) and analyzed after 4 days. **(K)** ImageJ quantification of protein expression in PNT2 treated with indicated doses of BM001 and analyzed 6 hours later. (**L,M**) PNT2 were infected with lentiGH or lentiV and analyzed 4 weeks later. (**L**) Western blot confirming GH expression in lentiGH. (**M**) ImageJ quantification of γH2AX, pATM, and Wip1 expression normalized to loading controls. **(N)** Western blot and **(O)** ImageJ quantification of DNA damage proteins in HPrEC cells line #1 treated with GH.Each experiment was repeated 4 times. (**P**)Average measurements of ImageJ quantification of prostate γH2AX expression in 24-month-old WT (n=5) and GHR-/- (n=6) male mice. Each dot represents sample analysis derived from an individual animal. For graphs, results are shown as mean ± SEM for each groupResults are graphed as percent of control, but statistical testing was performed on raw numbers. For A-F, K, and O, differences were assessed with ANOVA followed by Tukey's post-hoc test to adjust for multiple comparisons. For H, I, J,P, differences were assessed with 2-tailed t test. *$p<0.05$, **$p<0.01$ versus control.

A

	TWIST1/2 in epithelium		
	positive	negative	total
21-39 yrs	2	36	38
40-60 yrs	4	20	24
61-83 yrs	14	32	46
Total	20	88	108
$\chi^2(2, N=108)=8.8$, p=0.012			

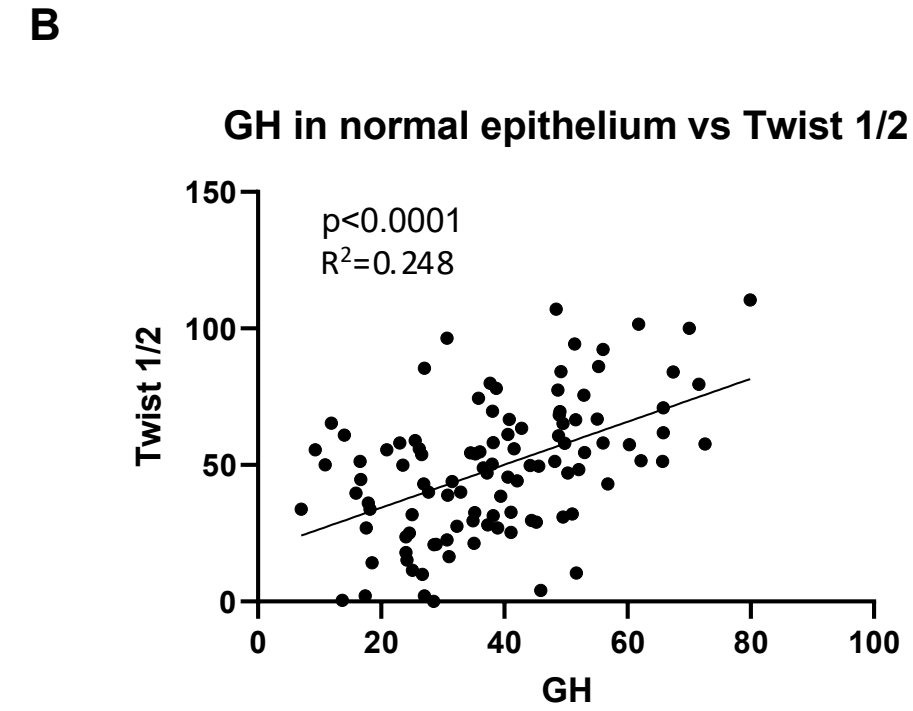

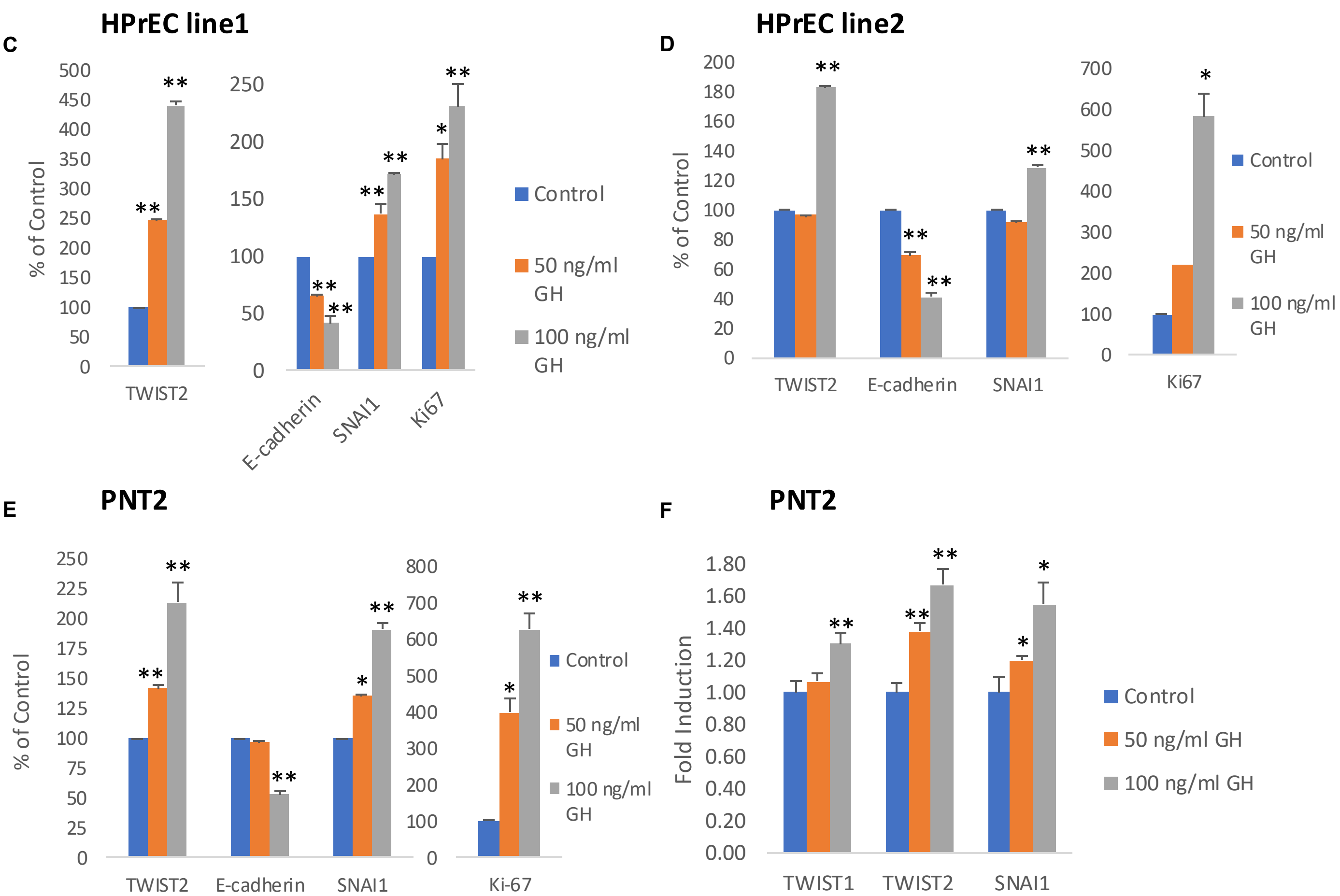

Supplemental Figure 3. (A-B) Normal human prostate TWIST1/2 expression increases with age. (A) Number of prostate specimens epithelial cell scored positive or negative for TWIST1/2 expression staining in each age group. Differences between groups assessed by chi-squared test. **(B)** Correlation between TWIST1/2 expression and GH in normal prostate epithelial cells analyzed using simple linear regression. Each dot represents IHC staining intensity of individual patient. **(C-F) Endocrine GH induces prostate cell EMT and proliferation.** ImageJ quantification of protein expression normalized to loading controls in **(C)** HPrEC line 1, **(D)** HPrEC line 2, and **(E)** PNT2 treated with indicated doses of GH for 24 hours. Each experiment was repeated 2-4 times. Results are shown as mean ± SEM. Data are graphed as percent of control, but statistical testing was performed on raw numbers. **(F)** Representative real-time PCR of mRNA levels in PNT2 treated with indicated doses of GH for 24 hours. Results are expressed as fold-change versus control. Results are depicted as mean ± SEM of triplicates. Differences were assessed with ANOVA followed by Tukey's post-hoc test to adjust for multiple comparisons. *p<0.05, **p<0.01 versus control.

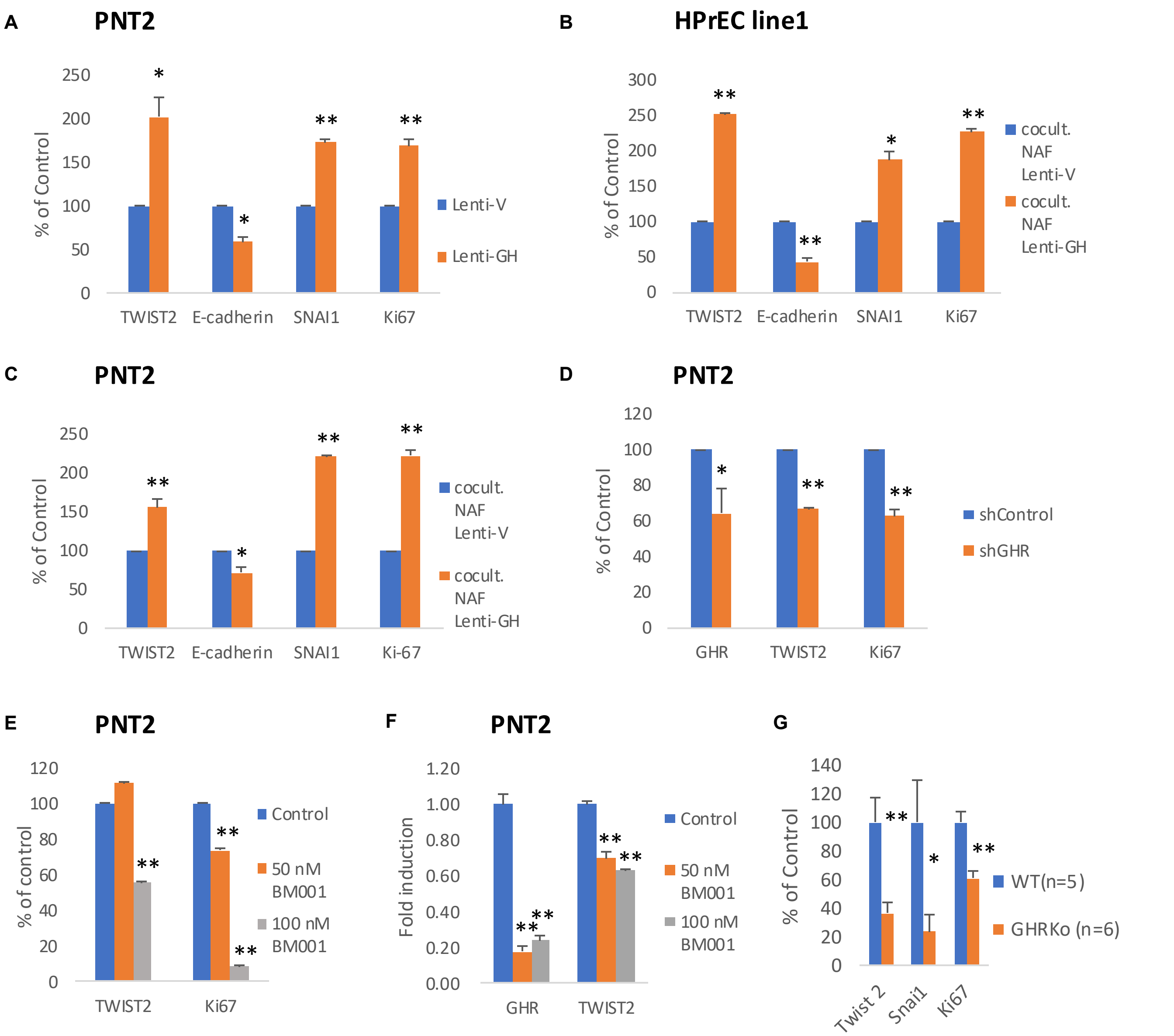

Supplemental Figure 4. GH induces prostate EMT and proliferation. ImageJ quantification of protein expression normalized to loading controls. **(A)** PNT2 infected with lentiGH or lentiV and analyzed 4 weeks later. Experiments for TWIST2, E-cadherin, and SNAI1 were repeated twice, and Ki67 4 times. **(B-C)** **(B)** HPrEC line 1 and **(C)** PNT2 co-cultured with lentiGH- or lentiV-infected NAF for 24 hours. For B, experiments were repeated 4 times. For C, experiments for TWIST2, SNAI1, and Ki67 were repeated 4 times, and for E-cadherin 2 times. **(D)** PNT2 infected with lentivirus expressing control shRNA (shControl) or GHR shRNA (shGHR). Experiments were repeated 4 times. **(E)** PNT2 treated with BM001 and analyzed 6 hours later. Experiments were repeated 2 times. For A-E, results are shown as mean ± SEM. **(F)** Representative real-time PCR of mRNA levels in PNT2 treated for 6 hours with indicated doses of BM001. Results are expressed as fold-change versus control. Results are shown as mean ± SEM of triplicates. **(G)** Prostate protein expression in tissues derived from 24-month-old WT and *GHR*$^{-/-}$ male mice. Average measurements of 5 WT and 6 *GHR*$^{-/-}$ mice are shown. Each dot represents a sample derived from an individual animal. Results are shown as mean ± SEM of each group. Data are graphed as percent of control, but statistical testing was performed on raw numbers. For A-D and G, differences were assessed with 2-tailed t test. For E and F, differences were assessed with ANOVA followed by Tukey's post-hoc test to adjust for multiple comparisons. *p<0.05, **p<0.01 versus control.

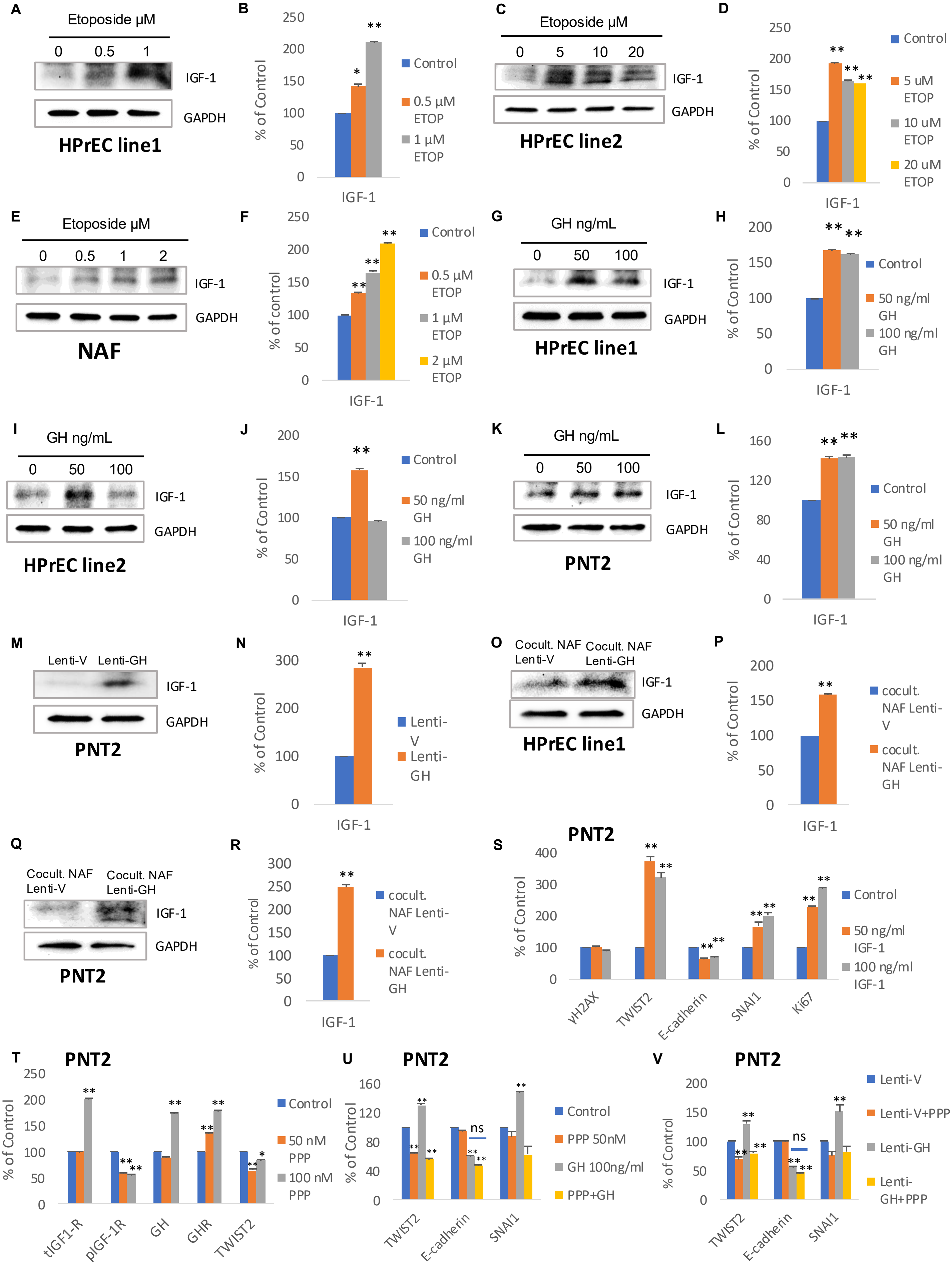
A
Etoposide μM
0 0.5 1
IGF-1
GAPDH
HPrEC line1
B
% of Control
250 200 150 100 50 0
**
*
Control
0.5 μM ETOP
1 μM ETOP
IGF-1
C
Etoposide μM
0 5 10 20
IGF-1
GAPDH
HPrEC line2
D
% of Control
250 200 150 100 50 0
** ** **
Control
5 uM ETOP
10 uM ETOP
20 uM ETOP
IGF-1
E
Etoposide μM
0 0.5 1 2
IGF-1
GAPDH
NAF
F
% of control
250 200 150 100 50 0
** ** **
Control
0.5 μM ETOP
1 μM ETOP
2 μM ETOP
IGF-1
G
GH ng/mL
0 50 100
IGF-1
GAPDH
HPrEC line1
H
% of Control
200 150 100 50 0
** **
Control
50 ng/ml GH
100 ng/ml GH
IGF-1
I
GH ng/mL
0 50 100
IGF-1
GAPDH
HPrEC line2
J
% of Control
200 150 100 50 0
**
Control
50 ng/ml GH
100 ng/ml GH
IGF-1
K
GH ng/mL
0 50 100
IGF-1
GAPDH
PNT2
L
% of Control
160 120 80 40 0
** **
Control
50 ng/ml GH
100 ng/ml GH
IGF-1
M
Lenti-V Lenti-GH
IGF-1
GAPDH
PNT2
N
% of Control
300 200 100 0
**
Lenti-V
Lenti-GH
IGF-1
O
Cocult. NAF Lenti-V
Cocult. NAF Lenti-GH
IGF-1
GAPDH
HPrEC line1
P
% of Control
200 150 100 50 0
**
cocult. NAF Lenti-V
cocult. NAF Lenti-GH
IGF-1
Q
Cocult. NAF Lenti-V
Cocult. NAF Lenti-GH
IGF-1
GAPDH
PNT2
R
% of Control
300 250 200 150 100 50 0
**
cocult. NAF Lenti-V
cocult. NAF Lenti-GH
IGF-1
S
PNT2
% of Control
400 300 200 100 0
Control
50 ng/ml IGF-1
100 ng/ml IGF-1
γH2AX TWIST2 E-cadherin SNAI1 Ki67
T
PNT2
% of Control
250 200 150 100 50 0
Control
50 nM PPP
100 nM PPP
tIGF1-R pIGF-1R GH GHR TWIST2
U
PNT2
% of Control
160 120 80 40 0
ns
Control
PPP 50nM
GH 100ng/ml
PPP+GH
TWIST2 E-cadherin SNAI1
V
PNT2
% of Control
200 150 100 50 0
ns
Lenti-V
Lenti-V+PPP
Lenti-GH
Lenti-GH+PPP
TWIST2 E-cadherin SNAI1

Supplemental Figure 5. Prostate IGF-1 increases with GH and DNA damage, induces EMT and proliferation, but attenuates DNA damage. Western blots and ImageJ quantification of protein expression normalized to loading controls. **(A-F)** Treatment of **(A-B)** HPrEC line 1, **(C-D)** HPrEC line 2, and **(E-F)** NAF with indicated doses of etoposide for 24 hours. **(G-L)** Treatment of **(G-H)** HPrEC line 1, **(I-J)** HPrEC line 2, and **(K-L)** PNT2 with indicated doses of GH and analyzed 24 hours later. **(M-N)** PNT2 infected with lentiGH or lentiV and analyzed 4 weeks later. **(O-R)** Co-culture of **(O-P)** HPrEC line 1 and **(Q-R)** PNT2 with lentiGH- or lentiV-infected NAF for 24 h. **(S-U)** Treatment of PNT2 with indicated doses of **(S)** IGF-1, **(T)** PPP, or **(U)** PPP with or without GH and analyzed 24 hours later. tIGF-R, total IGF-1 receptor; pIGF-1R, phosphorylated IGF-1 receptor. **(V)** PNT2 infected with lentiGH or lentiV, treated with indicated doses of PPP after 4 weeks, and analyzed 24 hours later. All experiments were repeated 2-4 times. For B, D, F, H, J, L, N, P, R, S-V, results are shown as mean ± SEM. Data are graphed as percent of control, but statistical testing was performed on raw numbers. For B, D, F, H, J, L, S-V, differences were assessed with ANOVA followed by Tukey's post-hoc test to adjust for multiple comparisons. For N, P, R, differences were assessed with 2-tailed t test. *$p<0.05$, **$p<0.01$ versus control.

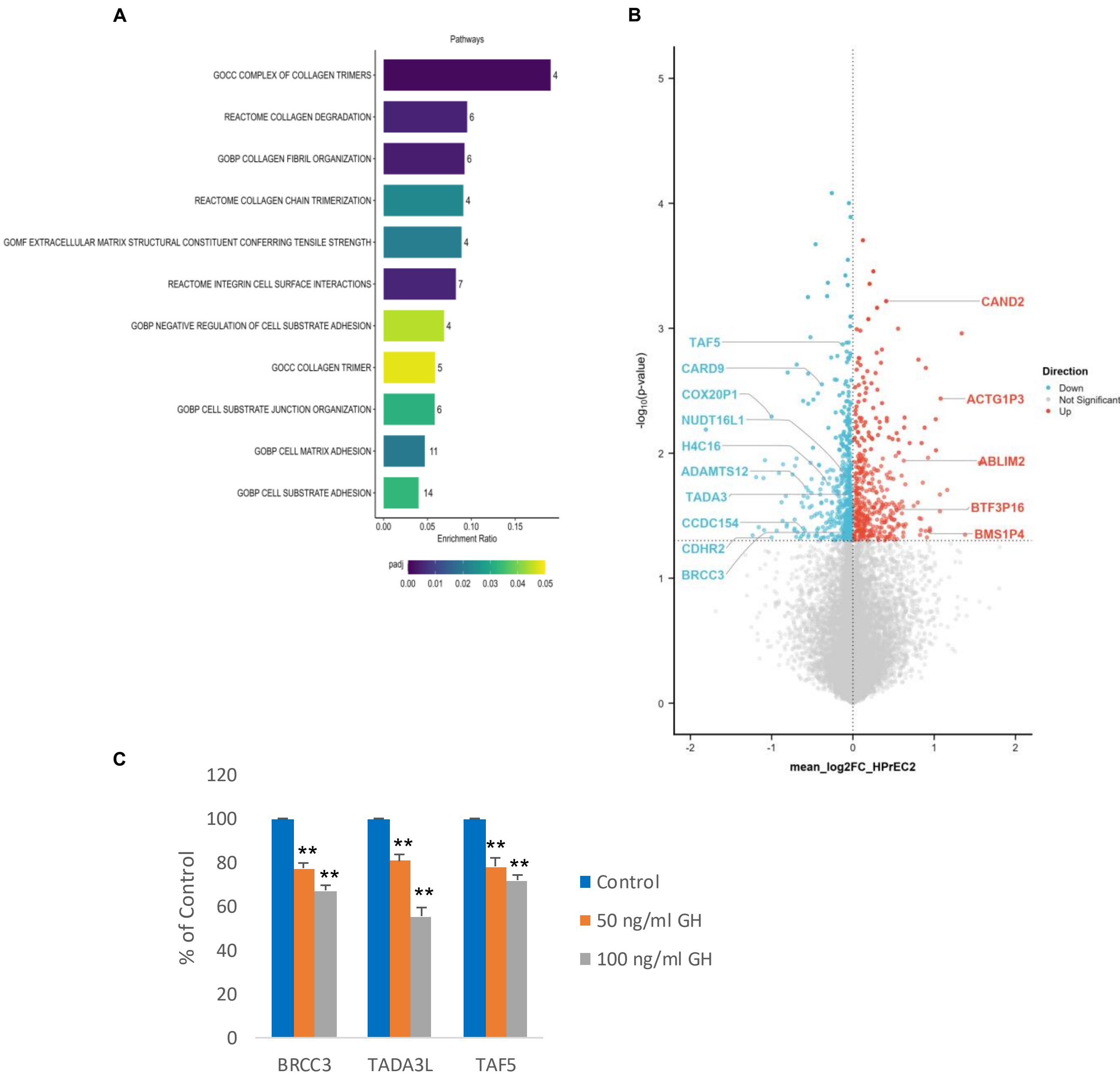

Supplemental Figure 6. GH suppresses DNA damage repair pathways in human prostate epithelial cells. (A,B) HPrEC cells were treated with 100 ng/ml GH for 24 hours. **(A)** Barplot showing collagen-related pathways identified by over-representation analysis. X axis (enrichment ratio) represents the ratio of differentially expressed genes (DEGs) within a specific pathway to the total number of genes annotated to that pathway. Number of DEGs within each pathway is given next to its respective bar. P value is indicated by color of the bar. **(B)** Combined volcano plot of differential gene expression. **(C)** ImageJ quantification of protein expression normalized to loading control in HPrEC line 2 treated with indicated doses of GH for 24 hours. Results are shown as mean ± SEM of at least 4-6 independent experiments. Data are graphed as percent of control, but statistical testing was performed on raw numbers. Differences were assessed with ANOVA followed by Tukey's post-hoc test to adjust for multiple comparisons. **$p<0.01$ versus control..

A

	GH in epithelium		
	positive	negative	total
61-83 yrs	17	67	84
BPH(61-88 yrs)	18	76	94
Total	35	143	178
χ^2(1,N=178)=0.0333, p=0.855177			

B

	GH in stroma		
	positive	negative	total
61-83 yrs	17	67	84
BPH(61-88 yrs)	15	79	94
Total	32	146	178
χ^2(1,N=178)=0.5512, p=0.45781			

C

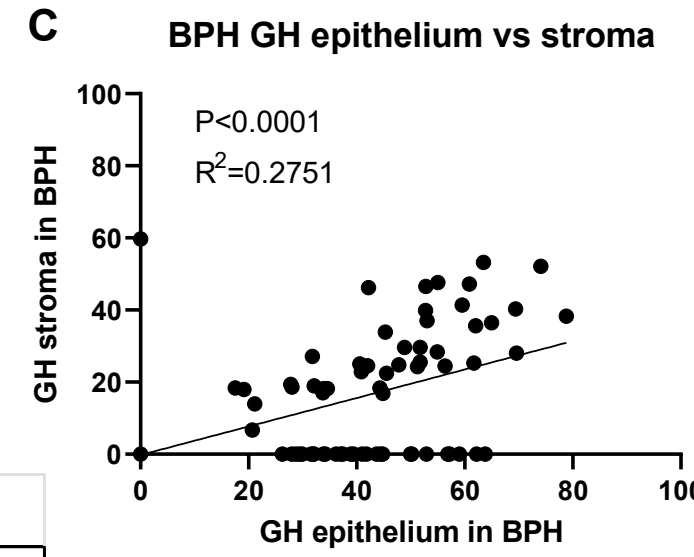

D

	γH2AX in epithelium		
	positive	negative	total
61-83 yrs	23	22	45
BPH(61-88 yrs)	32	44	76
Total	55	66	121
χ^2(1,N=121)=0.9246, p=0.33627			

E

	γH2AX in stroma		
	positive	negative	total
61-83 yrs	15	30	45
BPH(61-88 yrs)	18	58	76
Total	31	90	121
χ^2(1,N=121)=2.2369, p=0.134748			

F

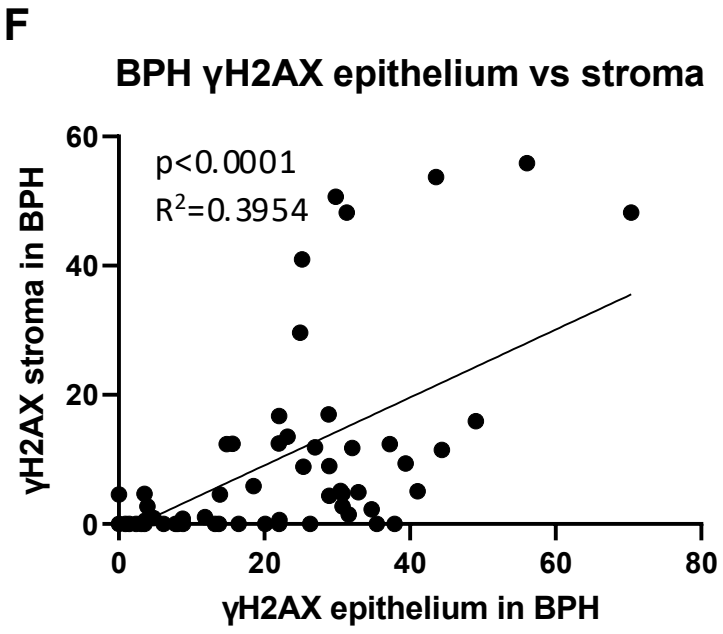

G

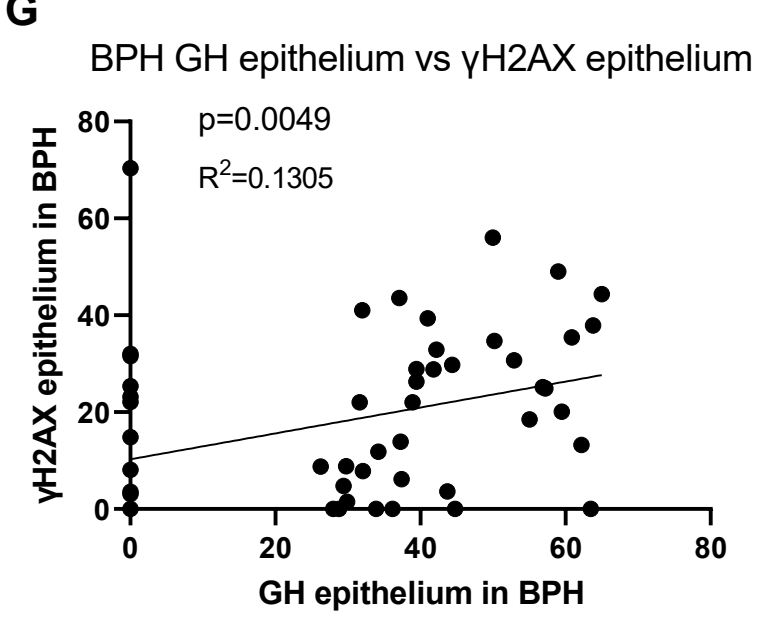

H

	TWIST1/2 in epithelium		
	positive	negative	total
61-83 yrs	14	32	46
BPH(61-88 yrs)	48	51	99
Total	62	83	145
χ^2(1,N=145)=4.1807, p=0.040887			

I

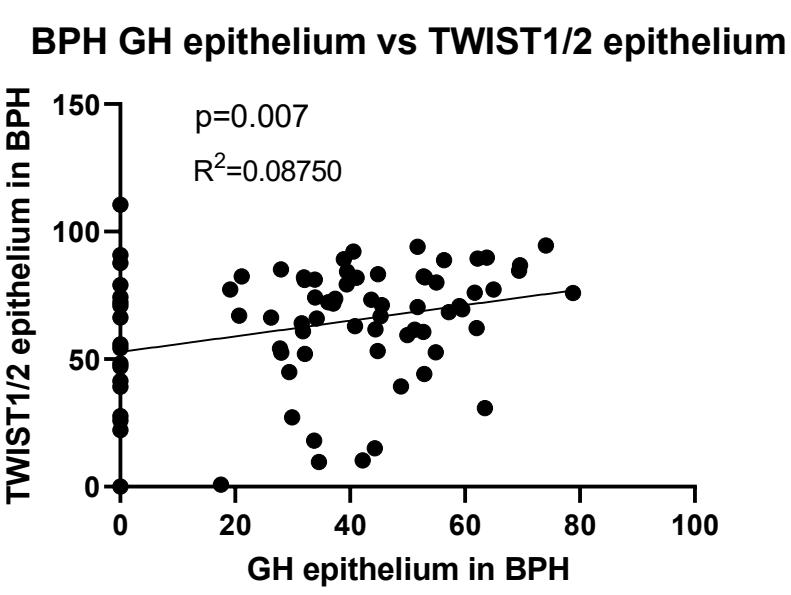

J

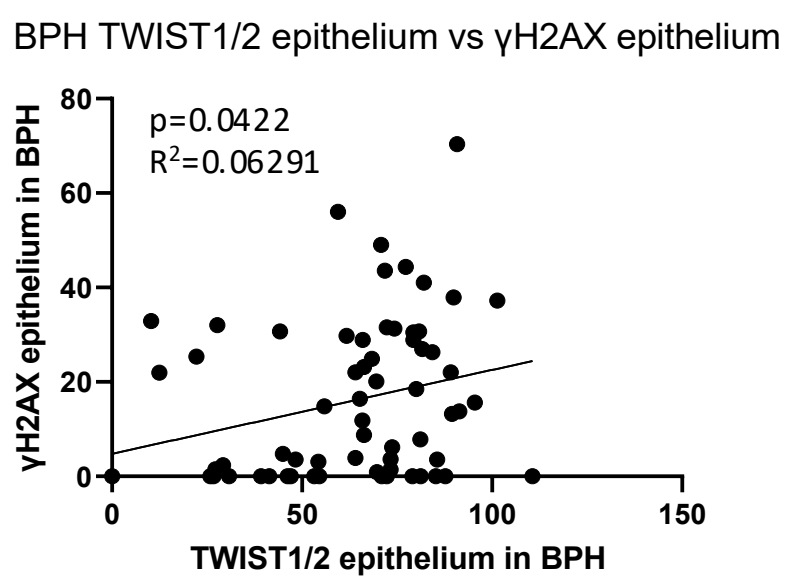

K

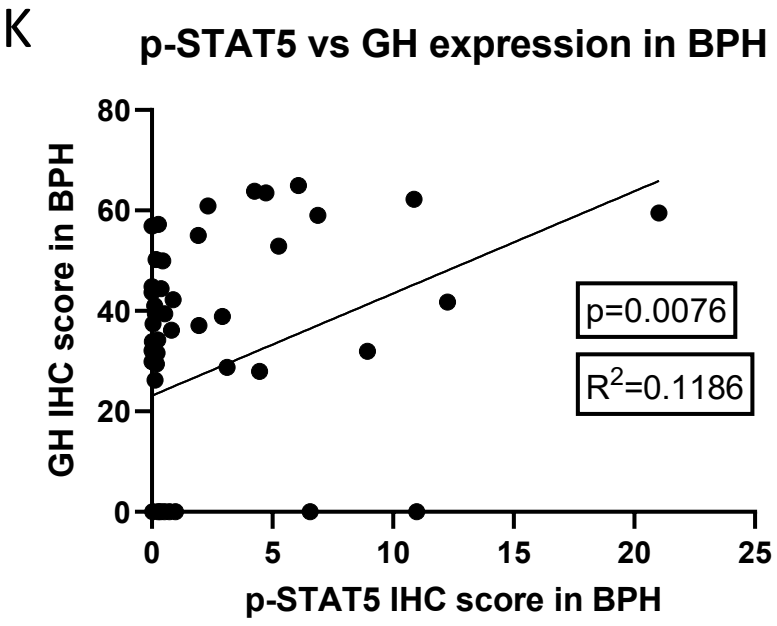

Supplemental Figure 7. Human prostate npGH, γH2AX, and TWIST1/2 are induced in aged normal prostate tissue and in BPH. (A-B) GH expression in BPH or normal human prostate assessed by chi-squared test. Number of tissue specimens with positive or negative staining in **(A)** epithelium or **(B)** stroma in aged cohorts. **(C)** Correlation between epithelial and stromal GH in BPH analyzed by simple linear regression. **(D-E)** BPH or normal human prostate γH2AX expression assessed by chi-squared test. Number of tissue specimens with positive or negative **(D)** epithelial or **(E)** stromal cells in aged cohorts. **(F-G)** Correlations in BPH between **(F)** epithelial and stromal γH2AX and **(G)** epithelial GH and γH2AX analyzed by simple linear regression. **(H)** TWIST1/2 expression in BPH or in normal human prostate assessed by chi-squared test. Number of tissue specimens with positive or negative staining in aged cohorts. **(I-J)** Correlation between **(I)** BPH epithelial TWIST1/2 and GH or **(J)** epithelial TWIST1/2 and γH2AX analyzed by simple linear regression. In C, F, G, I, J each dot represents IHC staining intensity in individual patient. **(K)** Correlation between GH and pSTAT5 expression in BPH epithelial cells.

A

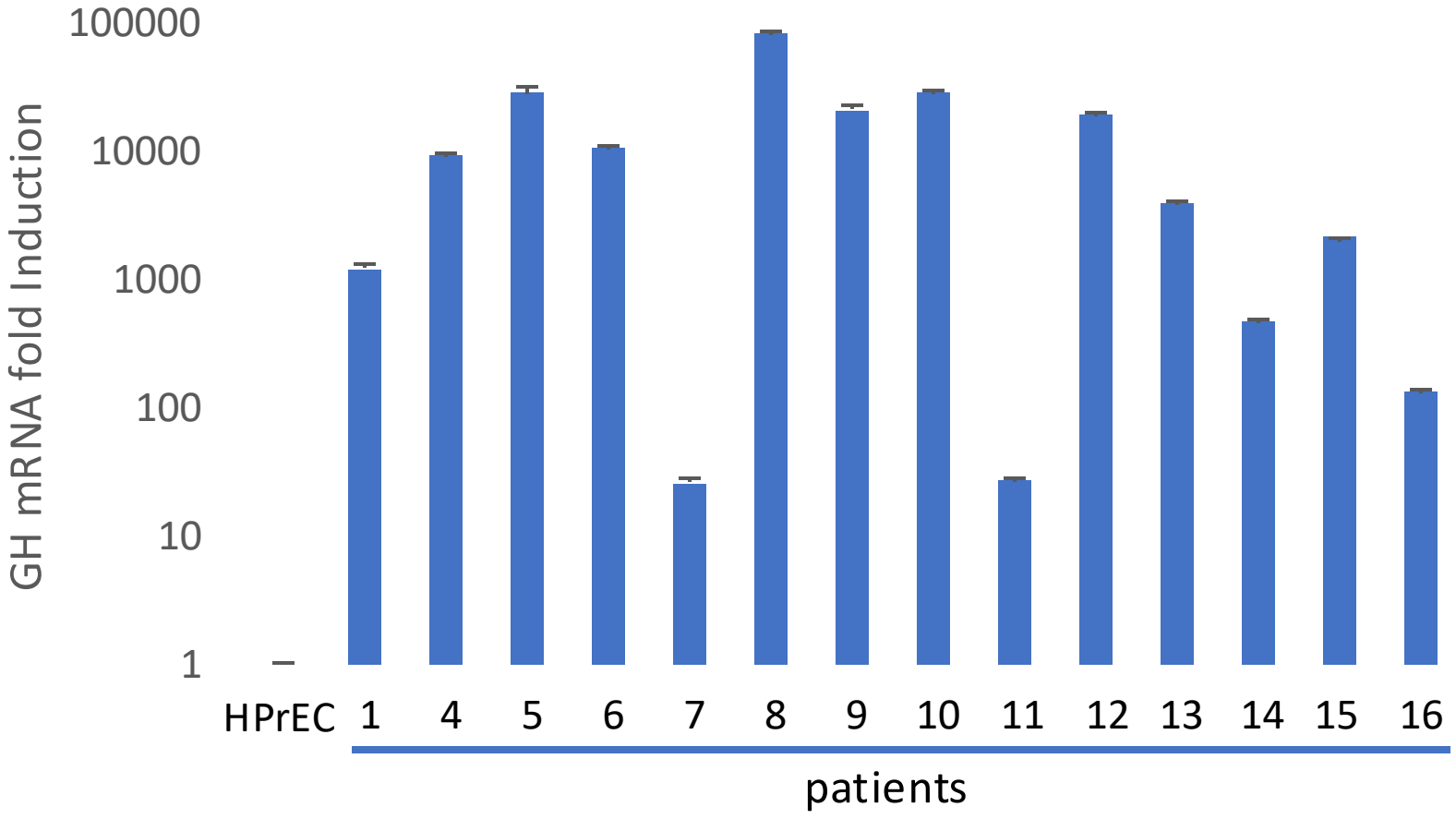

Supplemental Figure 8. npGH expression in human BPH specimens. (A) Real-time PCR of GH mRNA expression in HPrEC and BPH samples. Normalized PCR results are expressed in fold change from HPrEC taken as 1.

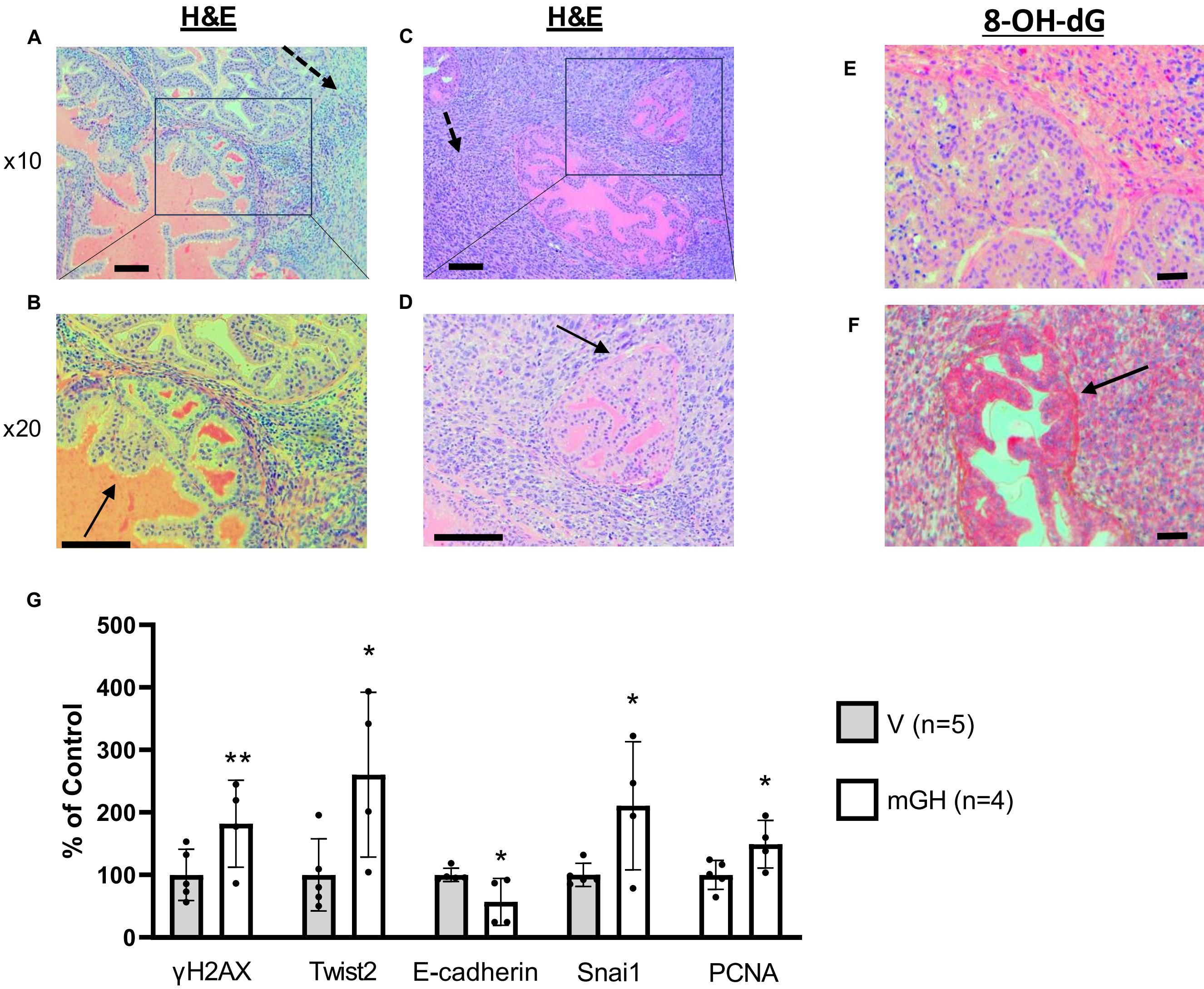

Supplemental Figure 9. Endocrine/paracrine GH induces changes consistent with BPH. Murine anterior prostate glands in 8-week-old C57BL/6 mice were injected anterodorsally with 6×10^5 mouse prostate fibroblasts infected with lentivirus expressing mGH (mGH) or empty vector (V) and sacrificed after 6 weeks. **(A-D)** Representative H&E staining images of prostate tissue from mice injected with lenti-mGH fibroblasts **(A, C)** imaged at x10 and **(B, D)** insets imaged at x20; allograft tumors are marked by dashed arrow and hyperplastic (BPH) areas are marked by solid arrows. **(E,F)** Representative images of 8-OH-dG staining of prostate tissue from mice injected with lentiV**(E)** or lenti-mGH **(F)** fibroblasts. Arrow marks intense staining in hyperplastic BPH area in mice injected with lenti-mGH fibroblasts. **(G)** ImageJ quantification of protein expression normalized to loading controls. Protein expression in dorsolateral prostate tissue derived from mice 8 weeks after injection of mouse prostate fibroblasts infected with lentivirus expressing mGH or V into both anterior prostate glands. Average measurements of 4 mice with mGH and 5 mice with V inoculants are shown. Each dot represents sample analysis derived from an individual mouse. Results are shown as mean ± SEM for each group. Data are graphed as percent of control, but statistical testing was performed on raw numbers. Differences were assessed with 2-tailed t test. $^*p<0.05$, $^{**}p<0.01$ versus control.
