# Supplementary material for: Local growth hormone promotes benign prostatic hyperplasia
Source: JCI Insight. 2026 Jul 28;11(17):e206012. doi: 10.1172/jci.insight.206012 (PMC13564083; doi:10.1172/jci.insight.206012)
Supplement: Supplementary file 2 [file jciinsight-11-206012-s161.pdf]

# Full unedited gels for Figure 1F

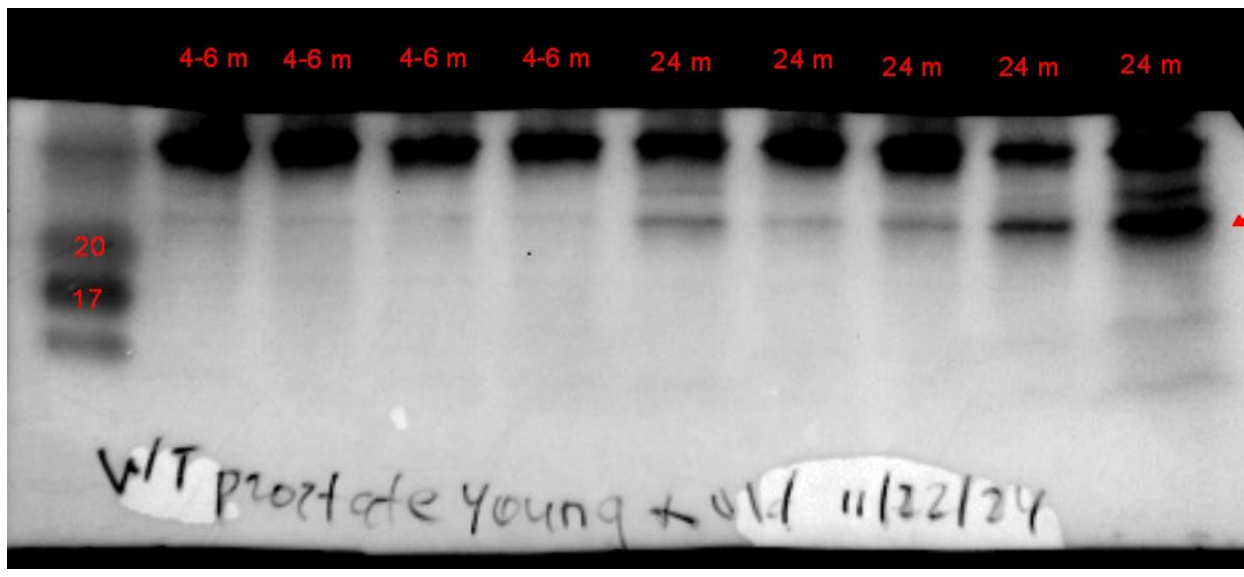


GH

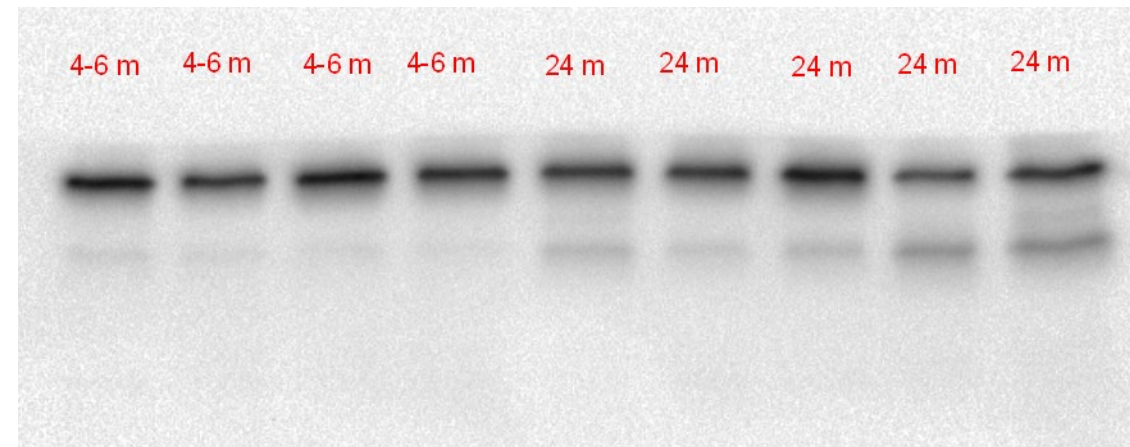


GAPDH

# Full unedited gels for Figure 2A-B

## Figure 2A

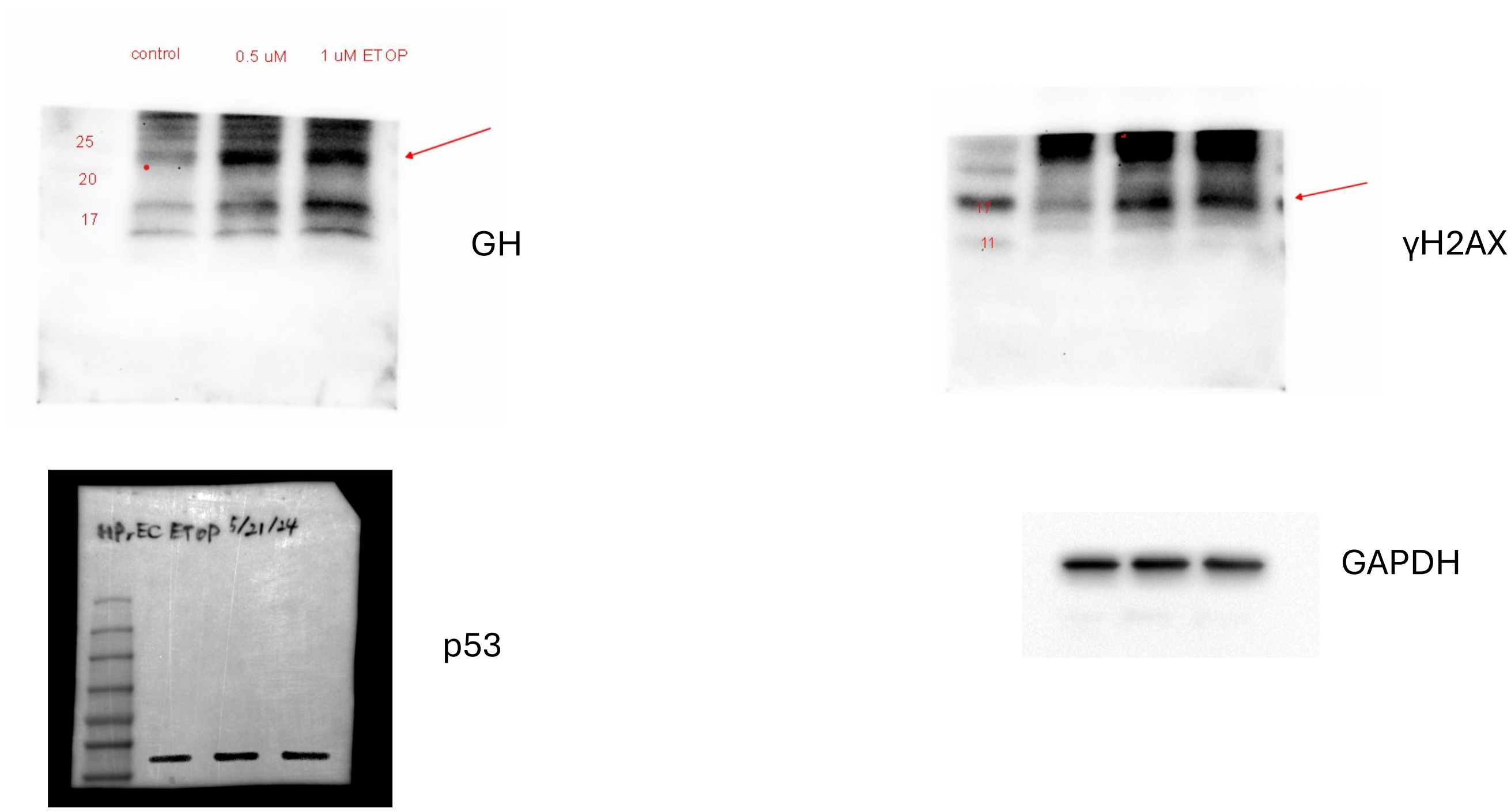


## Figure 2B

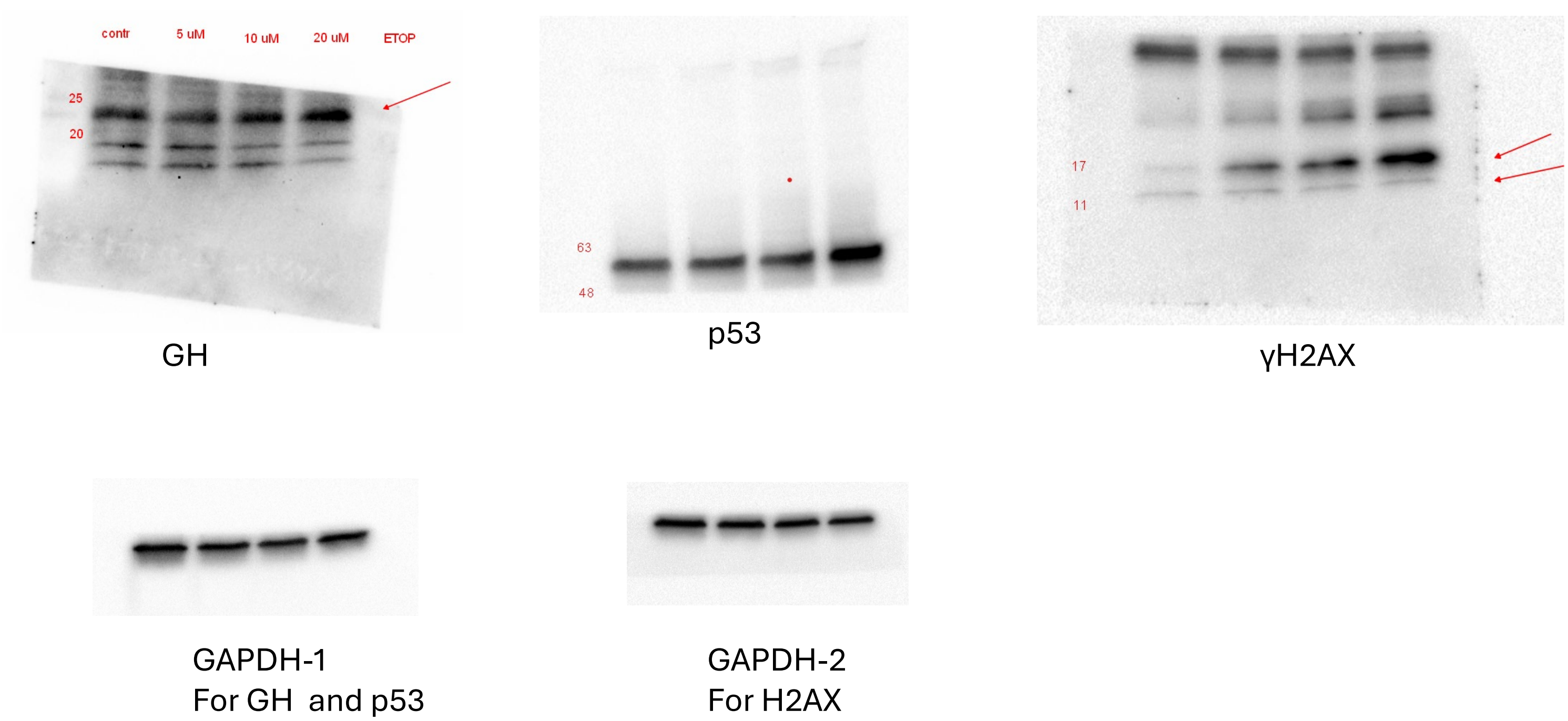

# Full unedited gels for Figure 2C-E

## Figure 2C

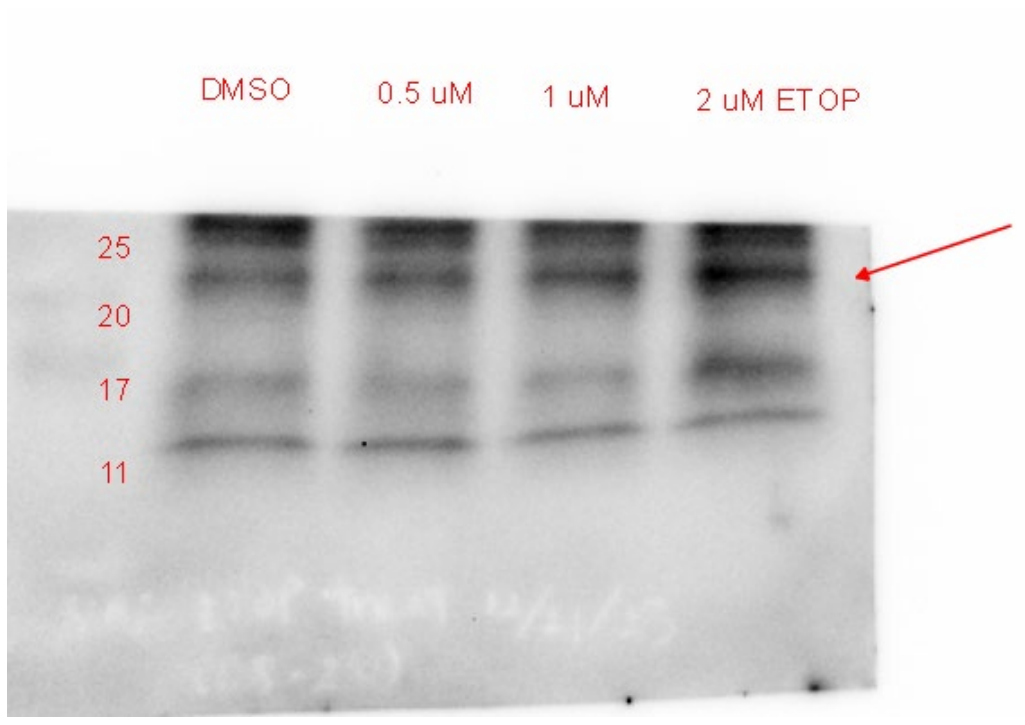


GH

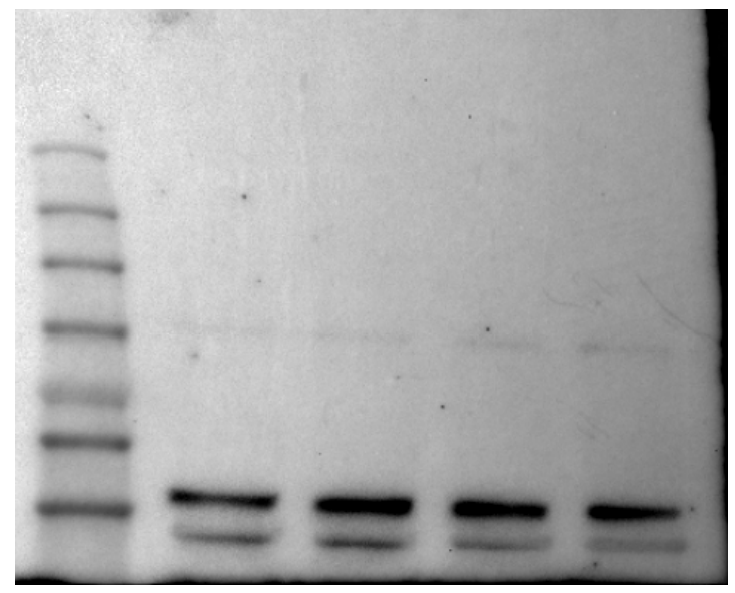

p53

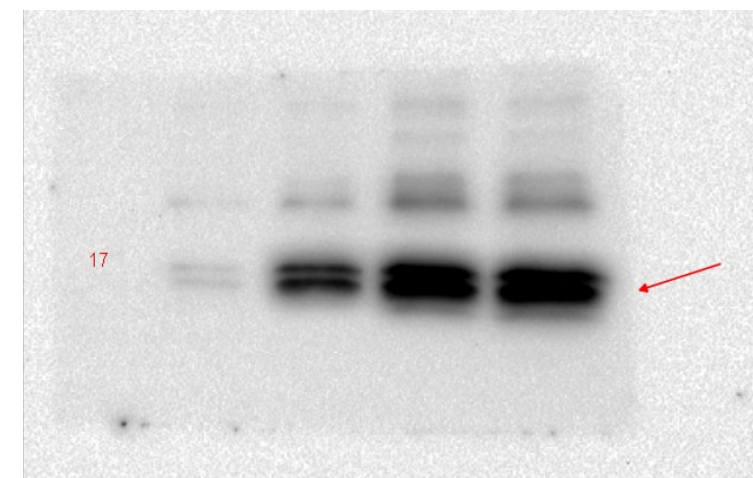


γH2AX

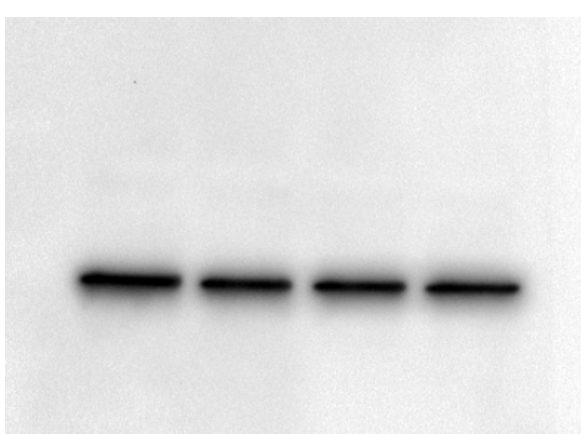

GAPDH-1for GH and p53

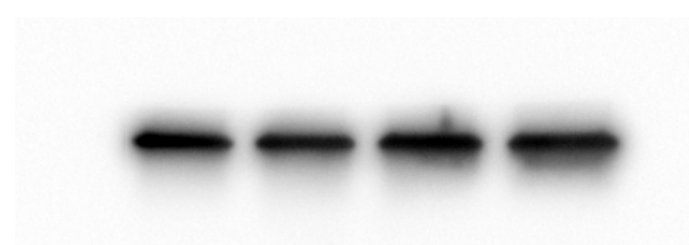

GAPDH-2for H2AX

---

## Figure 2D

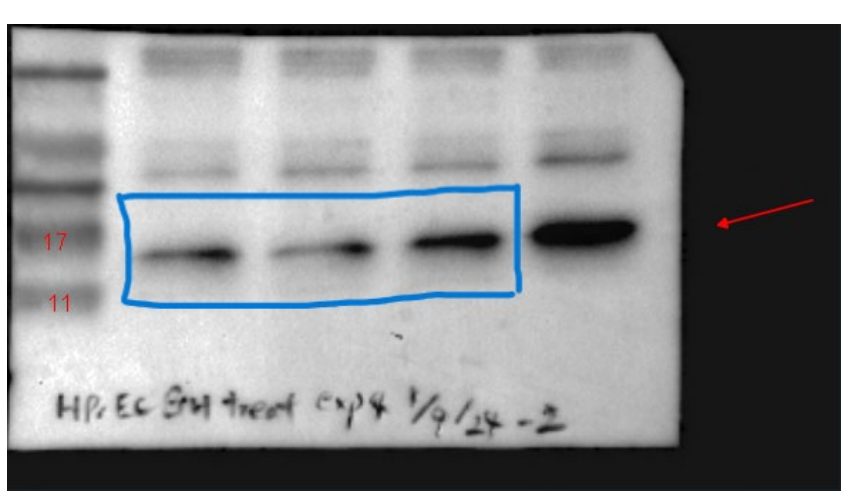


γH2AX

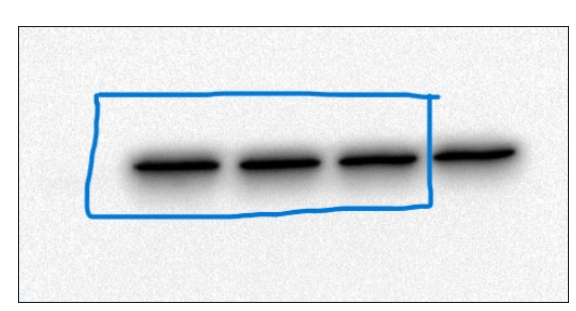

GAPDH

---

## Figure 2E

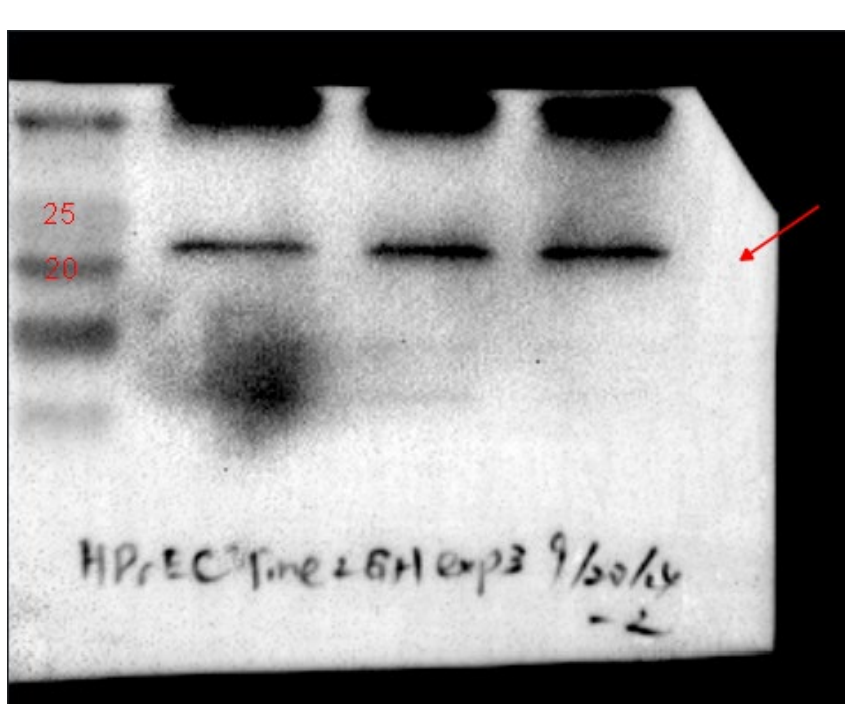


γH2AX

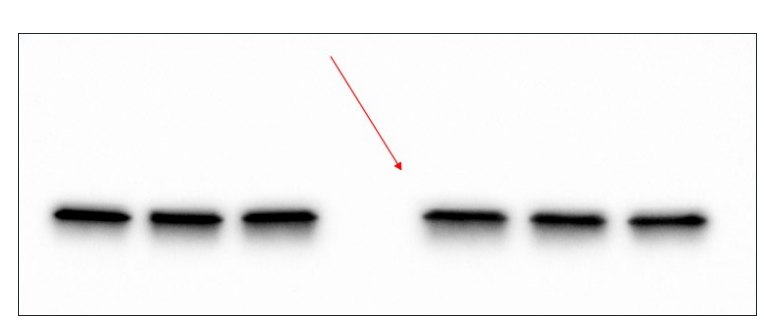

GAPDH

# Full unedited gels for Figure 2F-H

## Figure 2F

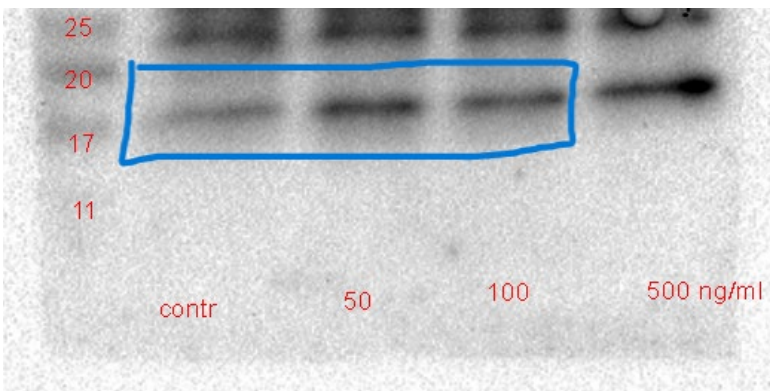


γH2AX

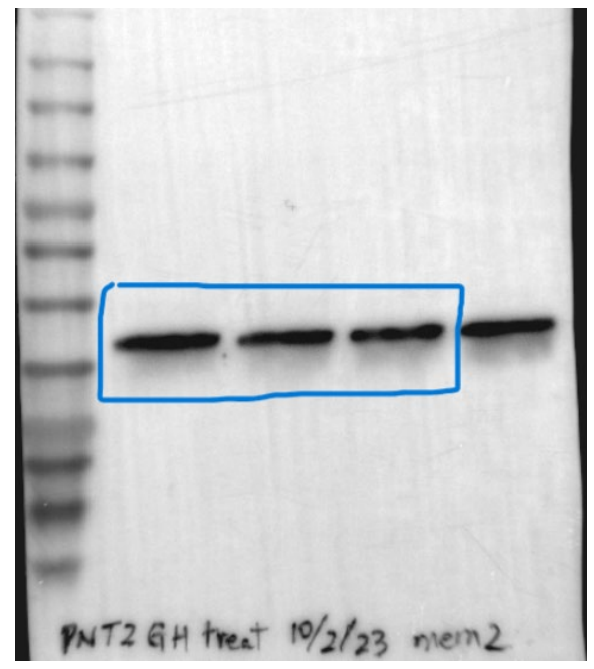


GAPDH

## Figure 2G

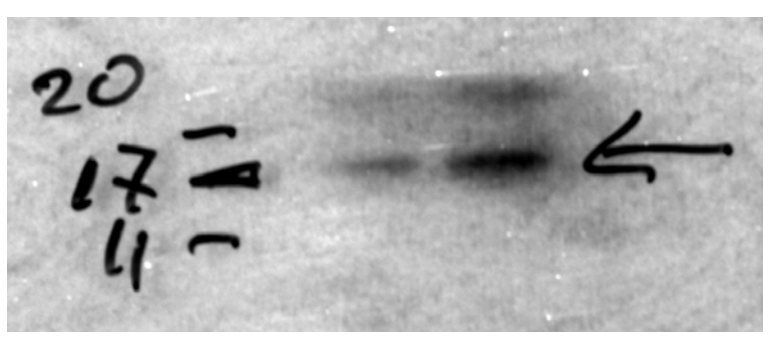


γH2AX

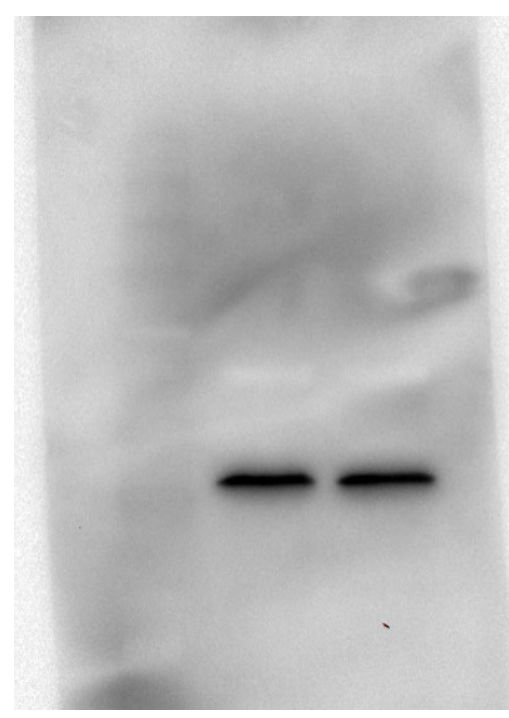

GAPDH

## Figure 2H

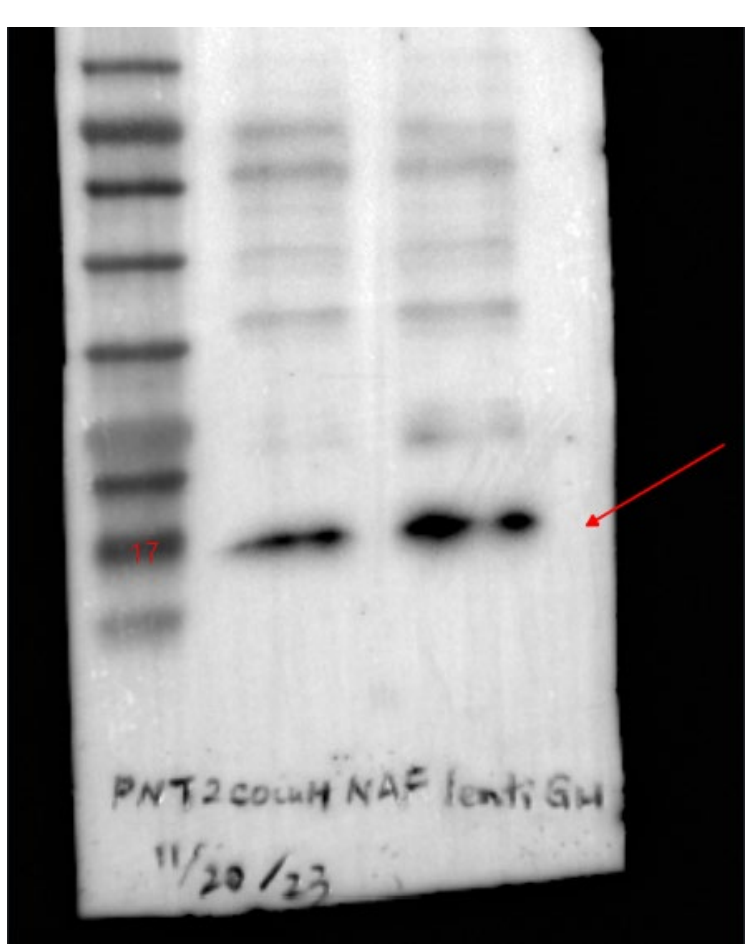


γH2AX

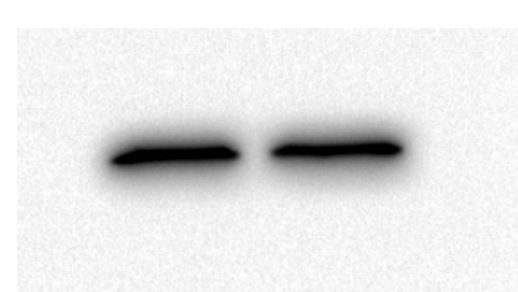

GAPDH

# Full unedited gels for Figure 2I, 2J

## Figure 2I

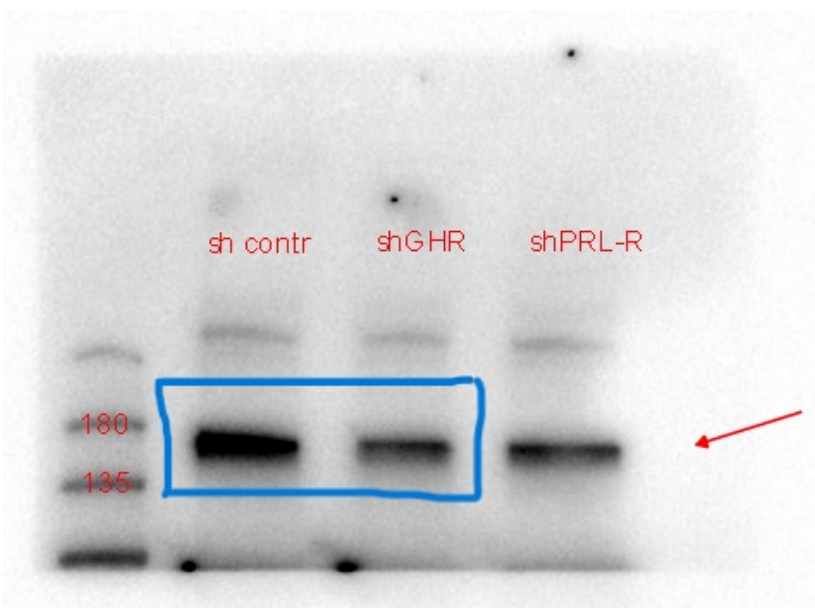


GHR

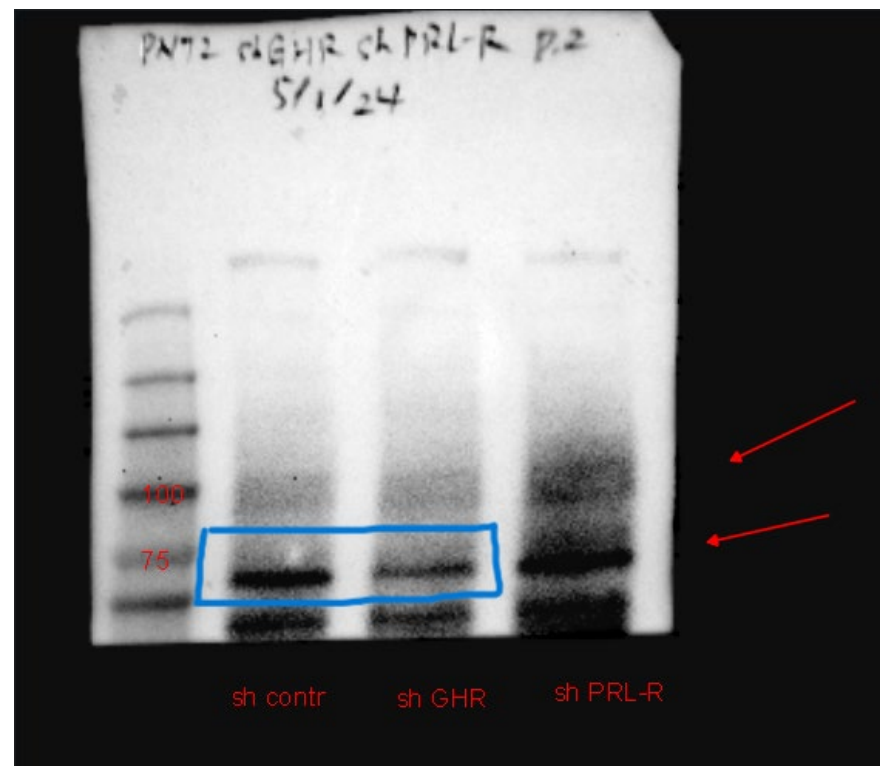


p-STAT5

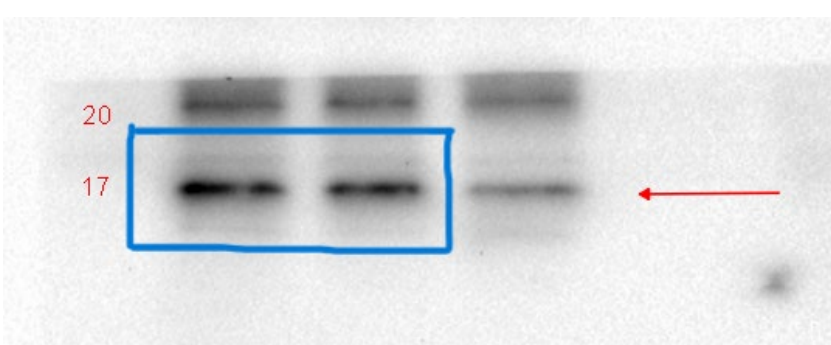


γH2AX

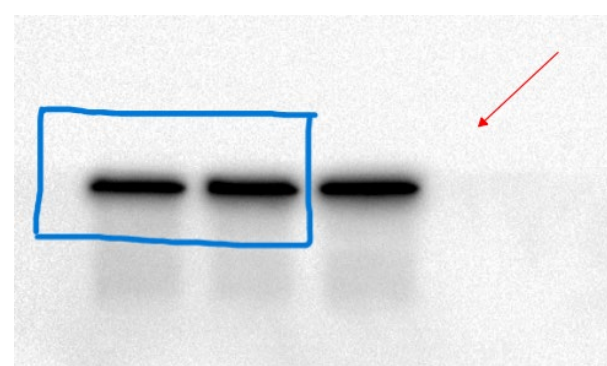

GAPDH-1for GHR and H2AX

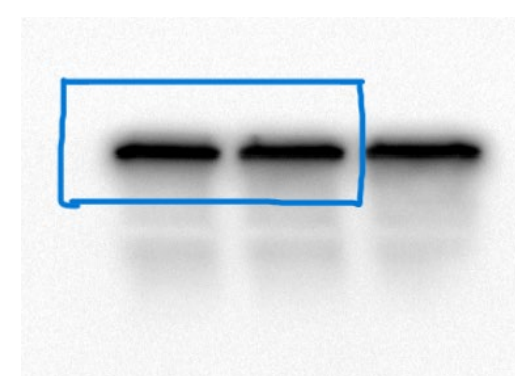

GAPDH-2 for pSTAT5

---

## Figure 2J

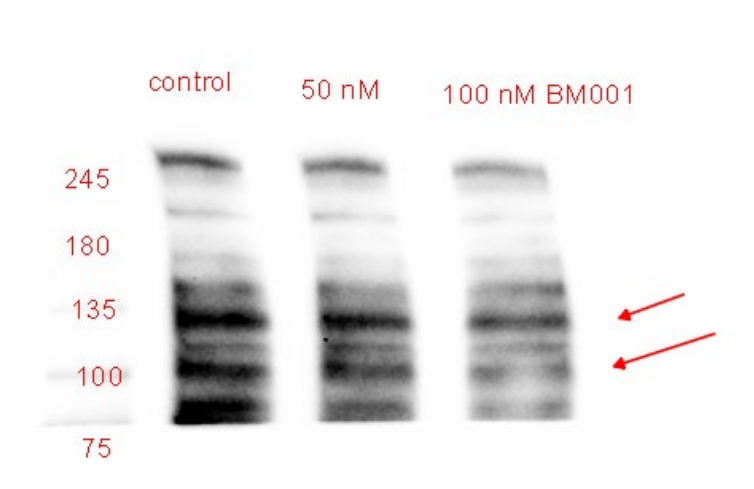


GHR

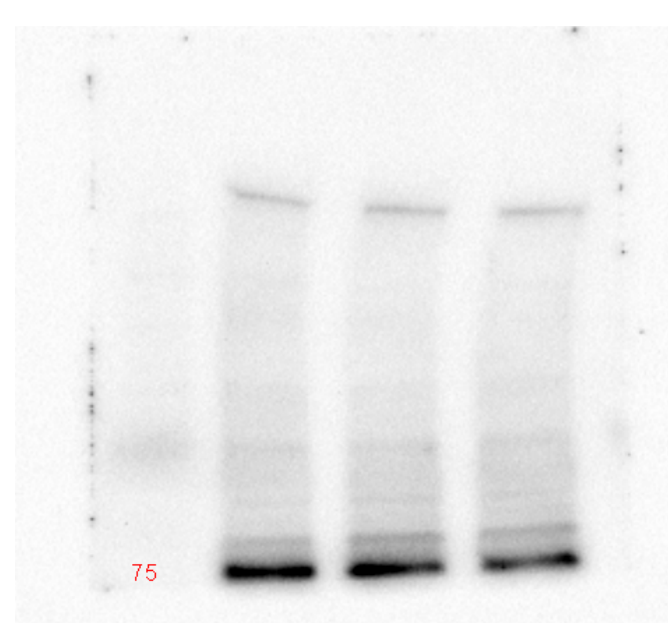


p-STAT5

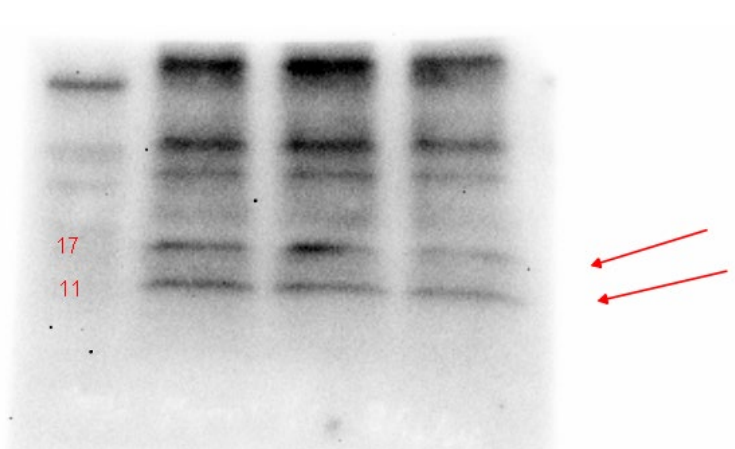


γH2AX

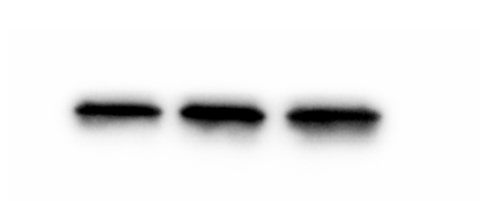

GAPDH-1

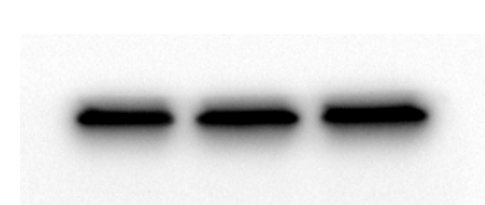

GAPDH-2

# Full unedited gels for Figure 2K, 2Q

## Figure 2K

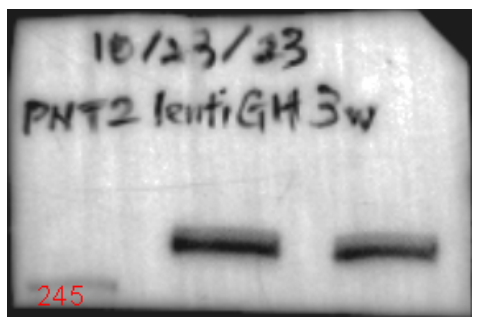


pATM

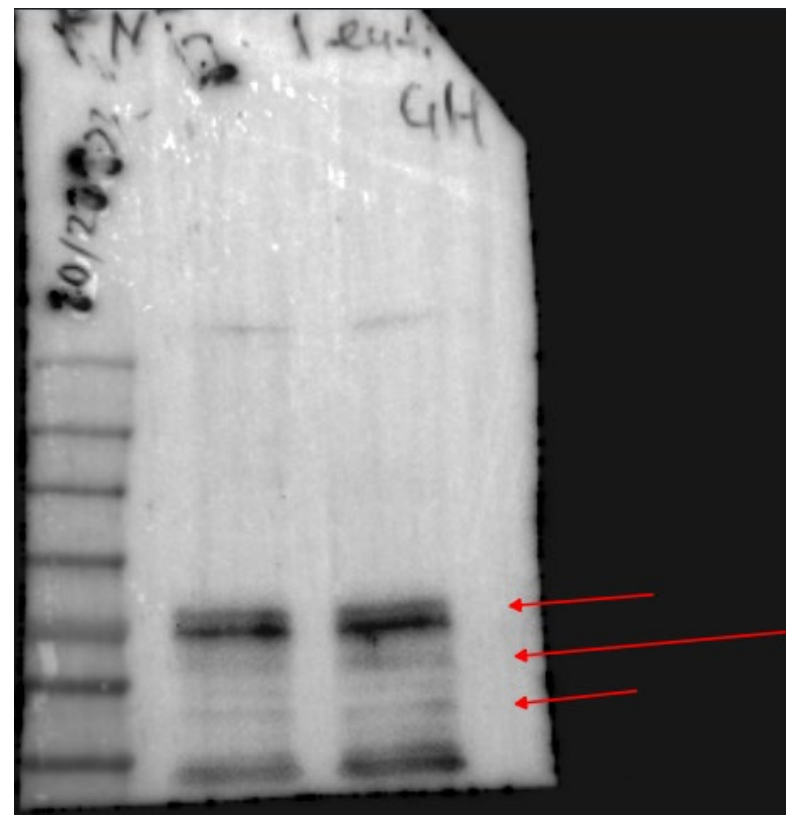


Wip1

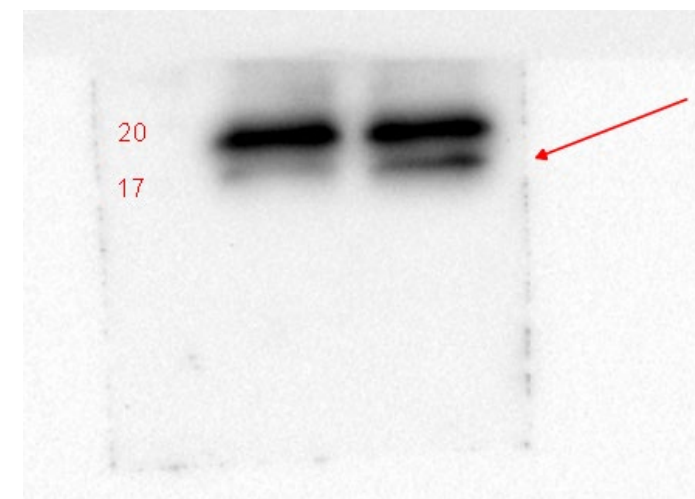


γH2AX

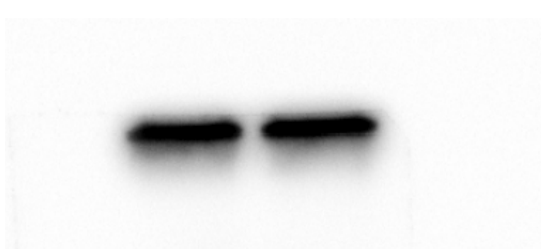

GAPDH-1for ATM and H2AX

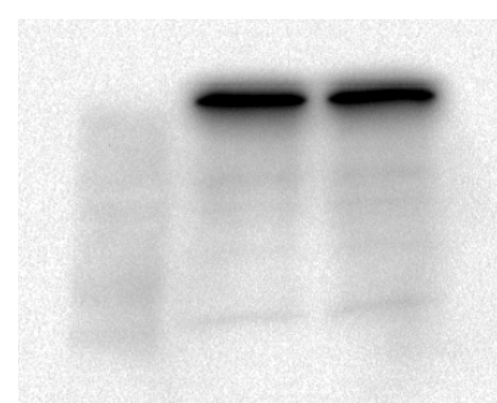

GAPDH-2 for Wip1

---

## Figure 2Q

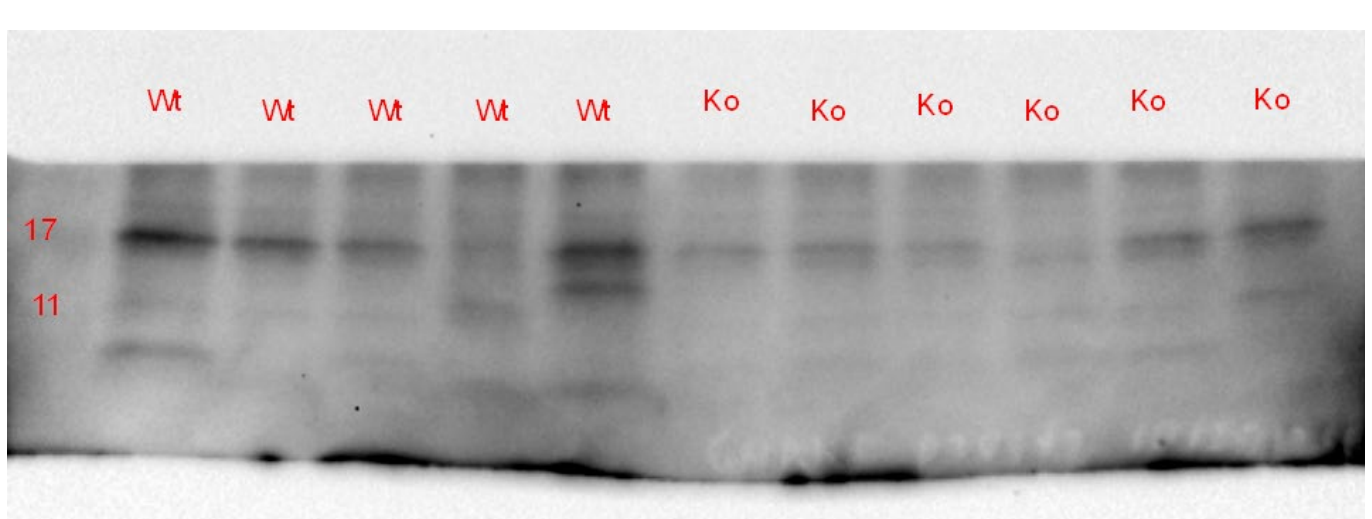


γH2AX

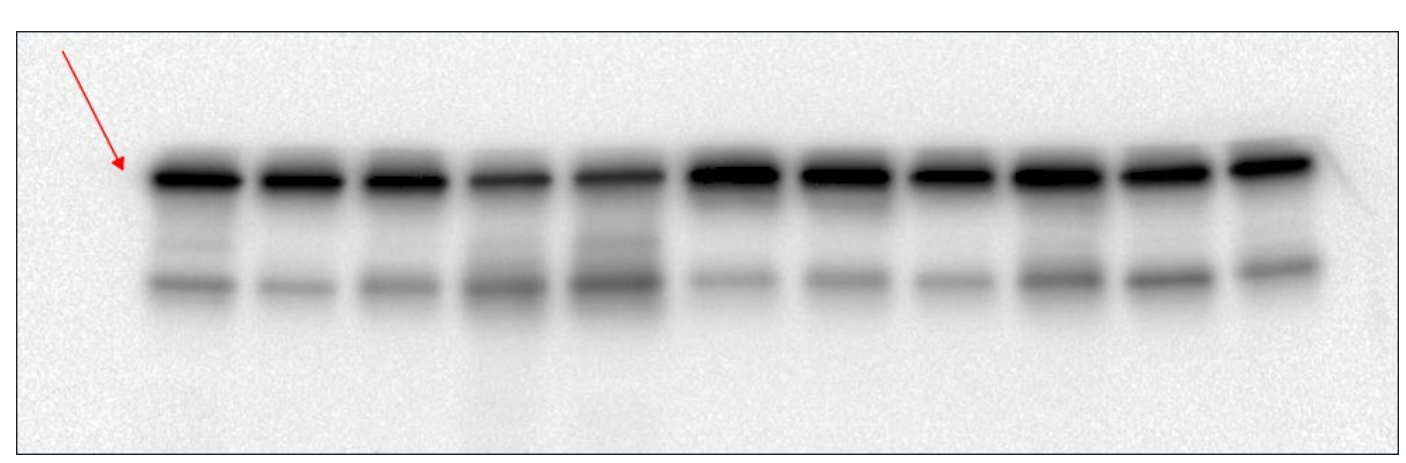

GAPDH

# Full unedited gels for Figure 3H

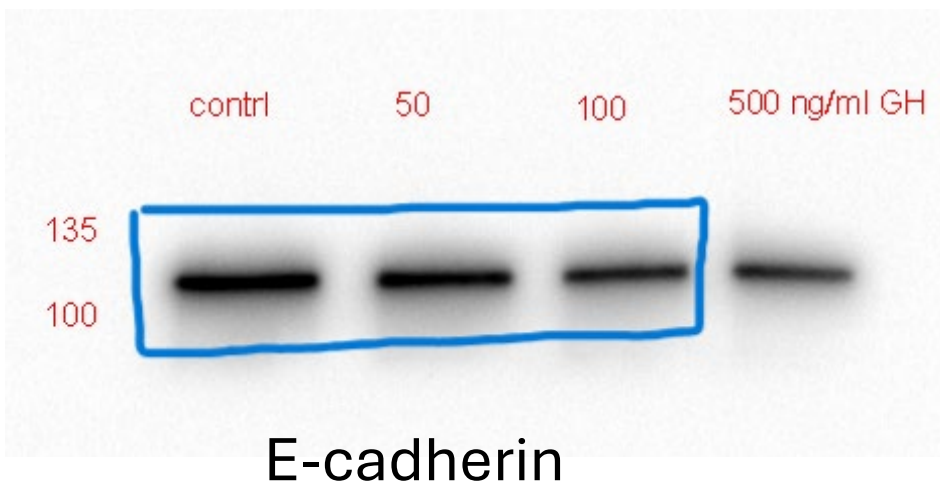


E-cadherin

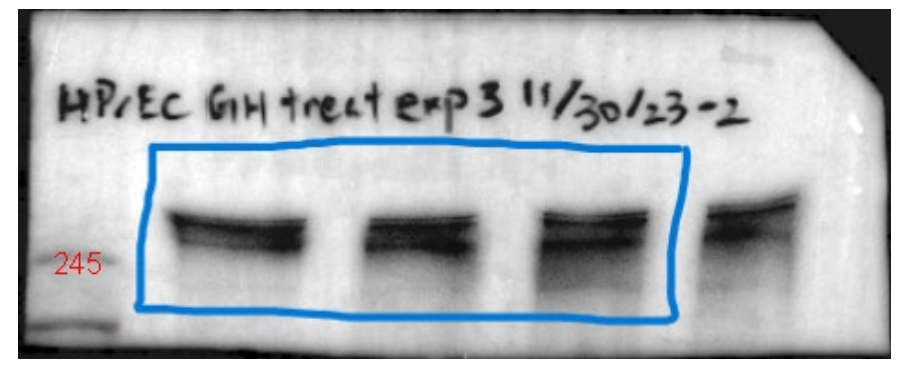


Ki-67

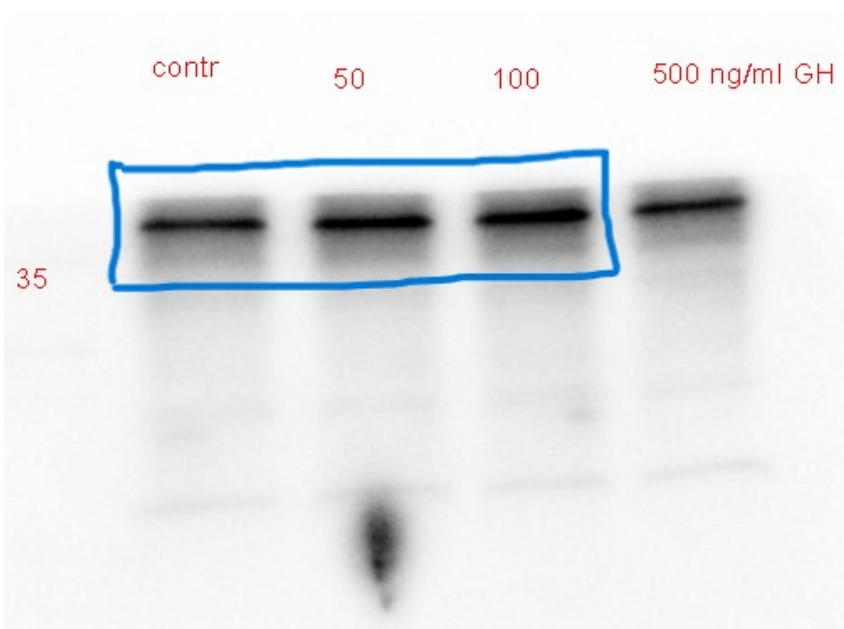


SNAI1

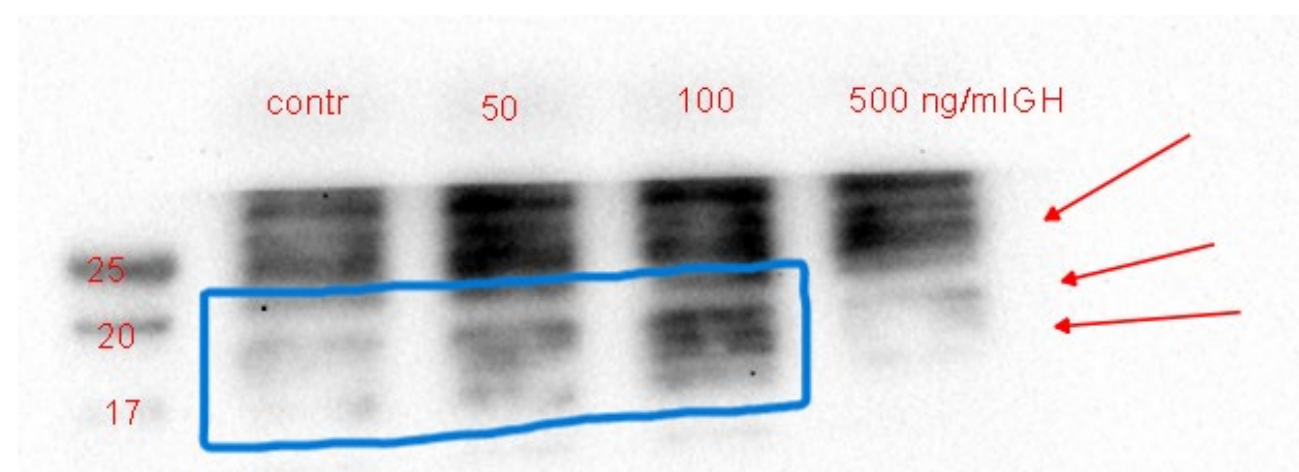


TWIST2

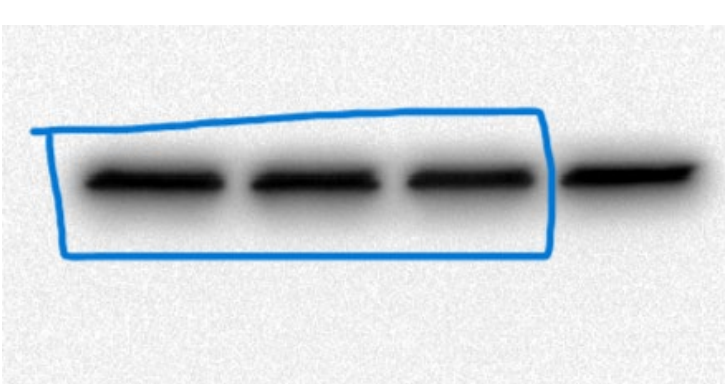

GAPDH-1 for Ki67 and Twist

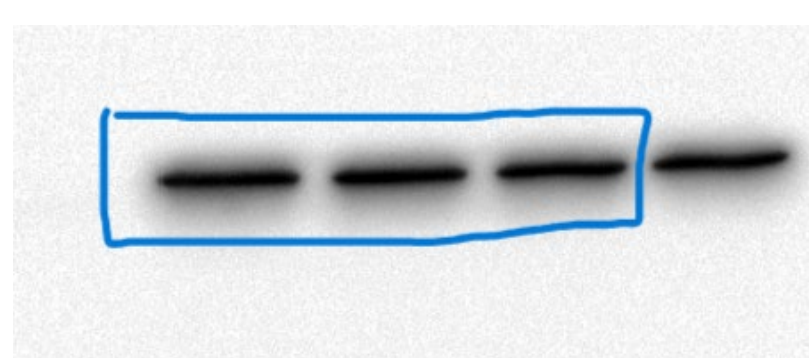

GAPDH-2 for E cadherin

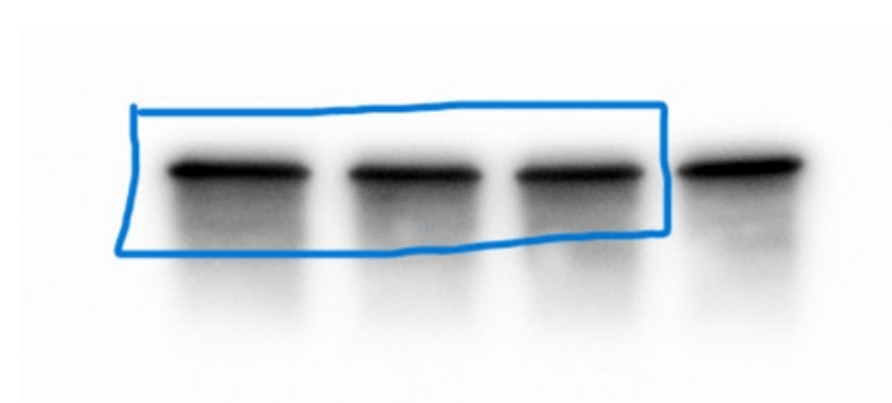

GAPDH-3 for Snai1

# Full unedited gels for Figure 3I

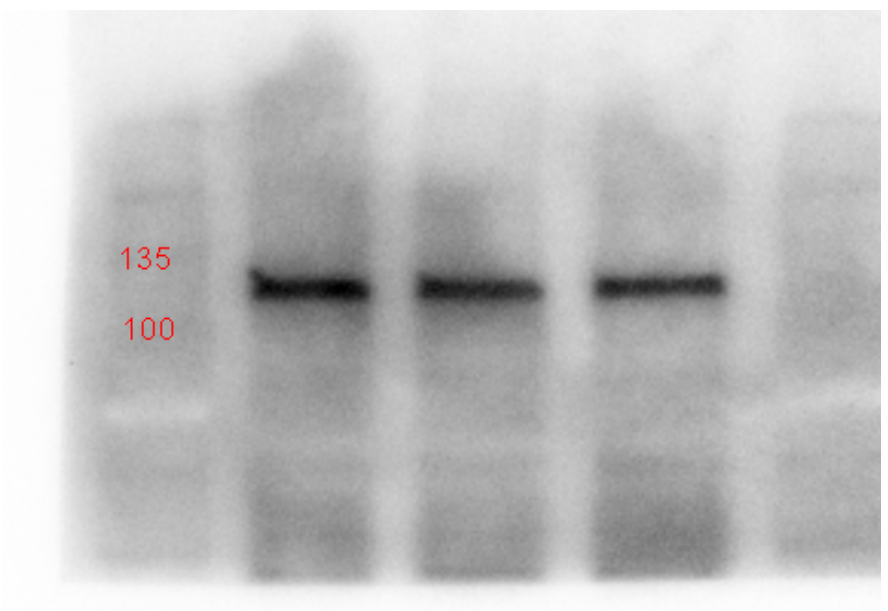


E-cadherin

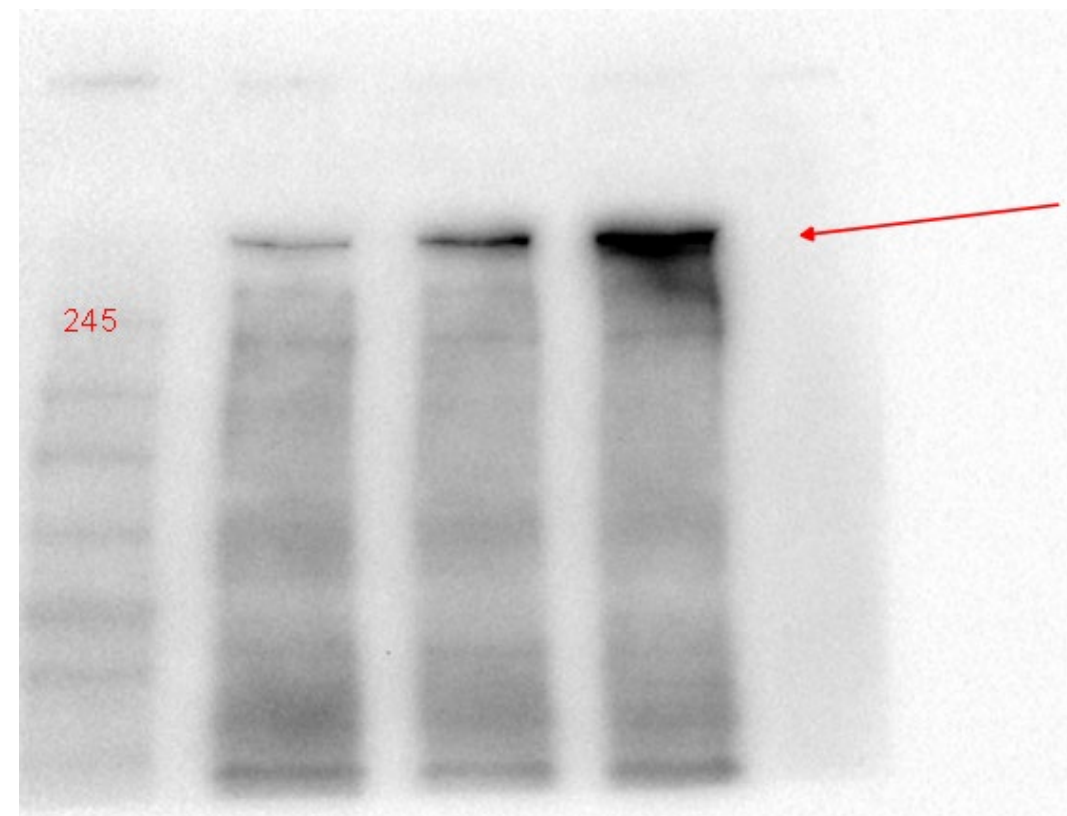


Ki-67

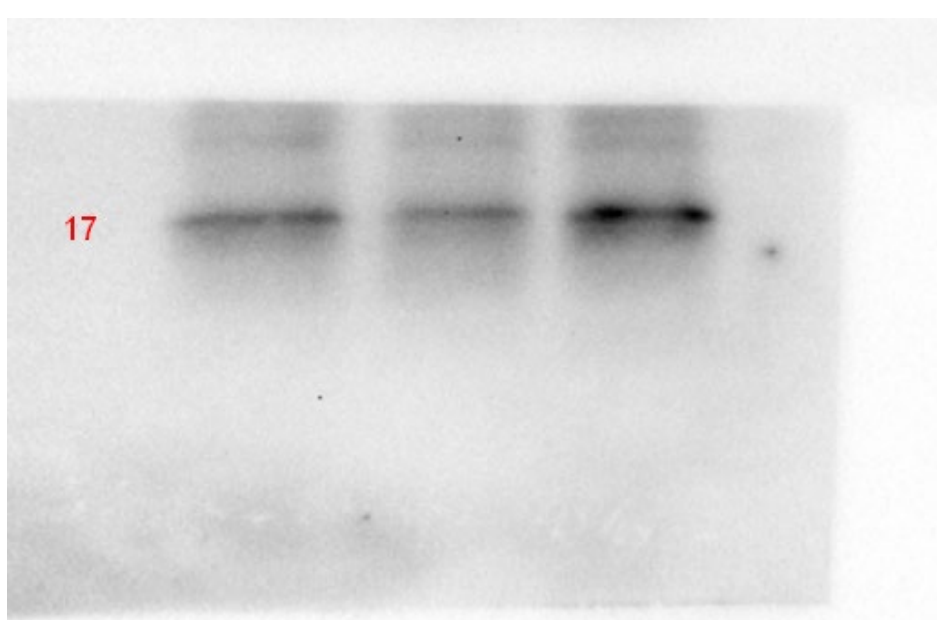


TWIST2

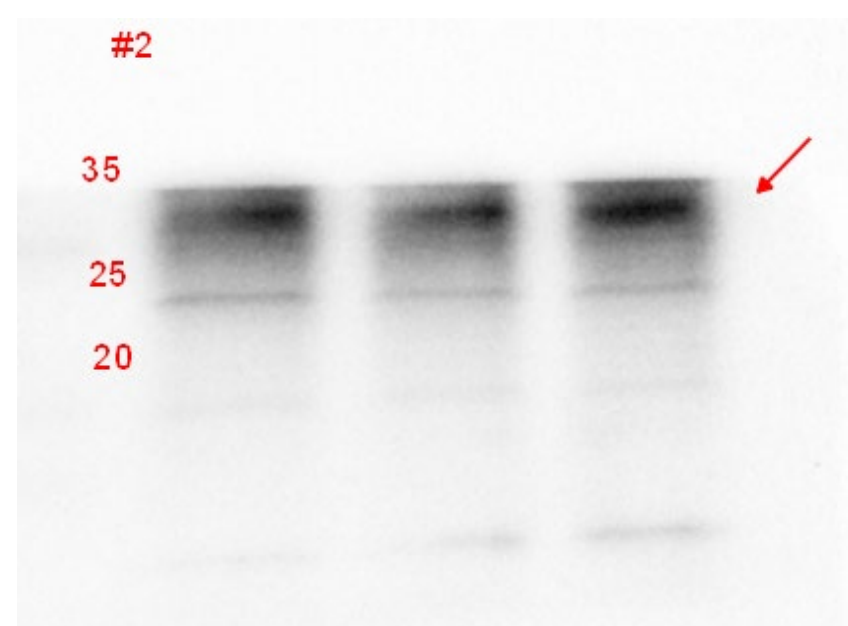


SNAI1

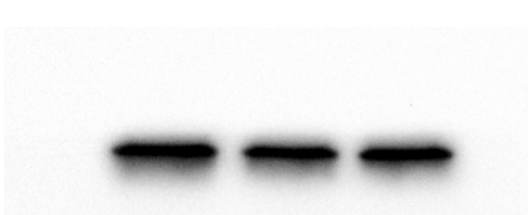

GAPDH-1for Twist2 and Ki67

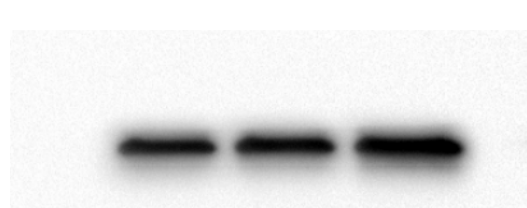

GAPDH-2for E cadherin

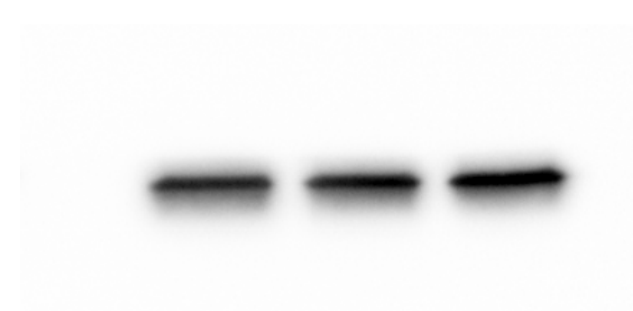

GAPDH-3for Snai1

# Full unedited gels for Figure 3J

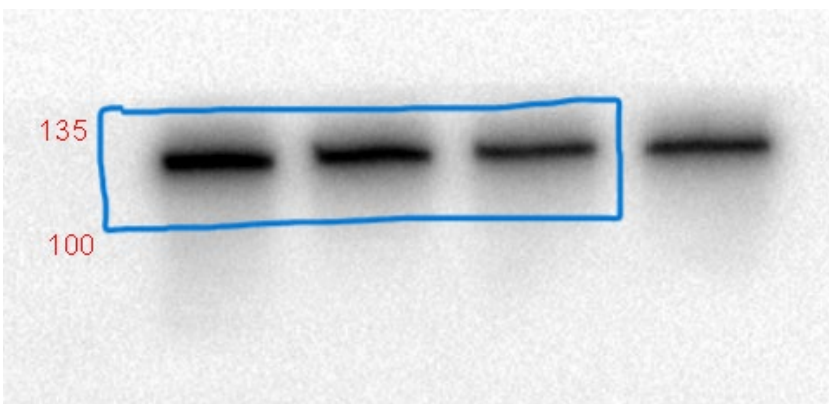


E-cadherin

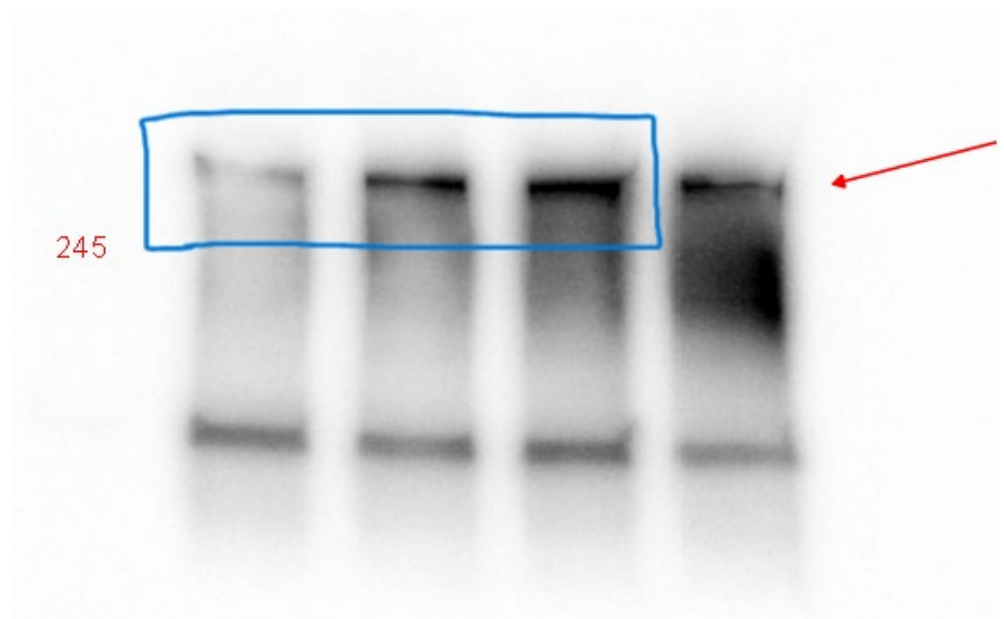


Ki-67

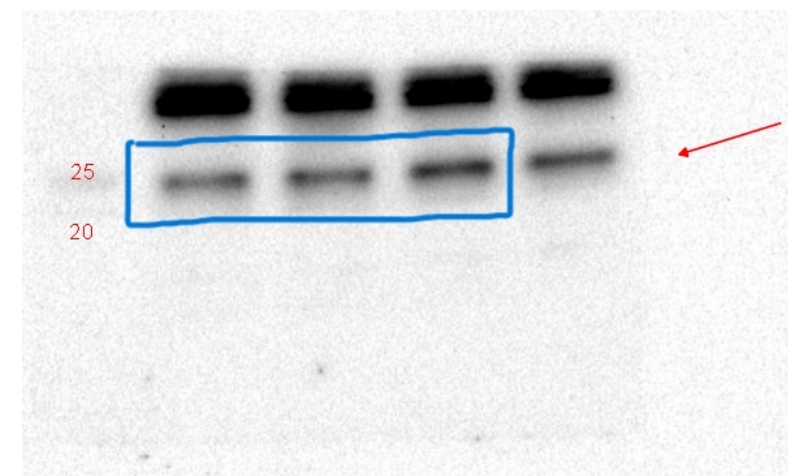


TWIST2

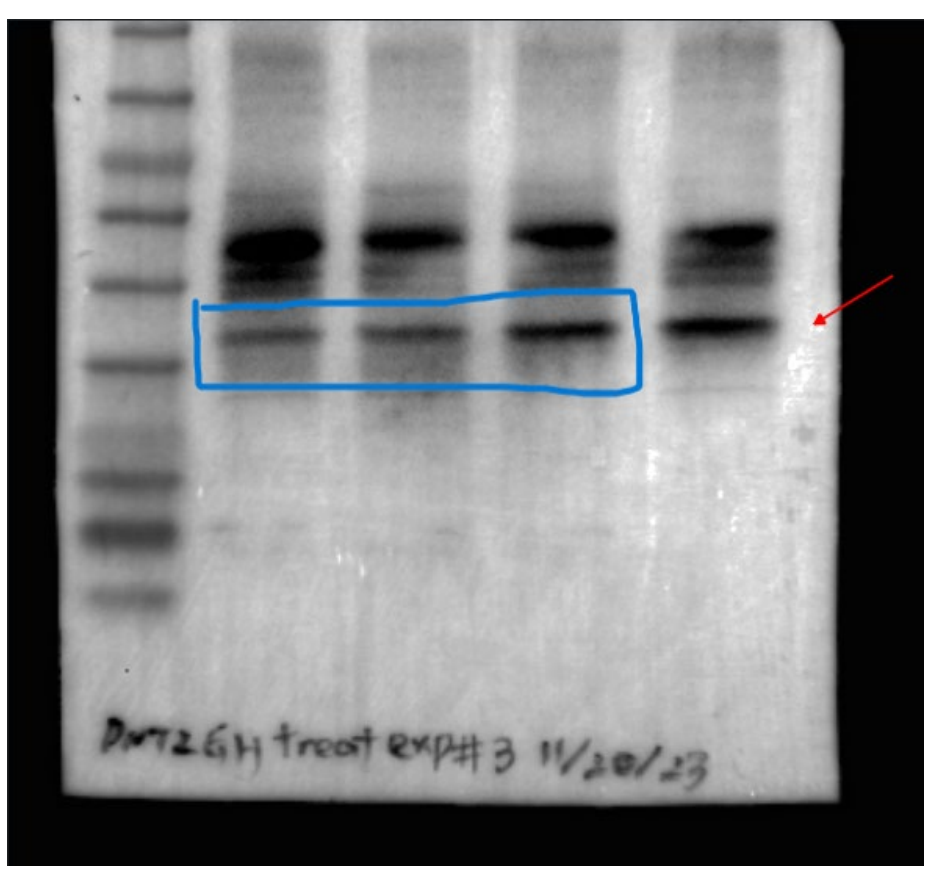

SNAI1

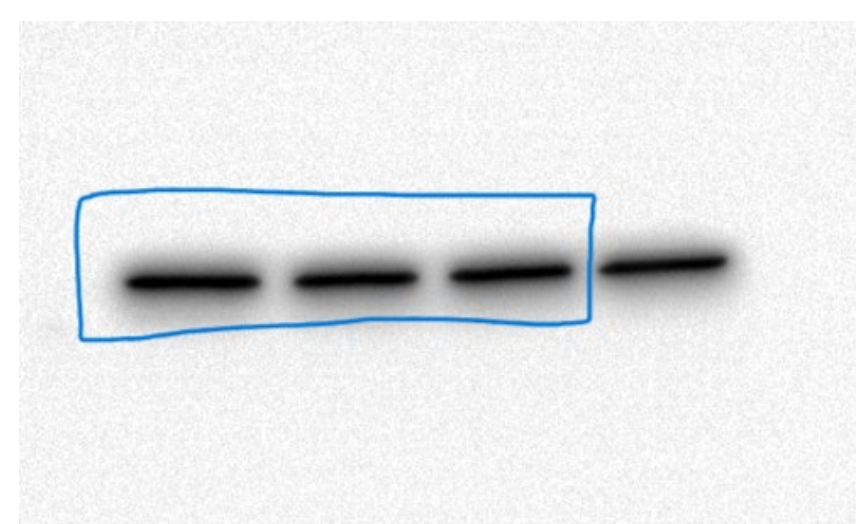

GAPDH-1 for Twist2

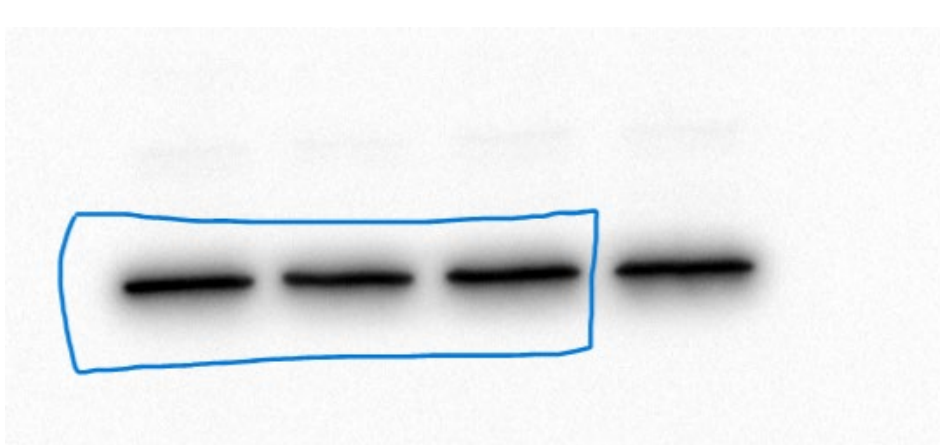

GAPDH-2for E cadherin and Twist2

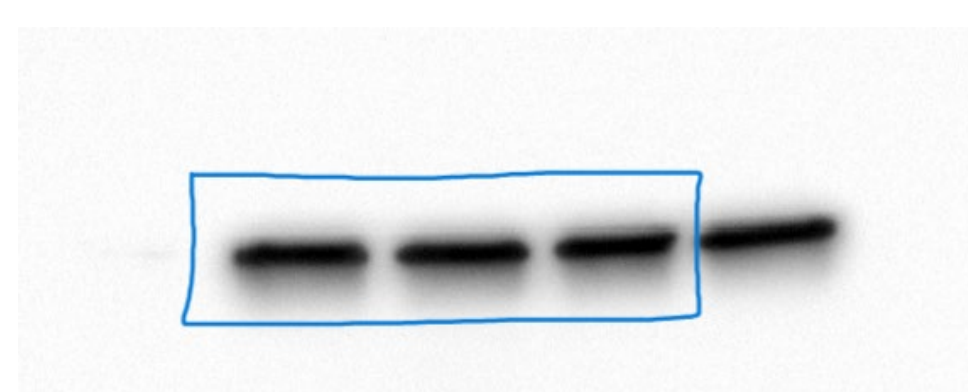

GAPDH-3for Ki67

# Full unedited gels for Figure 4A

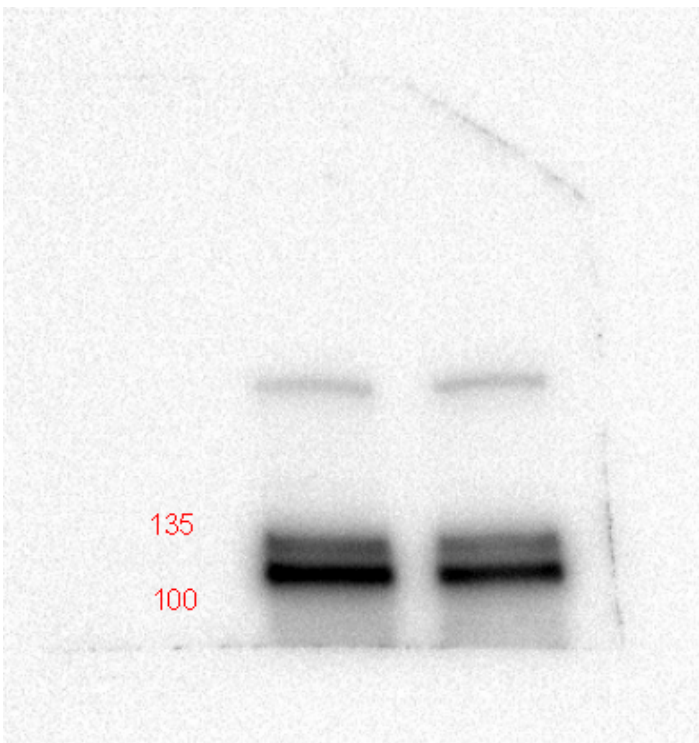


E-cadherin

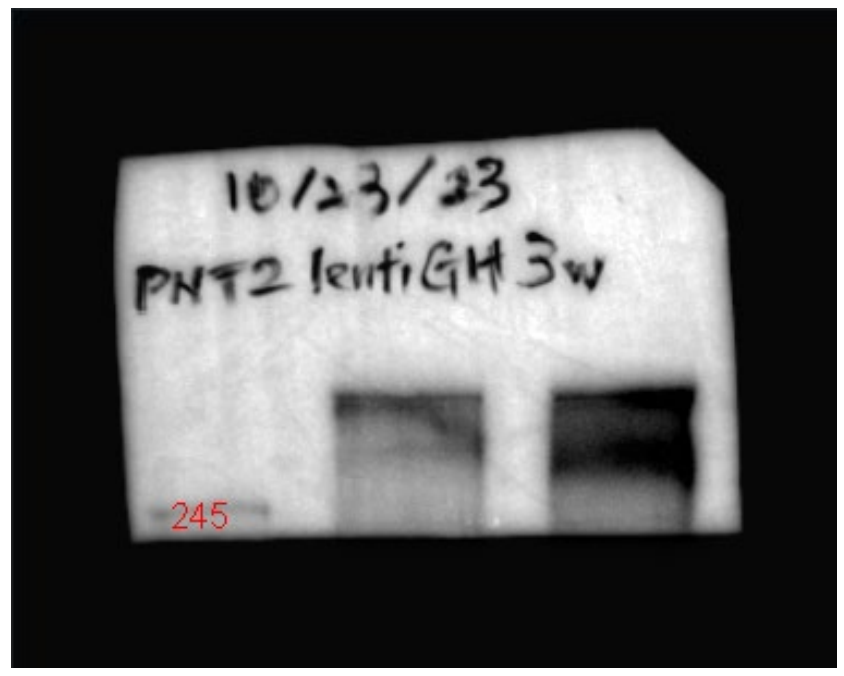


Ki-67

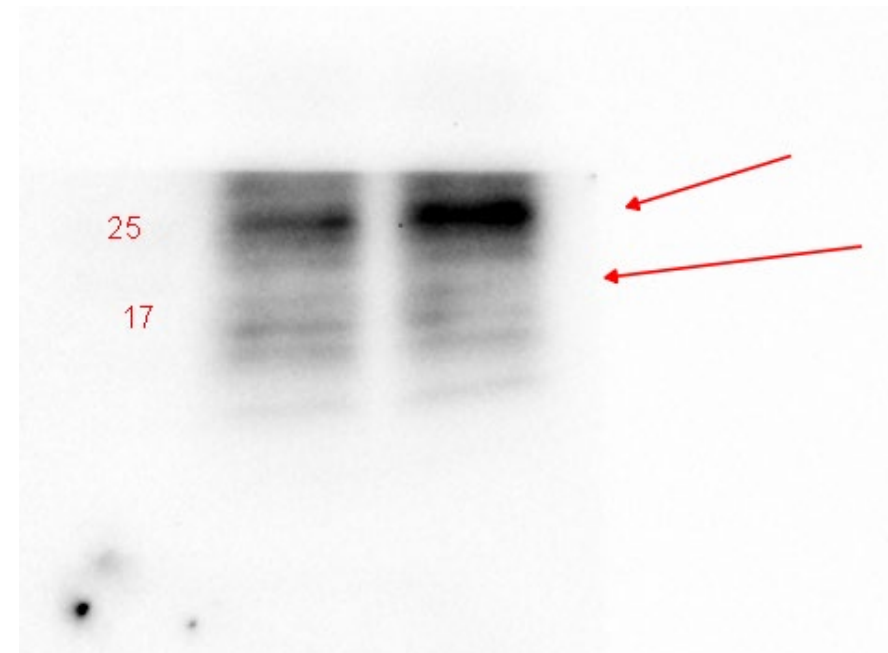


TWIST2

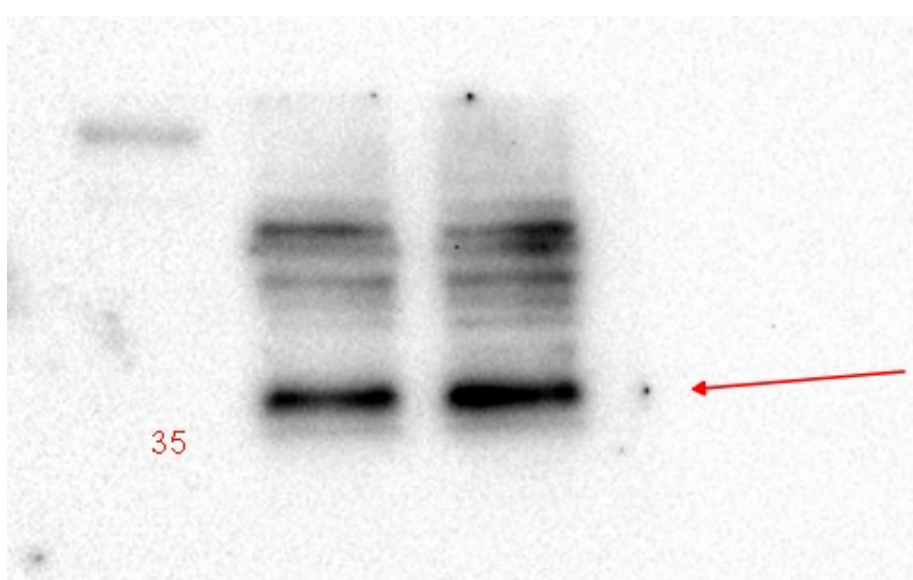


SNAI1

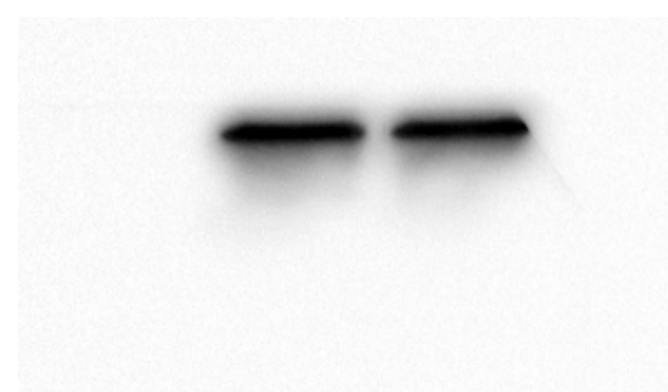

GAPDH-1 for Twist2

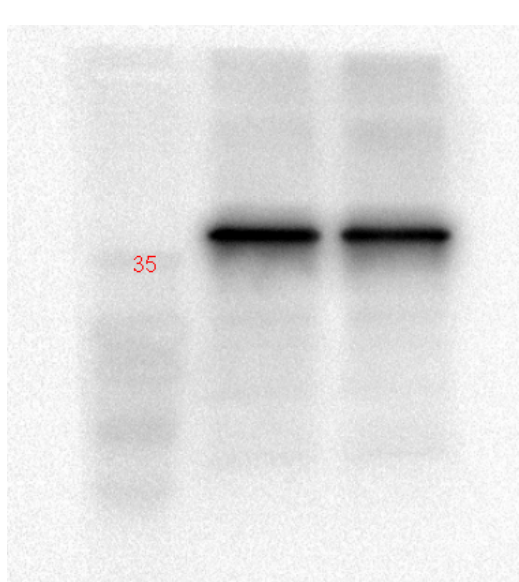


GAPDH-2 for Snai1

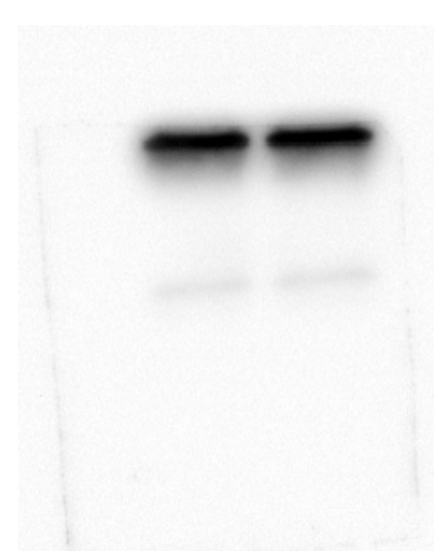

GAPDH-3for Ki67

# Full unedited gels for Figure 4B

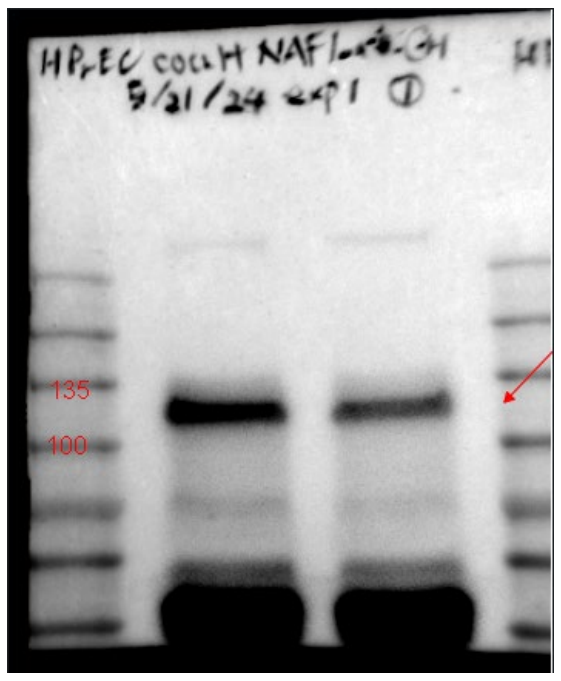


E-cadherin

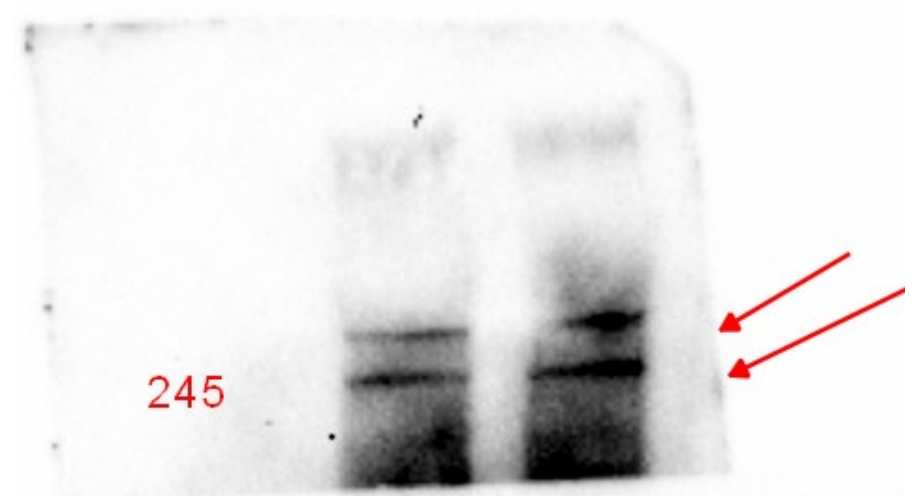


Ki-67

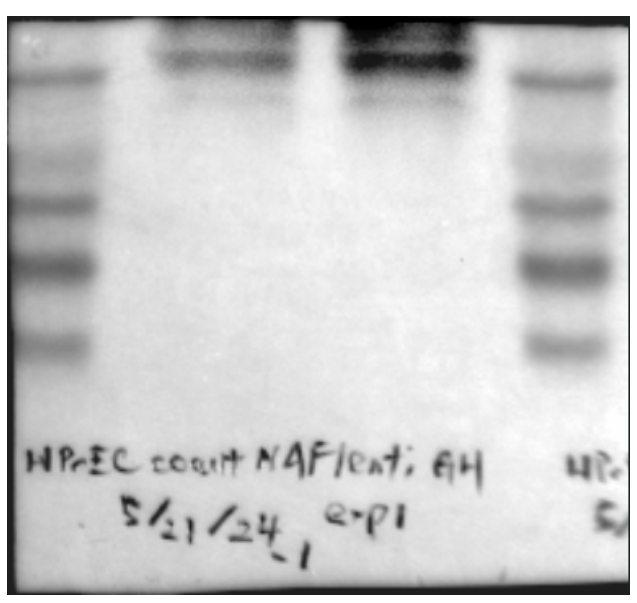

SNAI1

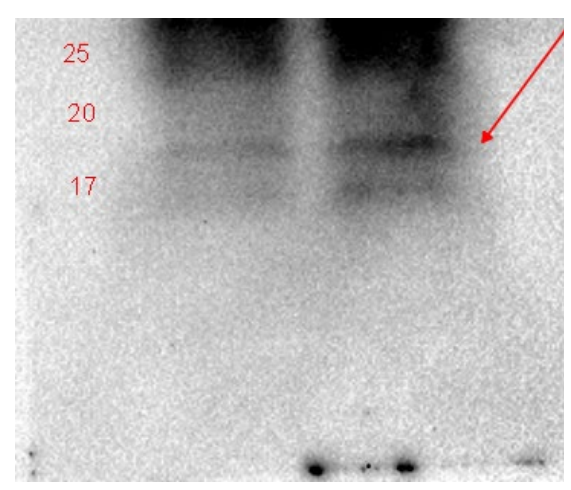


TWIST2

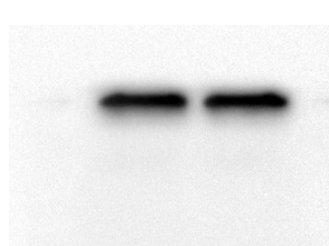

GAPDH-1fro twist2, E cadherin and Snai1

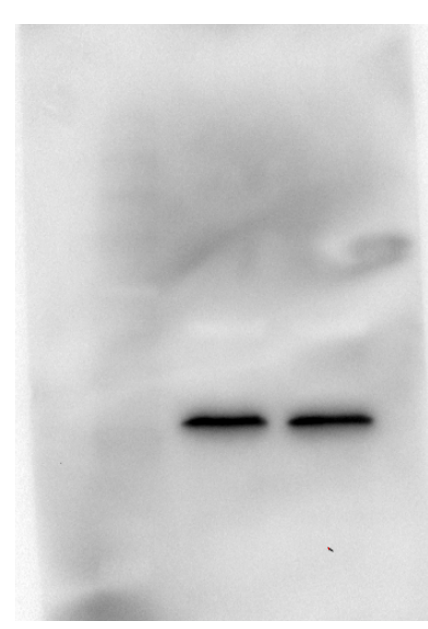

GAPDH-2 for Ki67

# Full unedited gels for Figure 4C

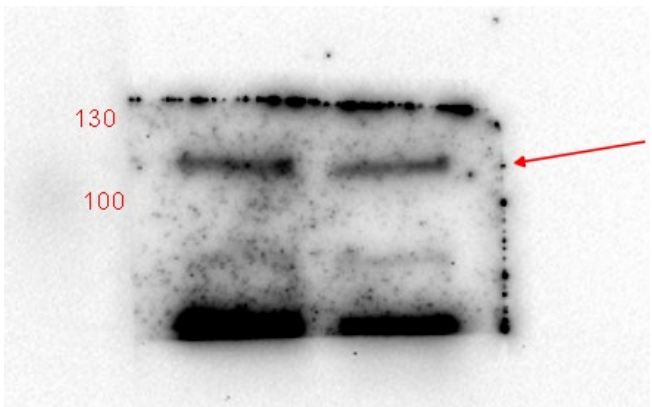


E-cadherin

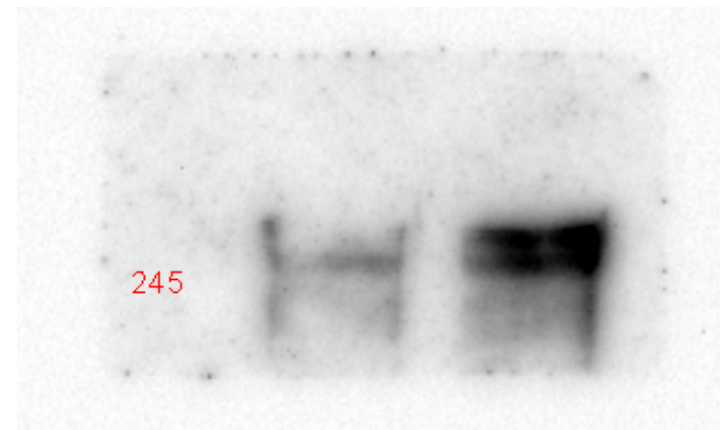


Ki-67

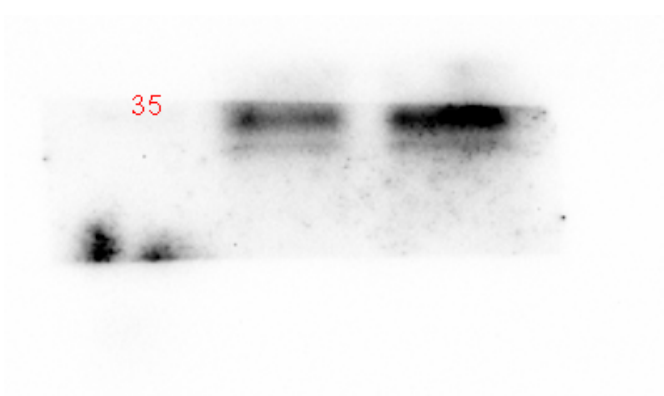


SNAI1

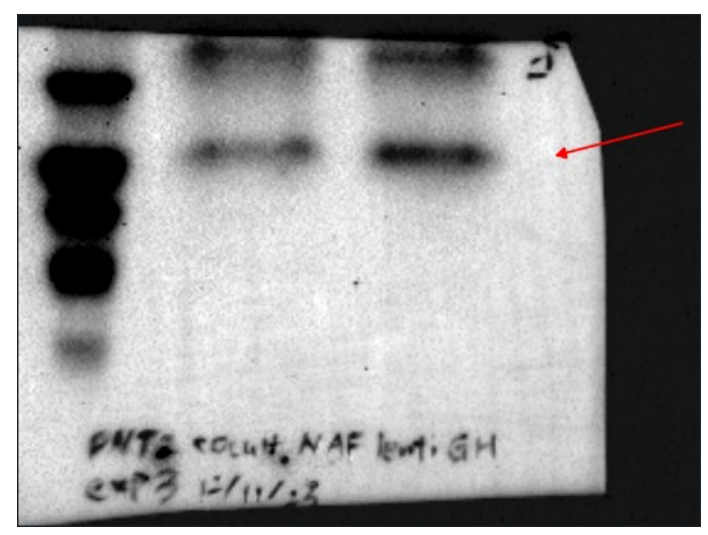

TWIST2

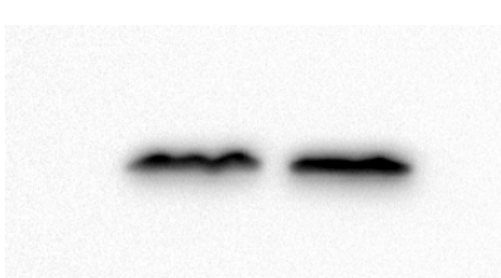

GAPDH-1for Twist2

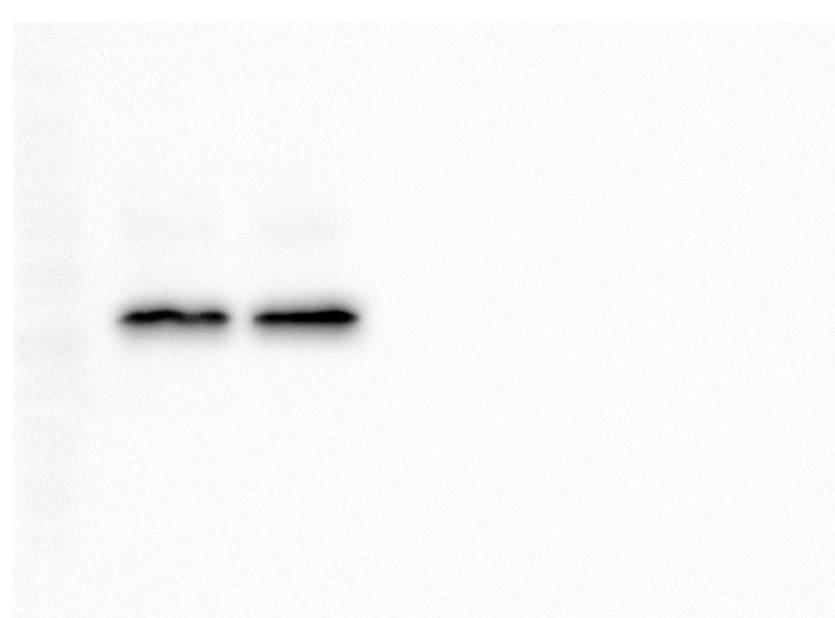

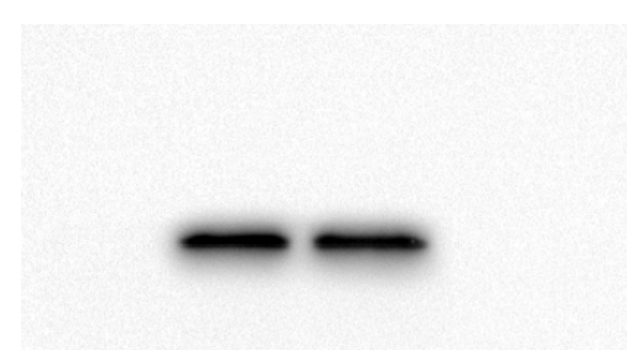

GAPDH-3 for Snai1

GAPDH-2 forE cadherin and Ki67

# Full unedited gels for Figure 4D

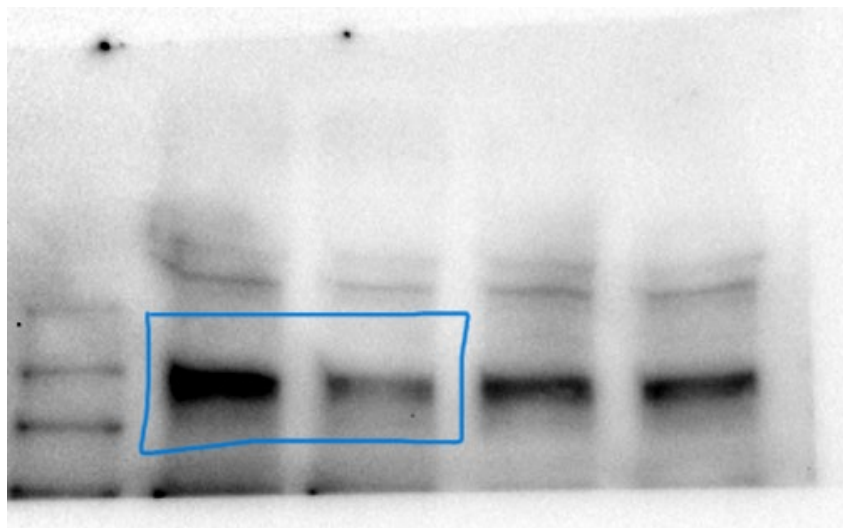

GHR

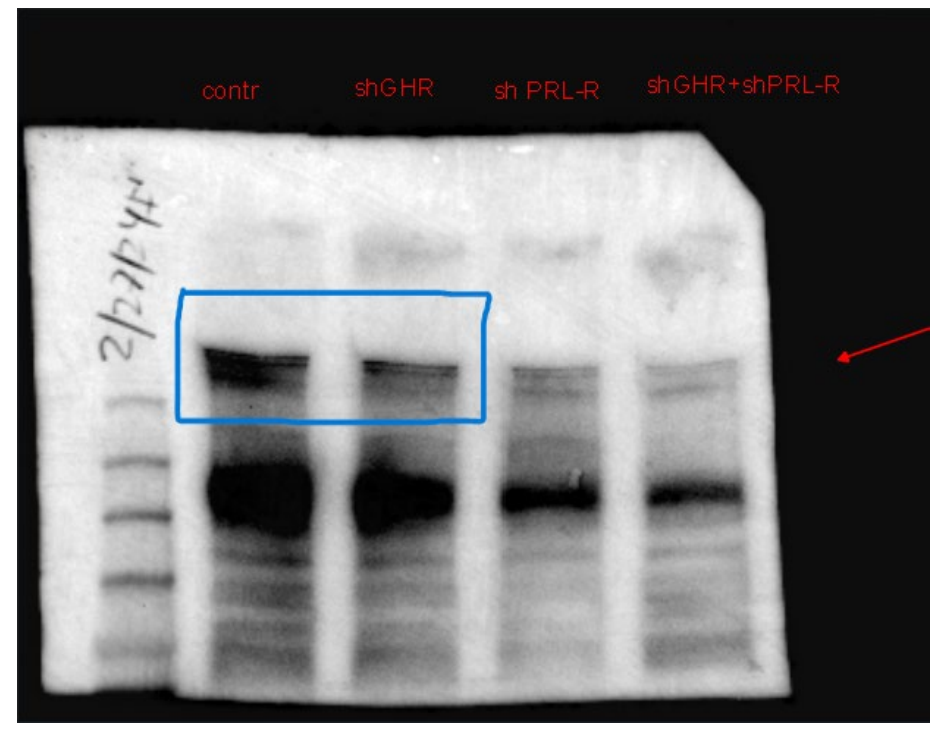


Ki-67

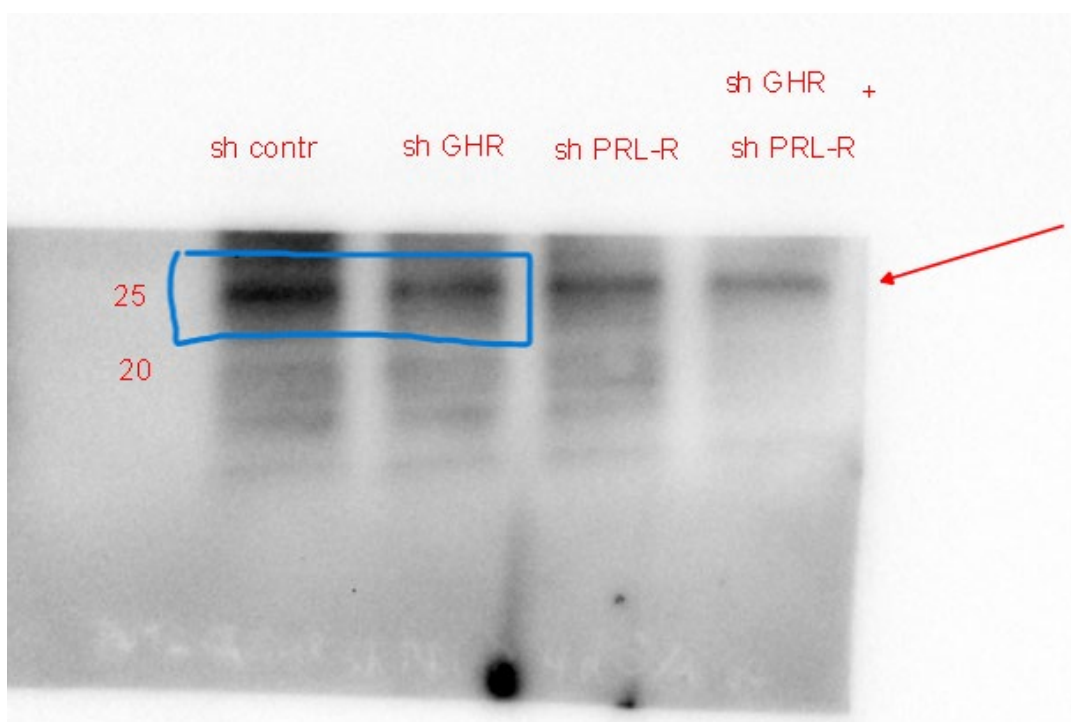


TWIST2

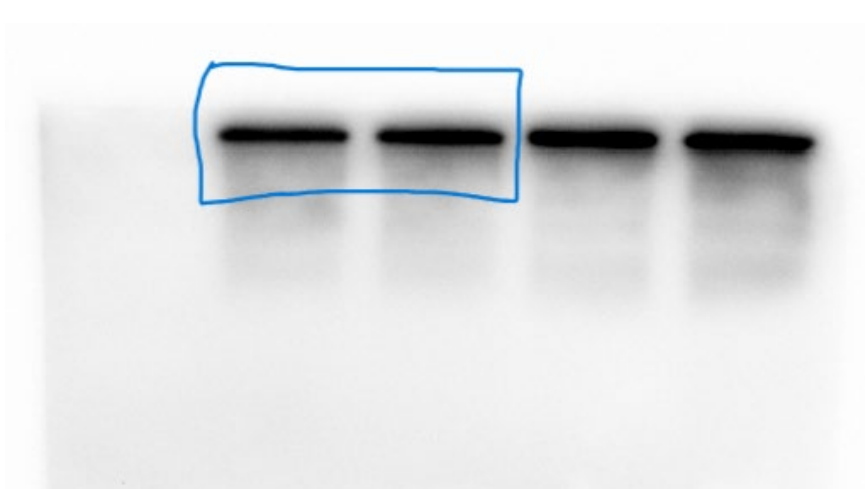

GAPDH-1 for GHR and Twist2

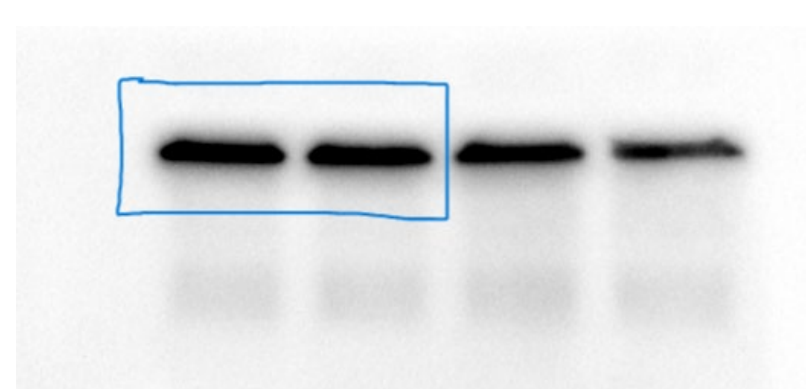

GAPDH-2 for Ki67

# Full unedited gels for Figure 4E, 4F

## Figure 4E

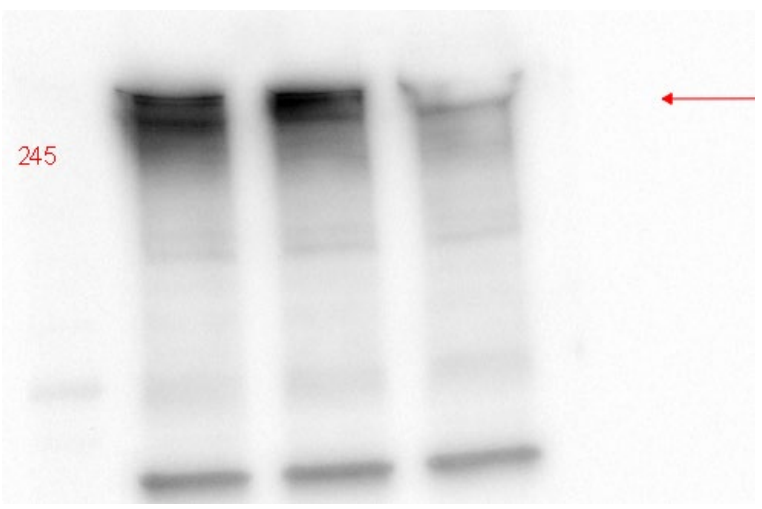


Ki-67

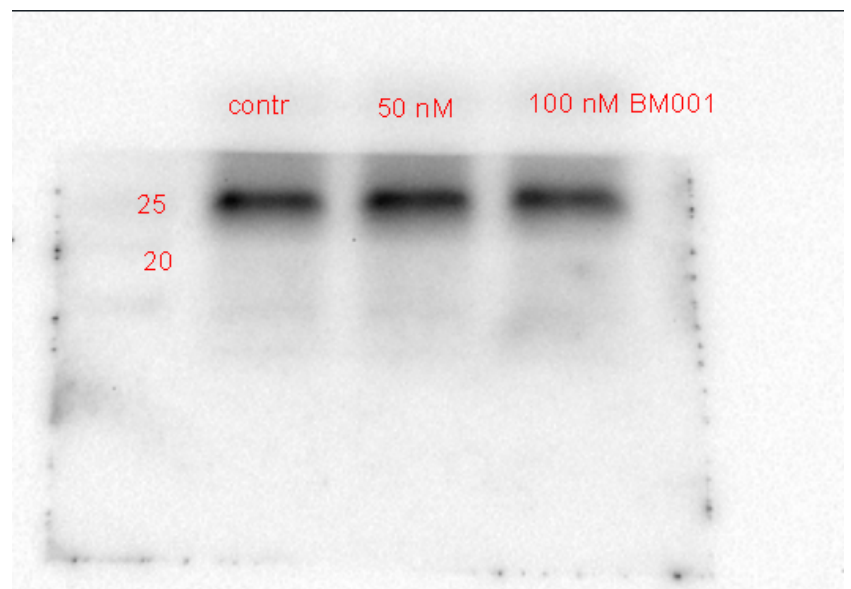


Twist2

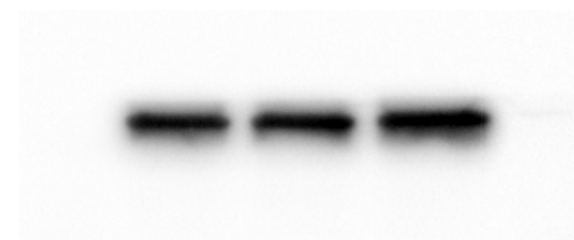

GAPDH

---

## Figure 4F

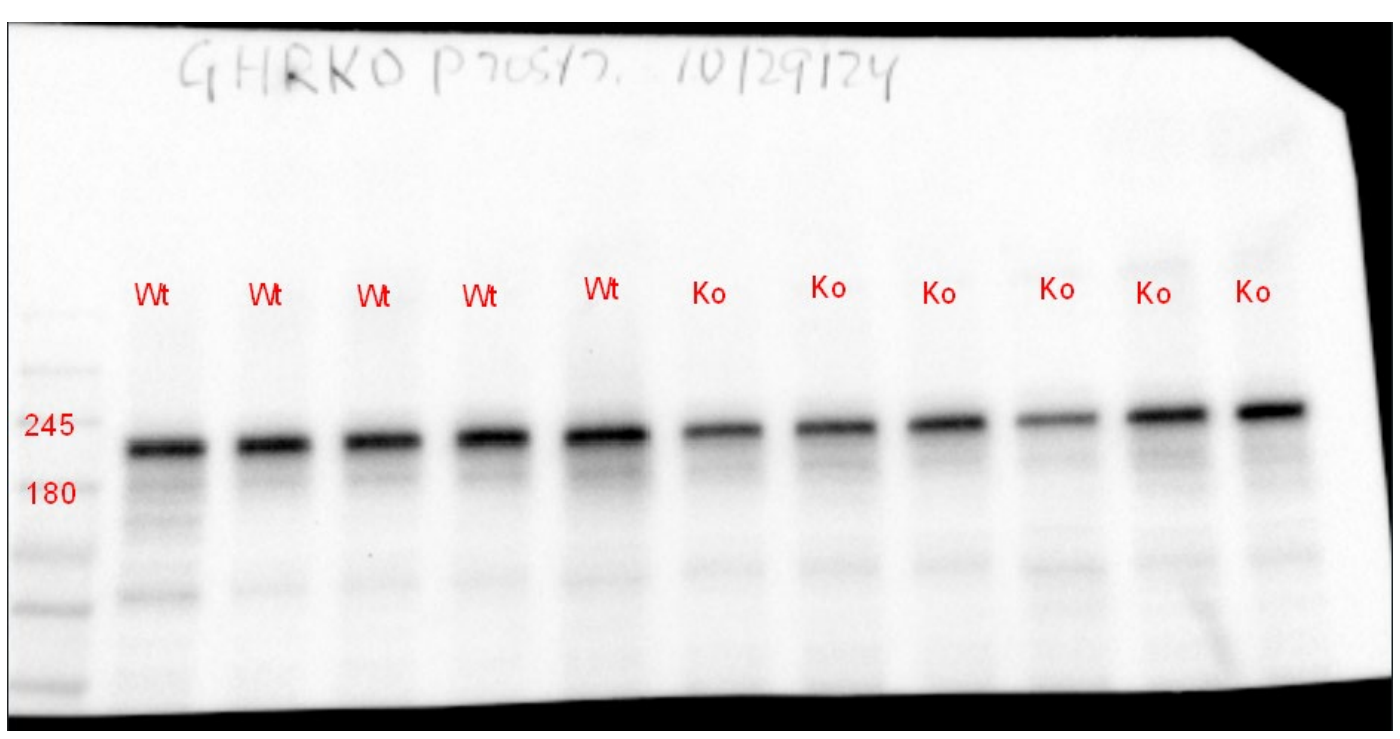


Ki67

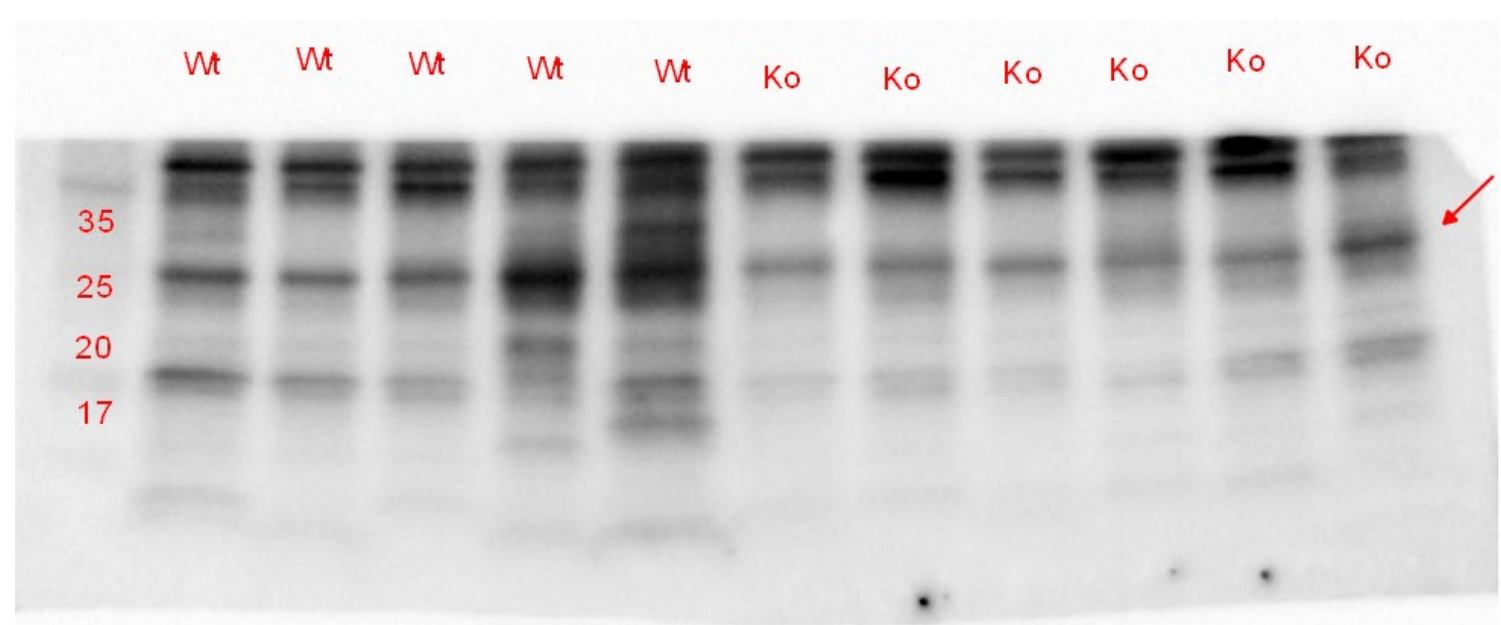


Snai1

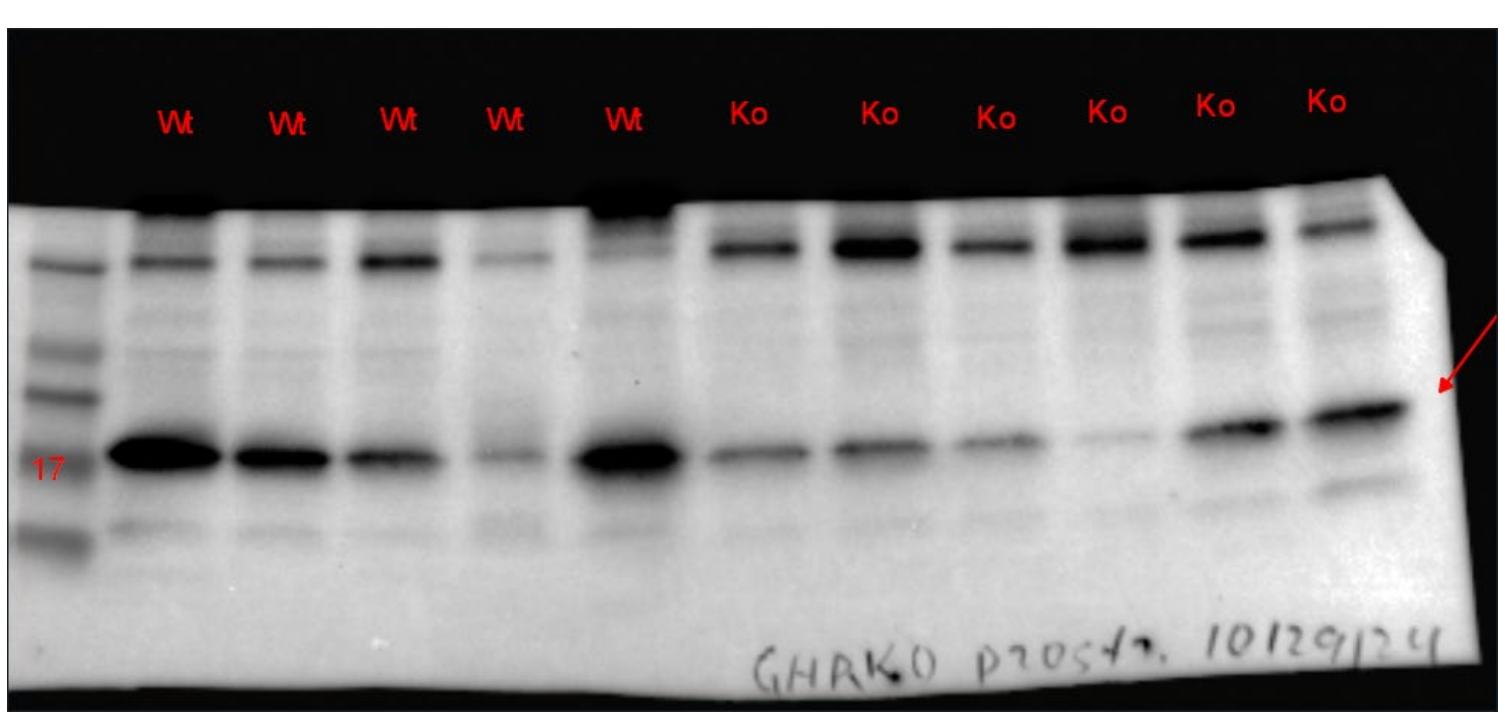


Twist2

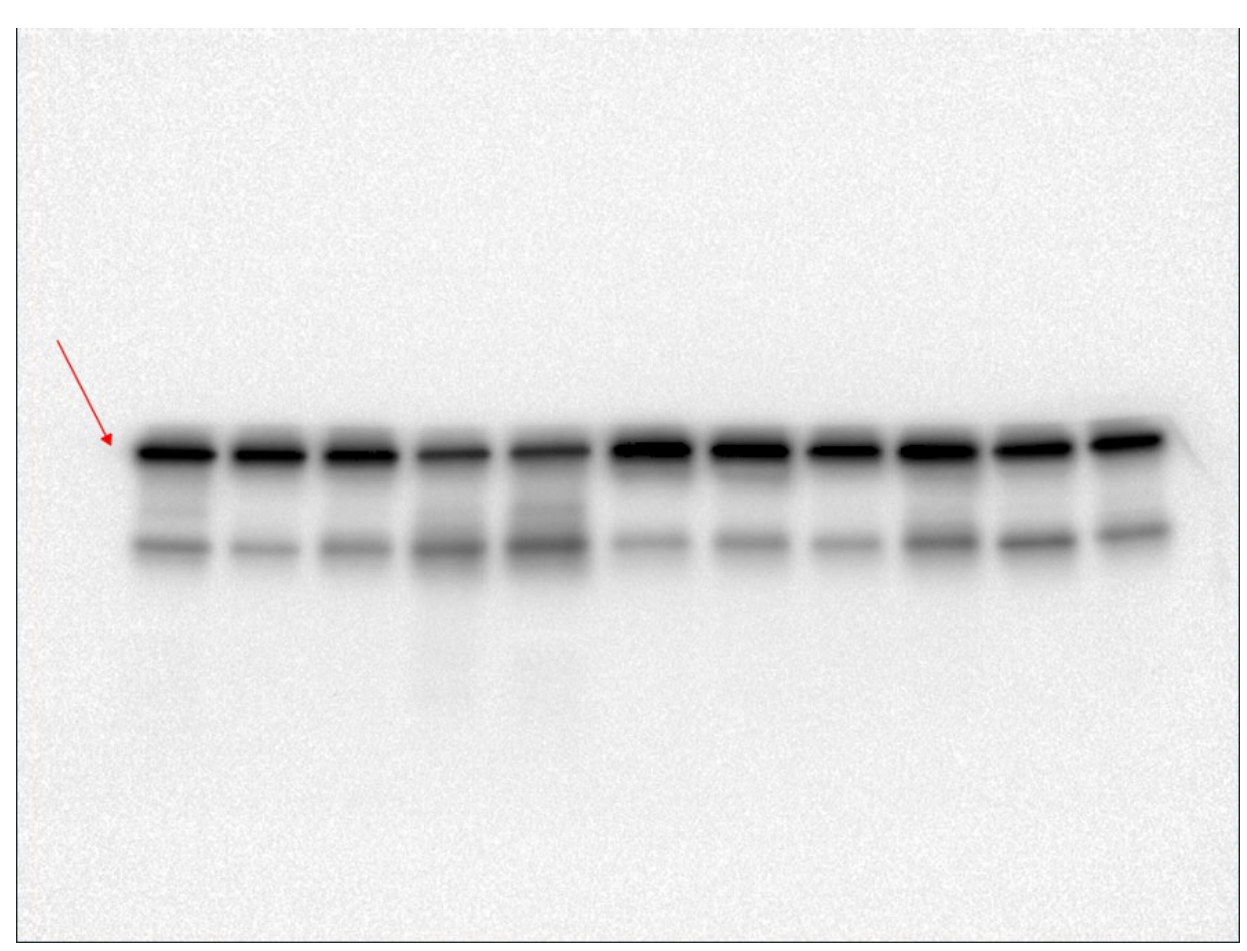

GAPDH

# Full unedited gels for Figure 5A, 5C

## Figure 5A

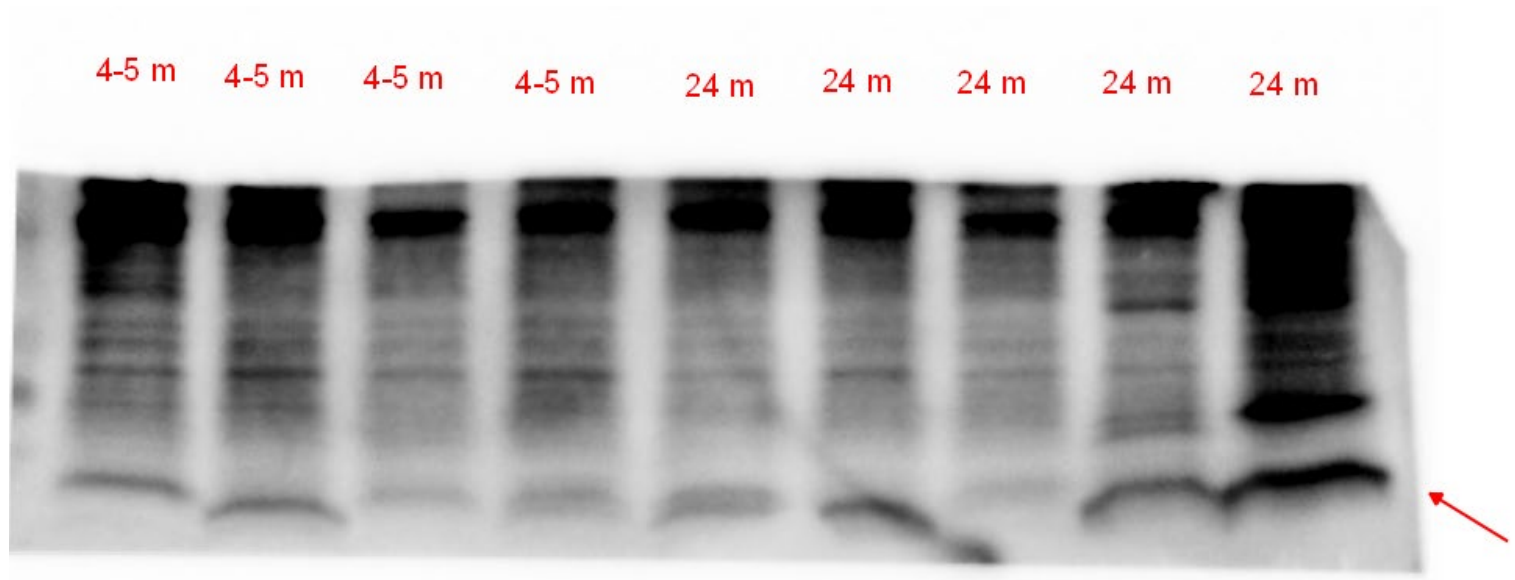


IGF-1

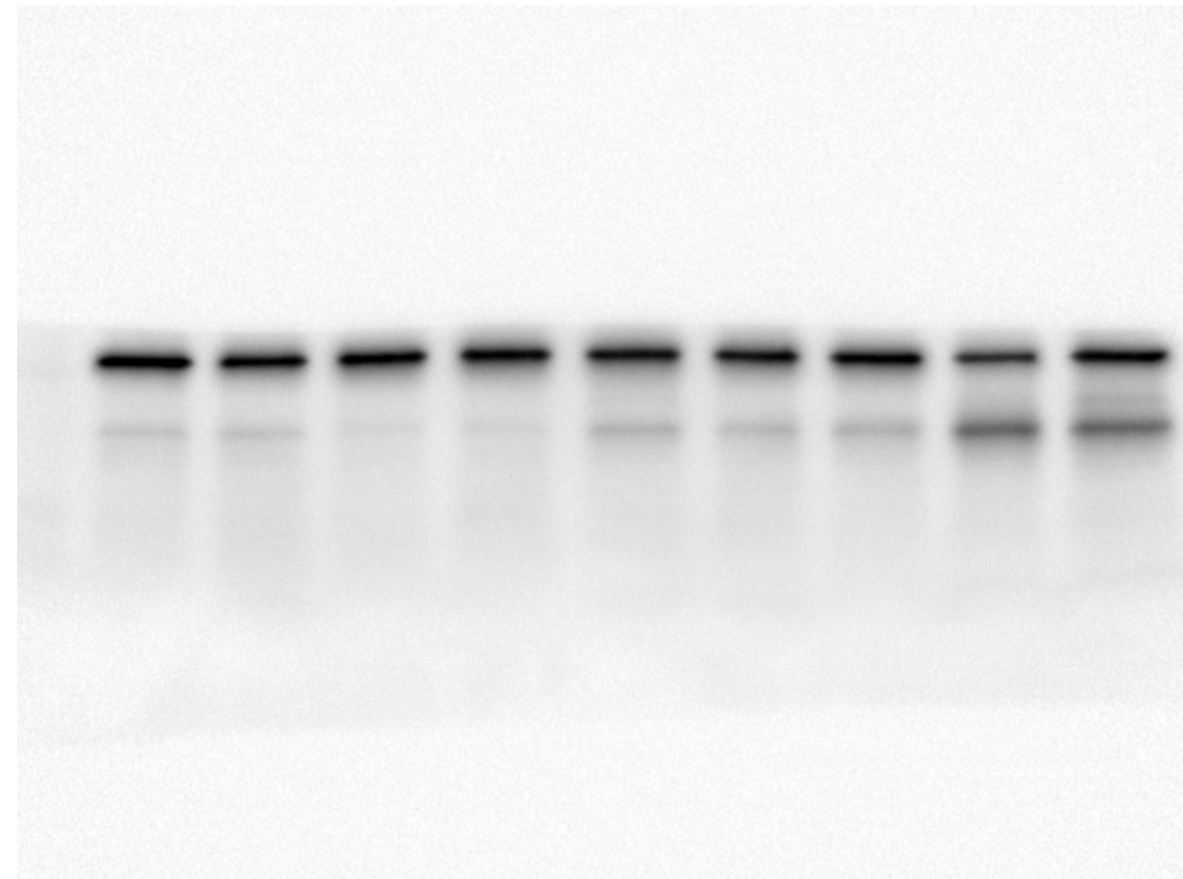

GAPDH

---

## Figure 5C

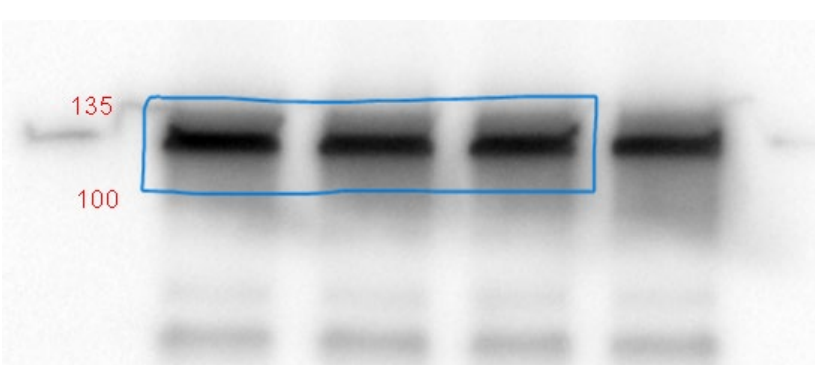


E-cadherin

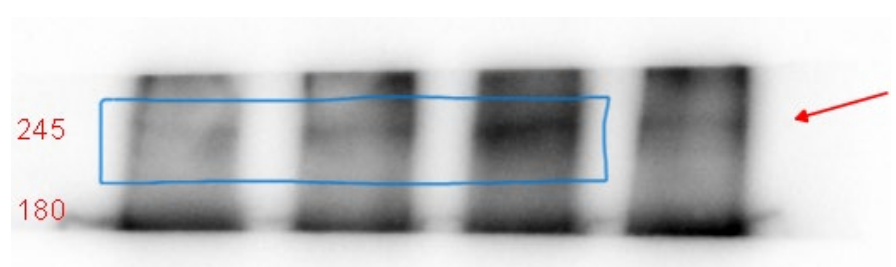


Ki-67

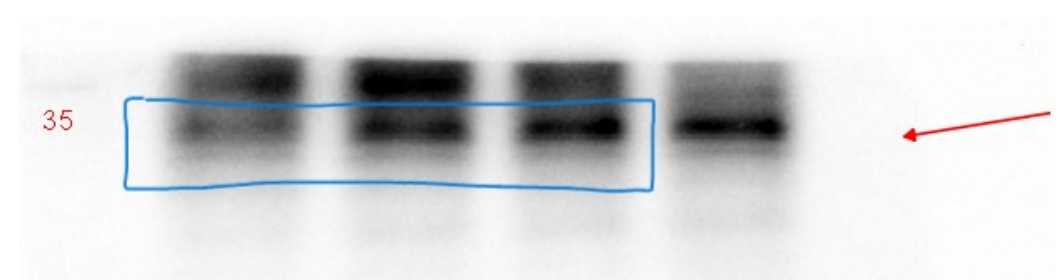


SNAI1

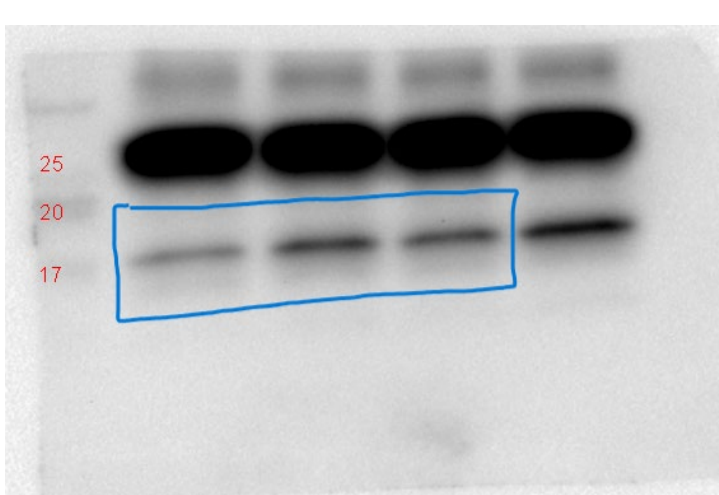


TWIST2

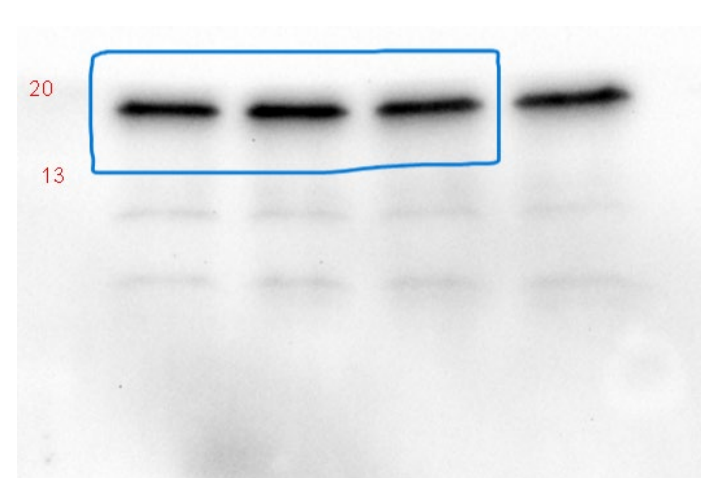


γH2AX

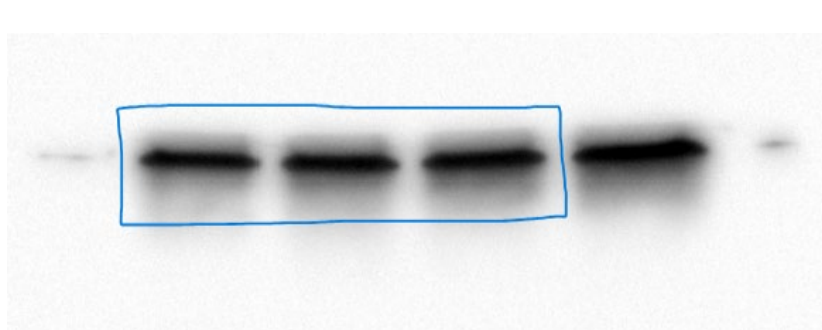

GAPDH-1 for H2AX

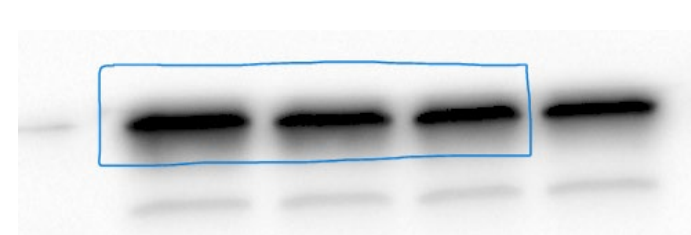

GAPDH-2 for Twist2, E cadherin,Snai1, Ki67

# Full unedited gels for Figure 5D

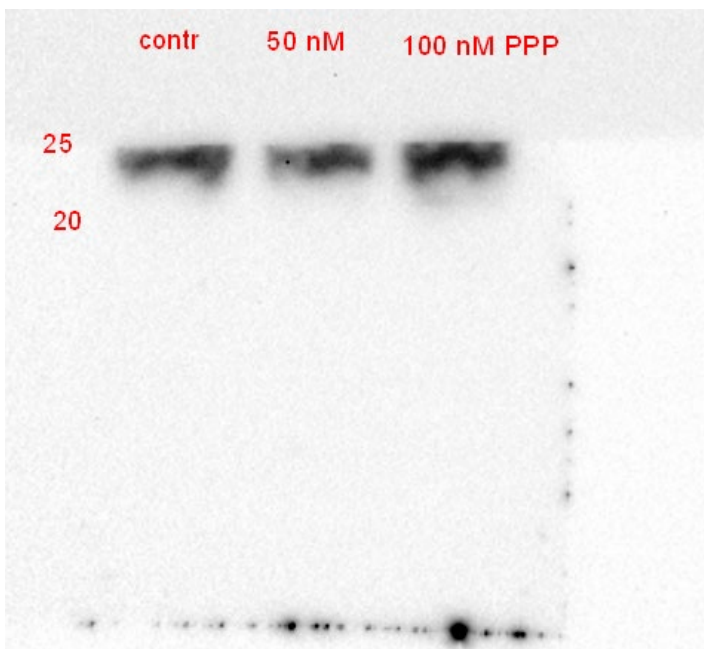


GH

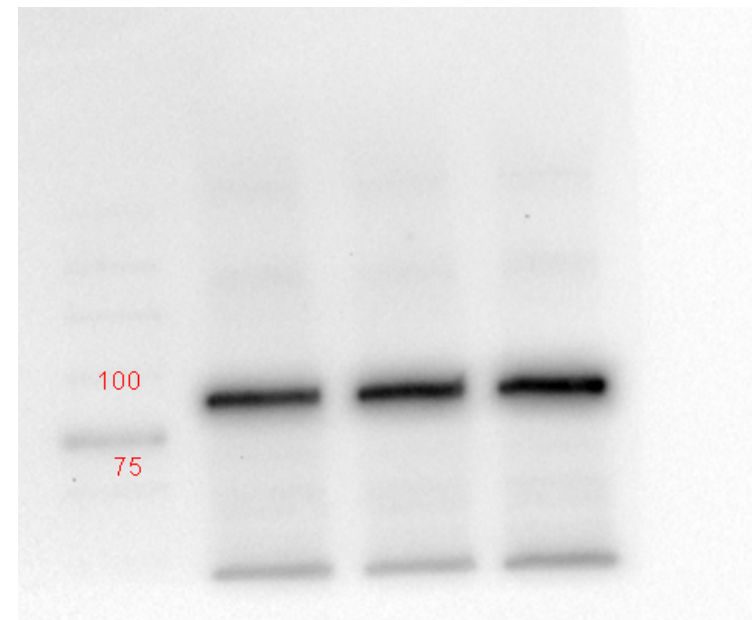


GHR

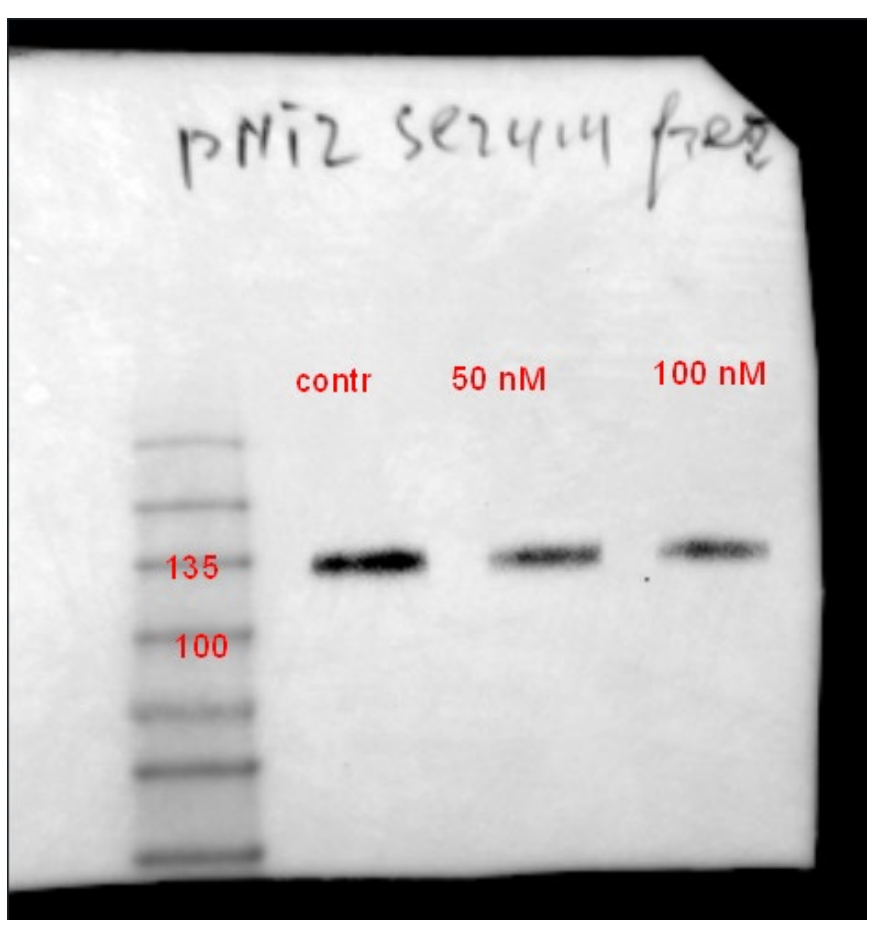


pIGF1-R

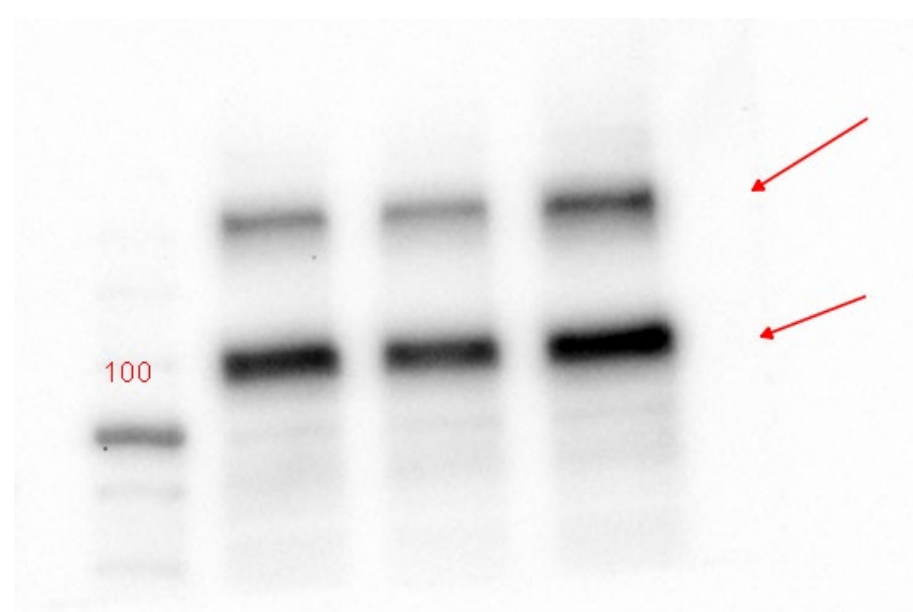


Total IGF1-R

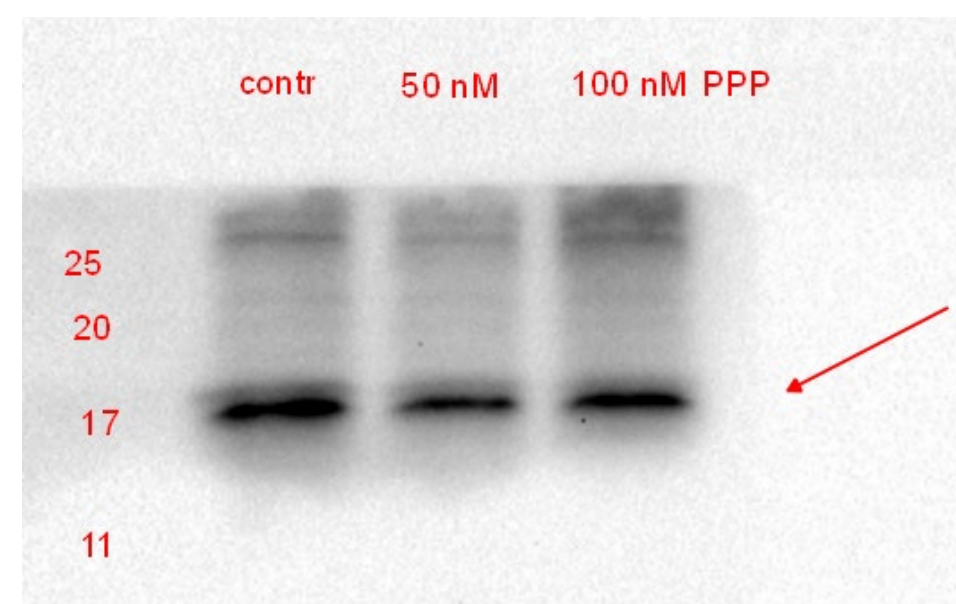


TWIST2

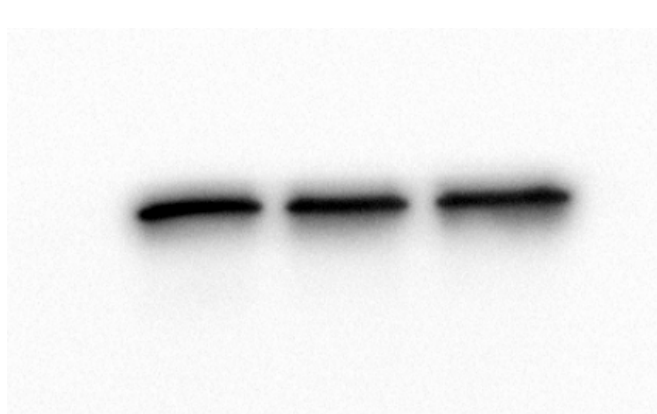

GAPDH-1for tIGF1R

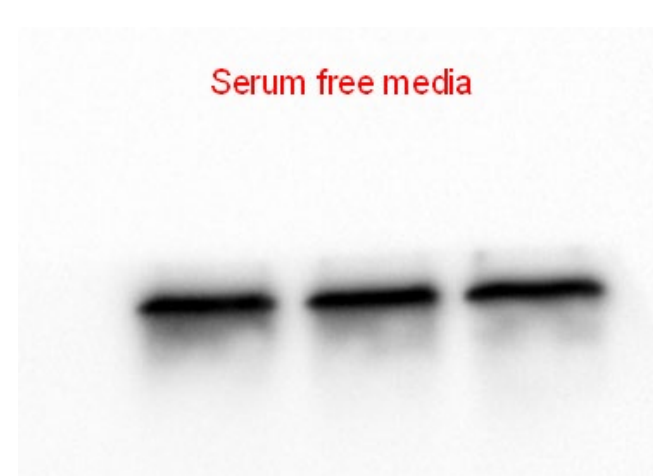


GAPDH-2pIGF1R, GH, GHR, Twist

# Full unedited gels for Figure 5E

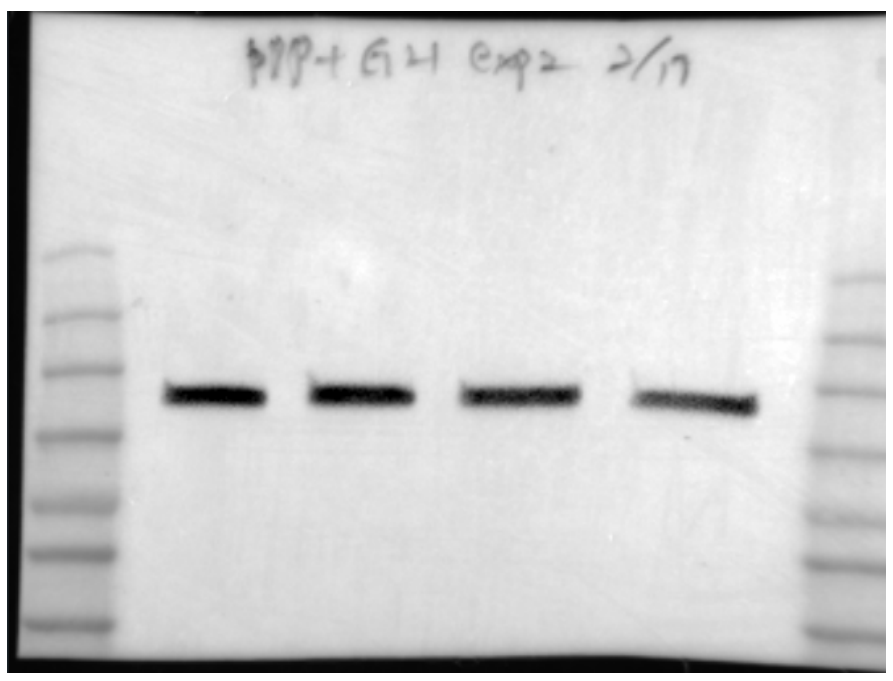

E-cadherin

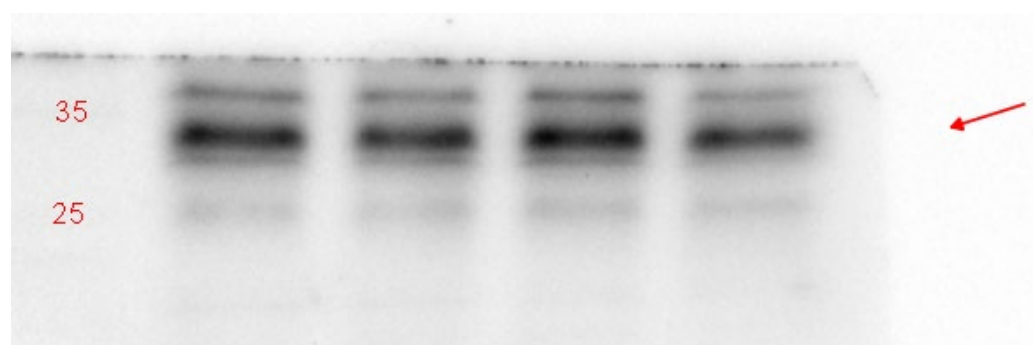


Snai1

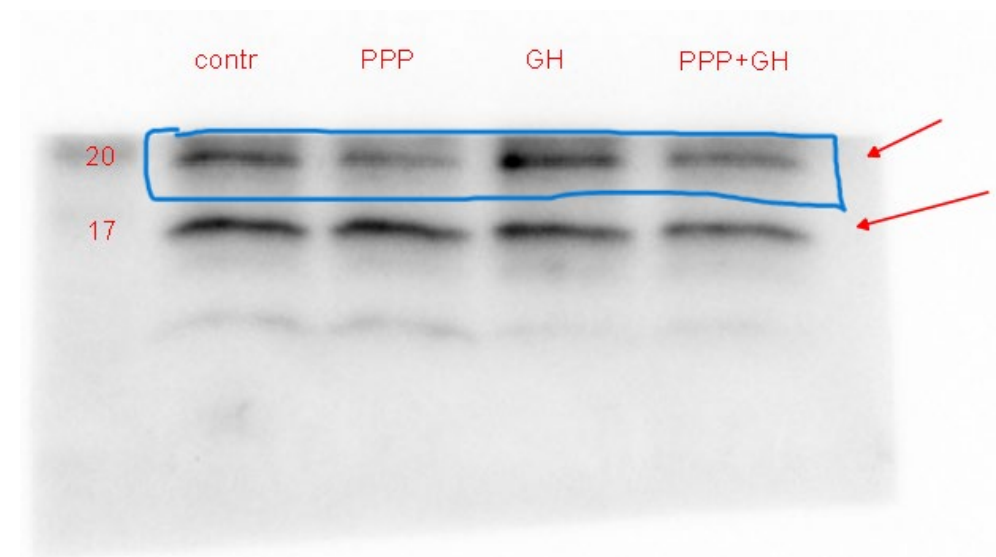


Twist2

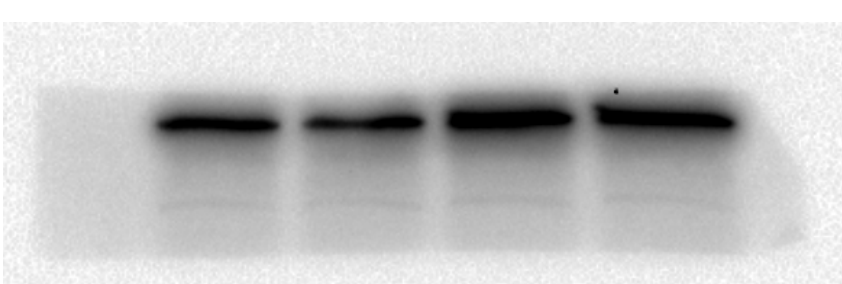

GAPDH-1 for Twist2

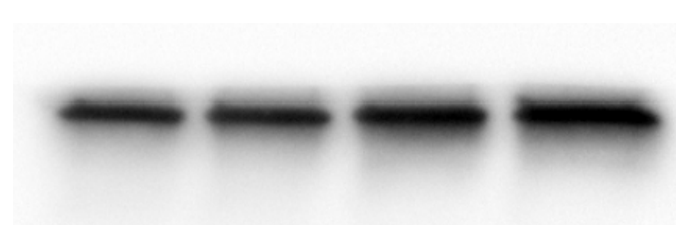

GAPDH-2 for E cadherin

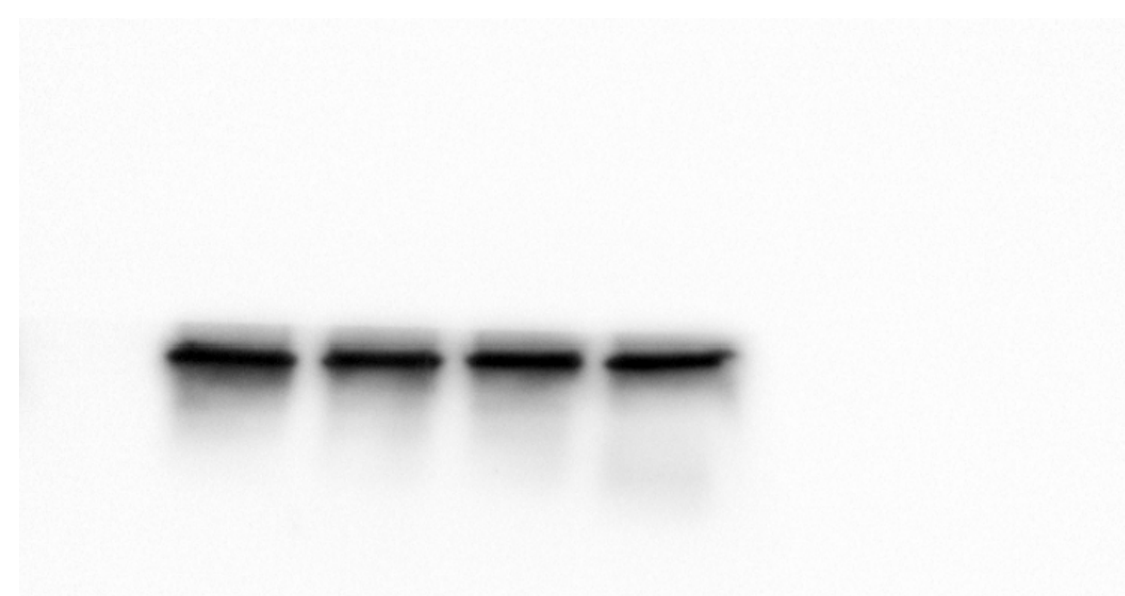

GAPDH-3fro Snai1

# Full unedited gels for Figure 5F

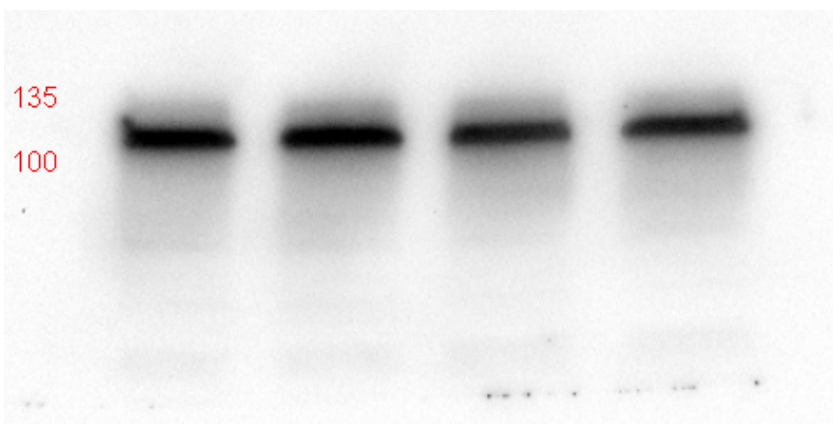


E-cadherin

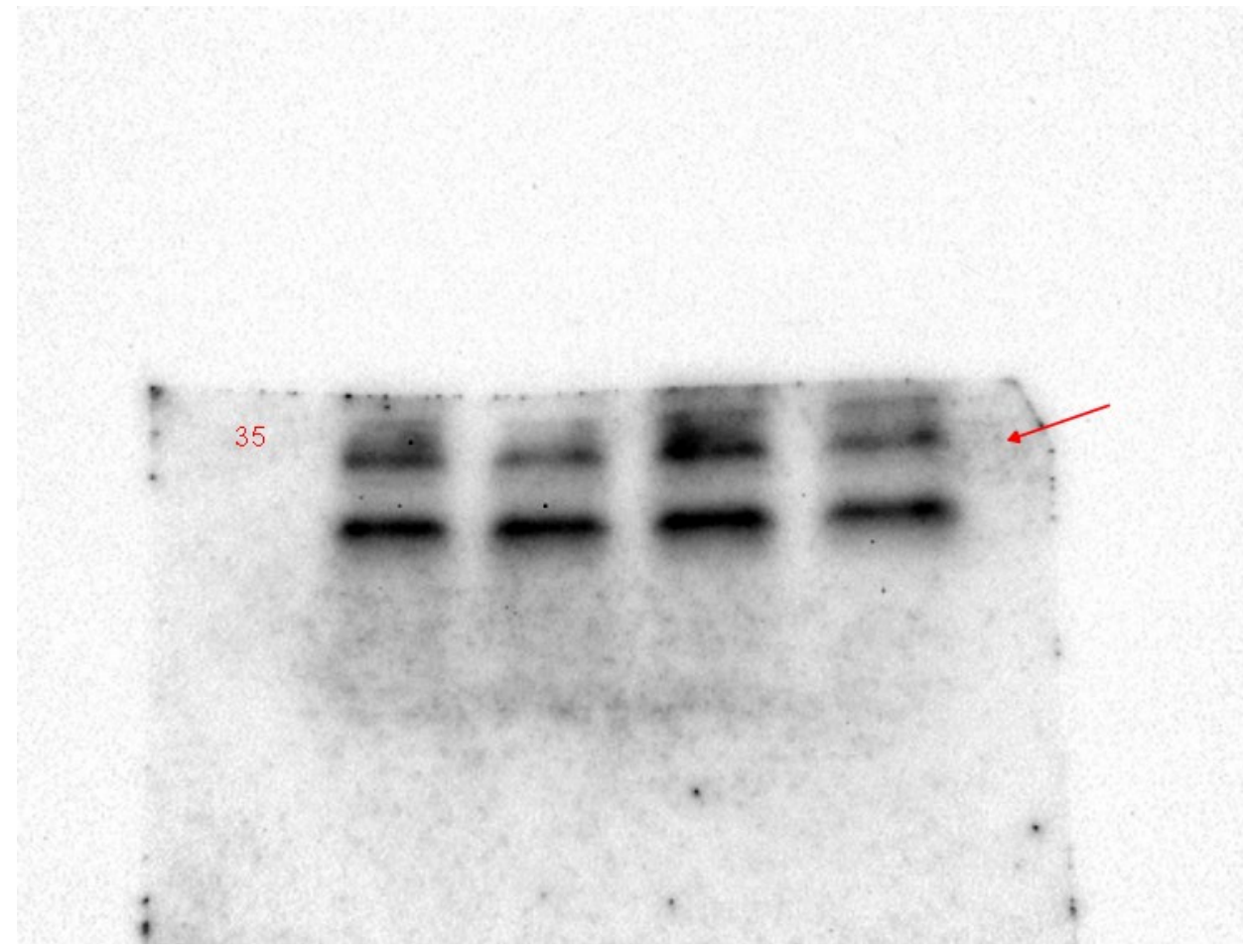


Snai1

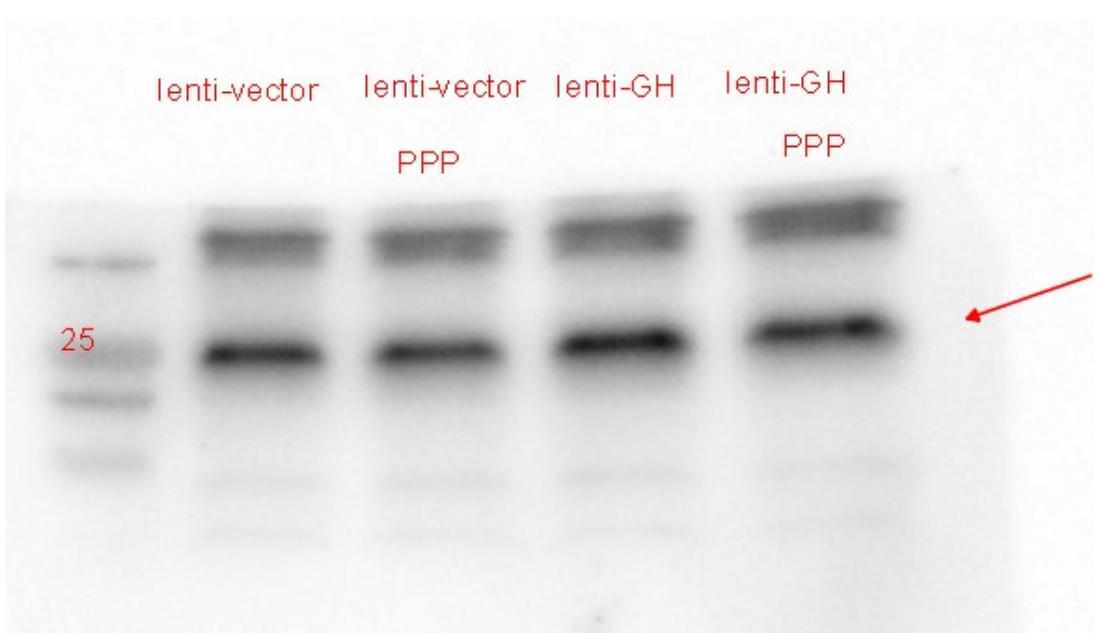


Twist2

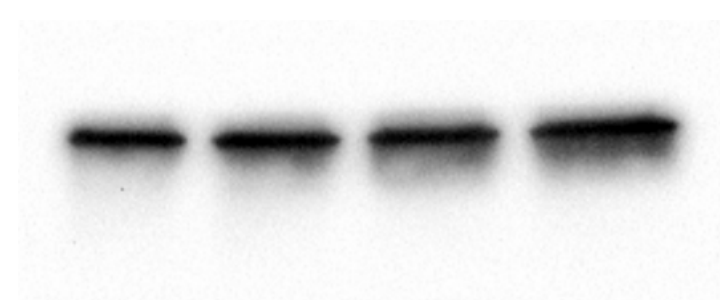

GAPDH

# Full unedited gels for Figure 6E

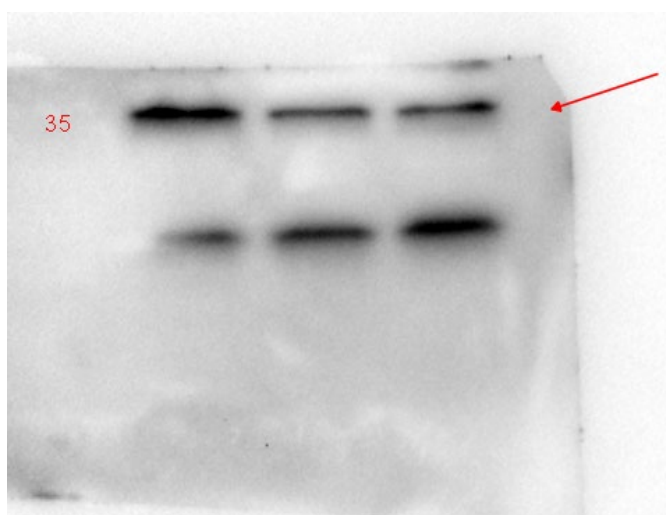


BRCC3

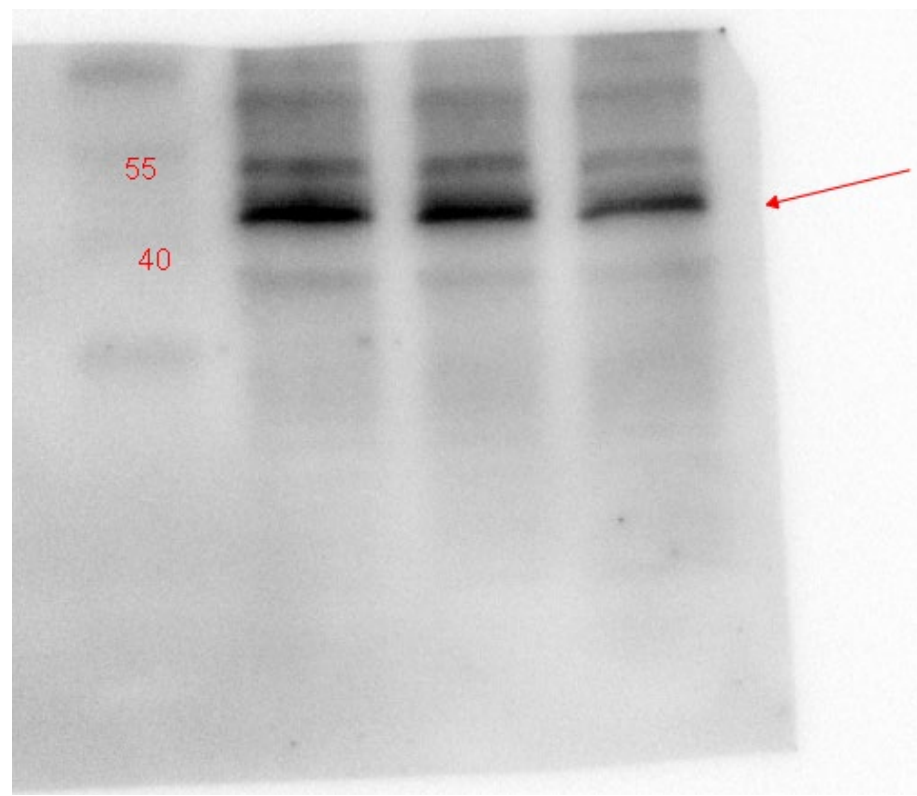


TADA3L

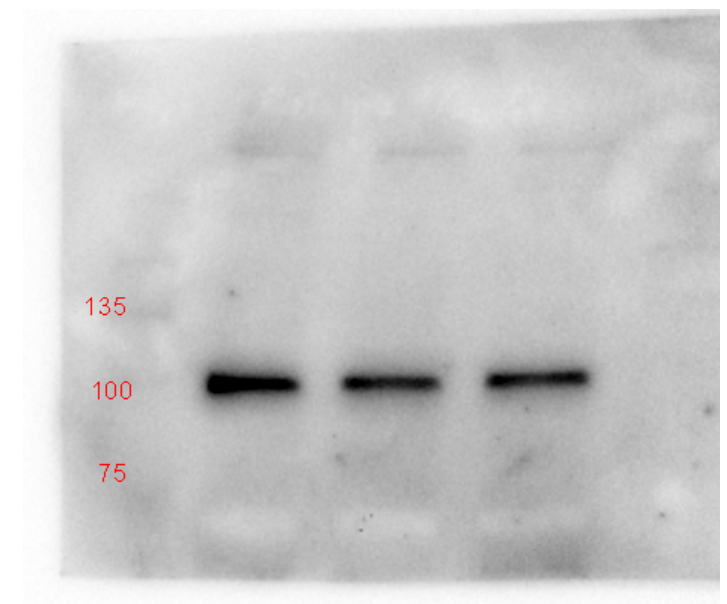


TAF5

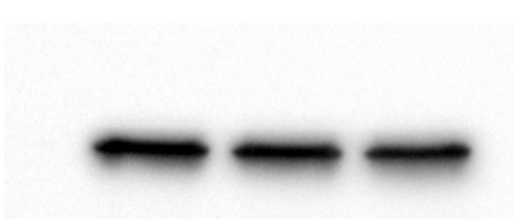

GAPDH-1 for BRCC

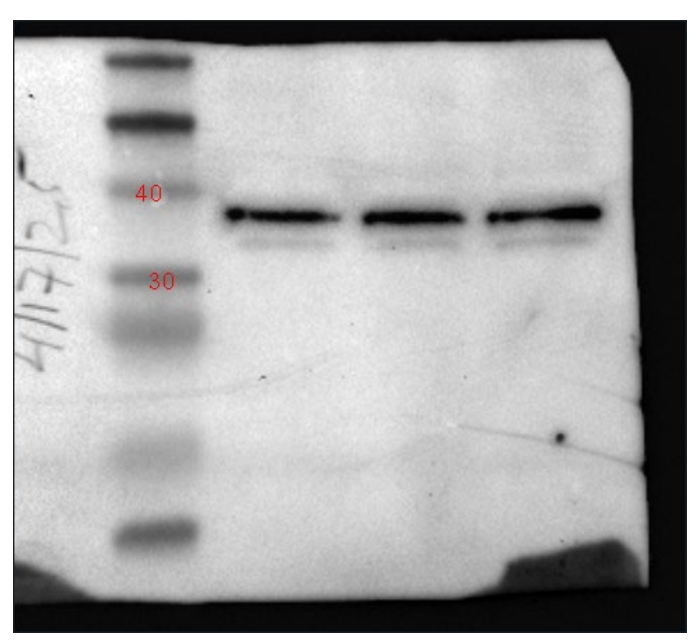


GAPDH-2 for TADA3L

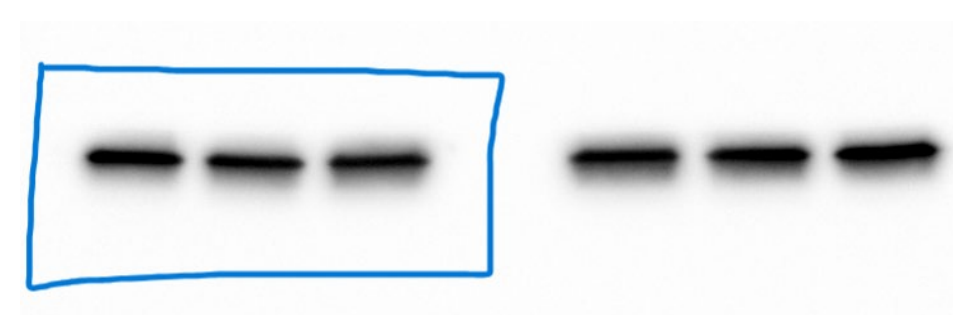

GAPDH-3 for TAF5

# Full unedited gels for Figure 7M

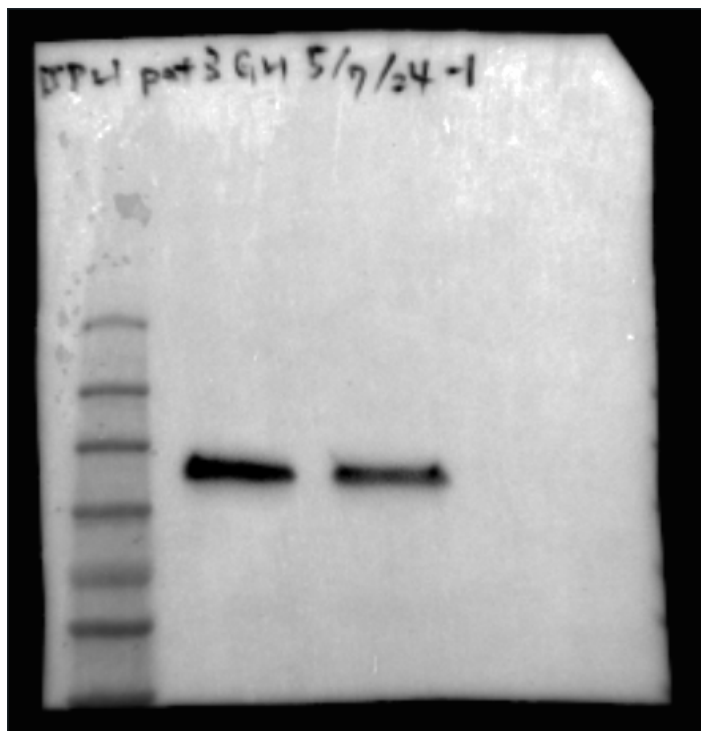

E-cadherin

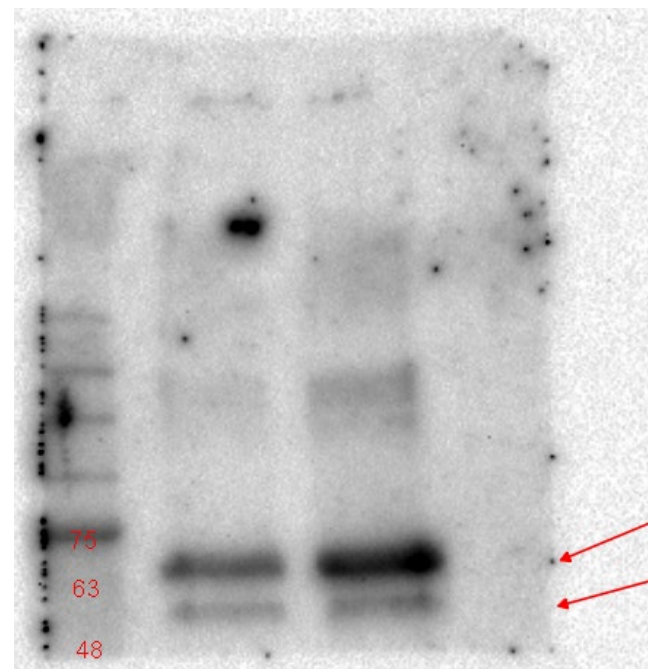


p53

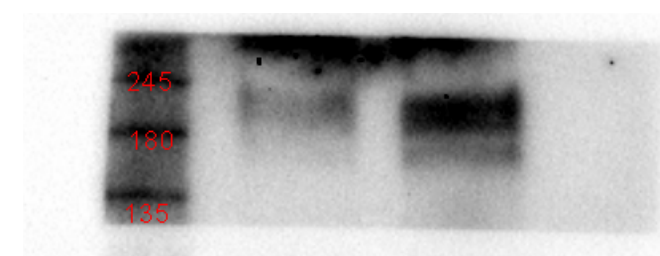


Ki-67

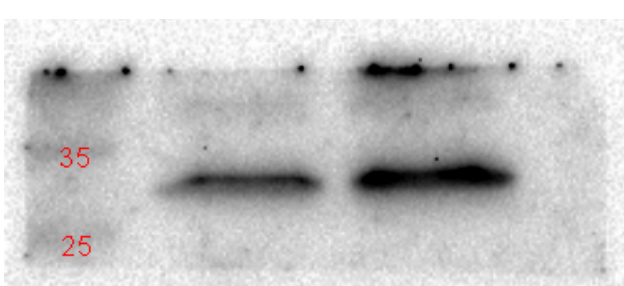


Snai1

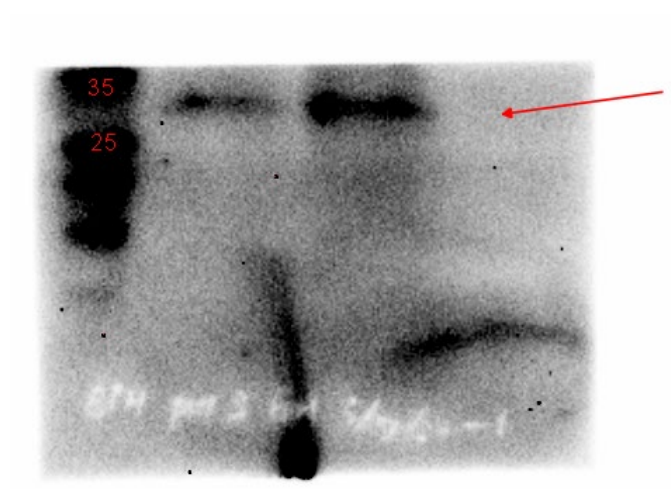


Twist2

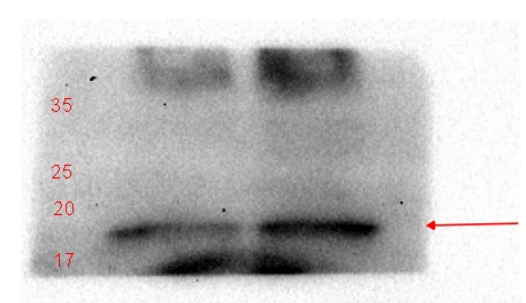


γH2AX

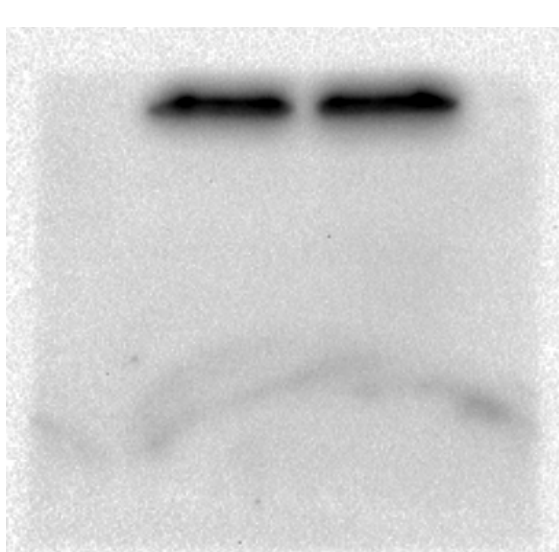

GAPDH-1

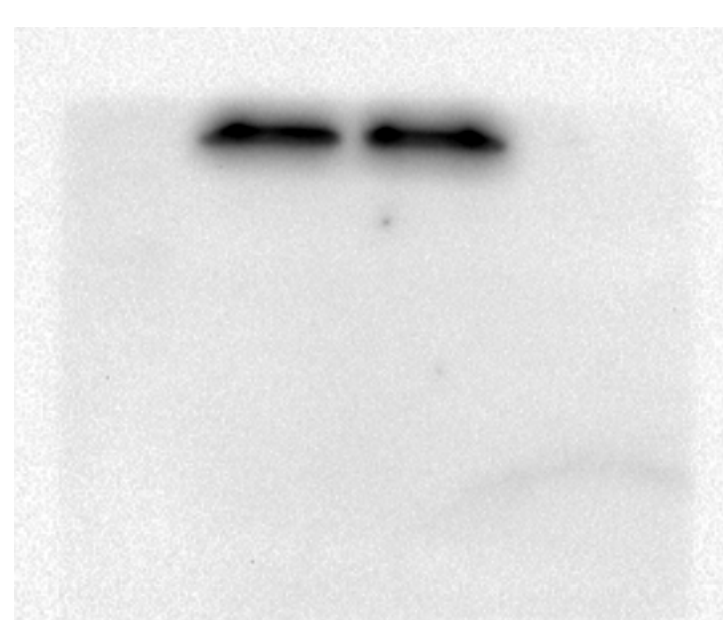

GAPDH-2

# Full unedited gels for Figure 8R

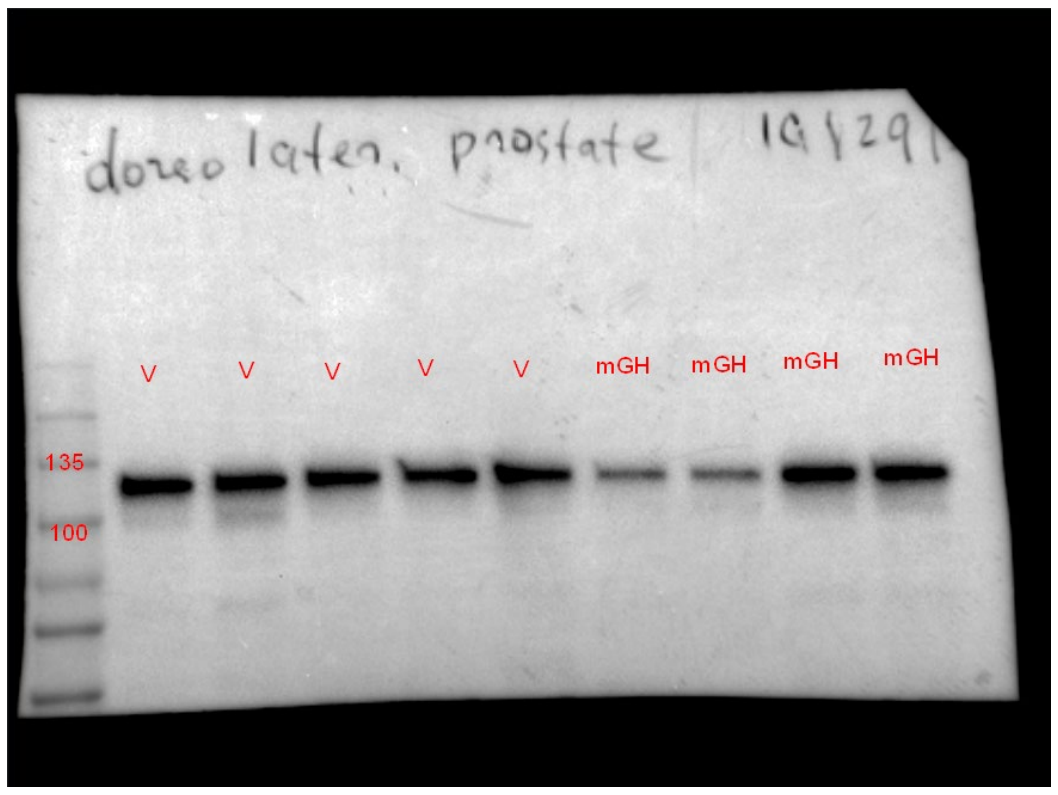


E-cadherin

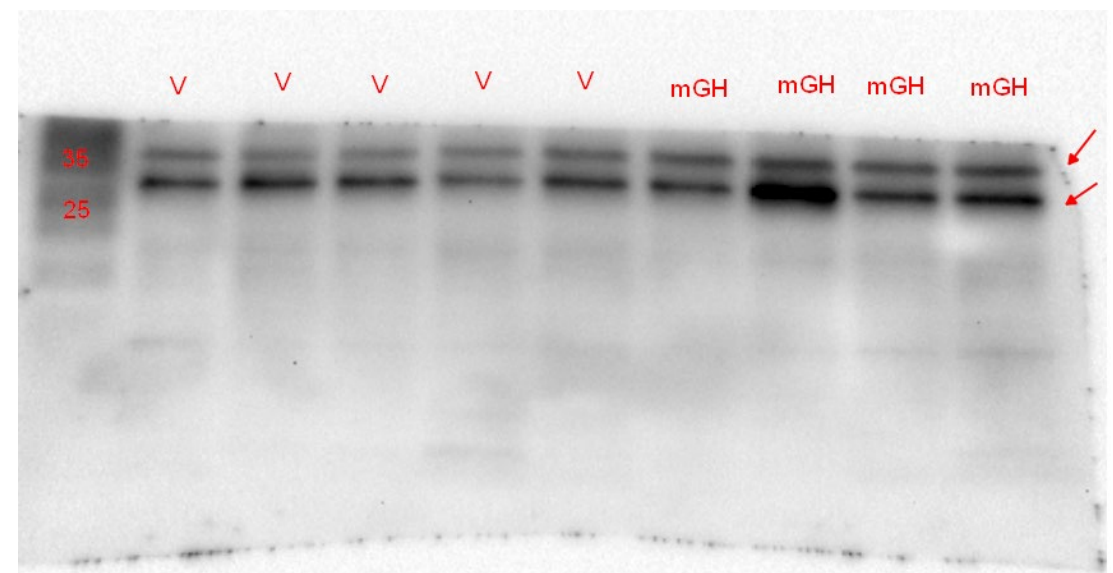


PCNA

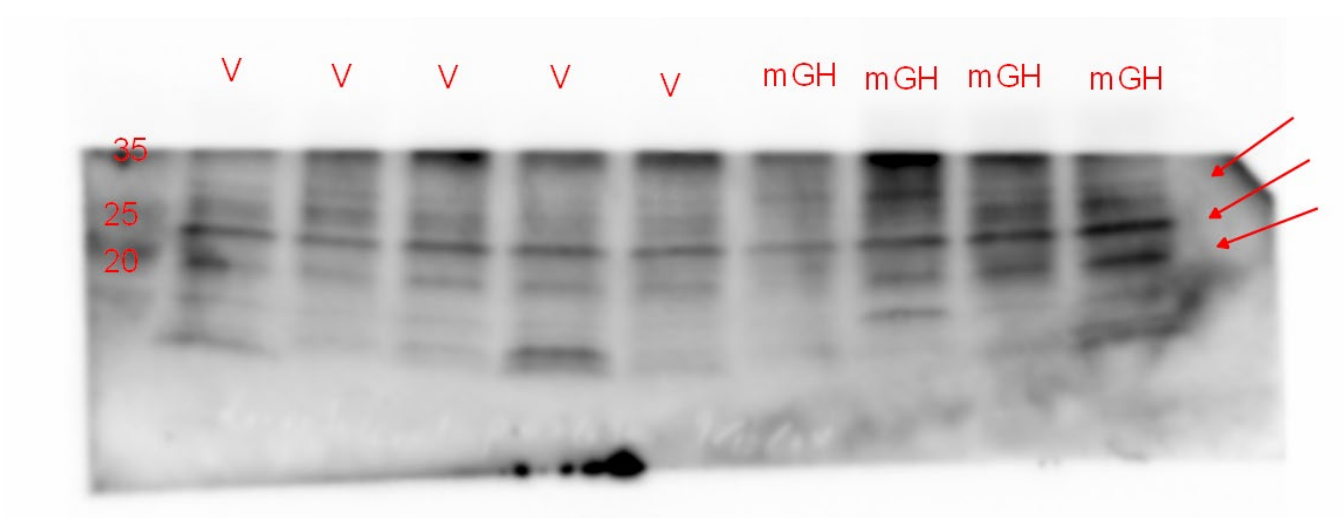


Snai1

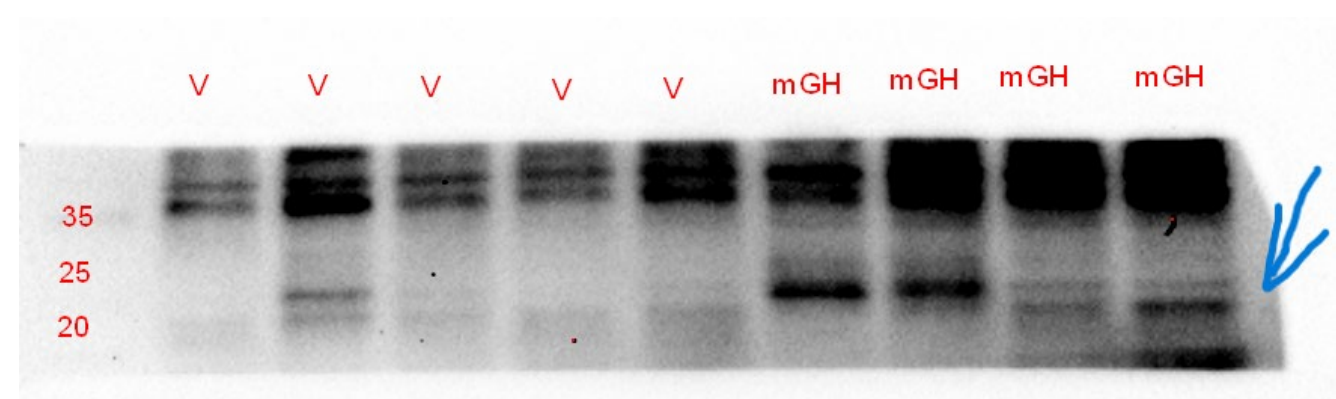


Twist2

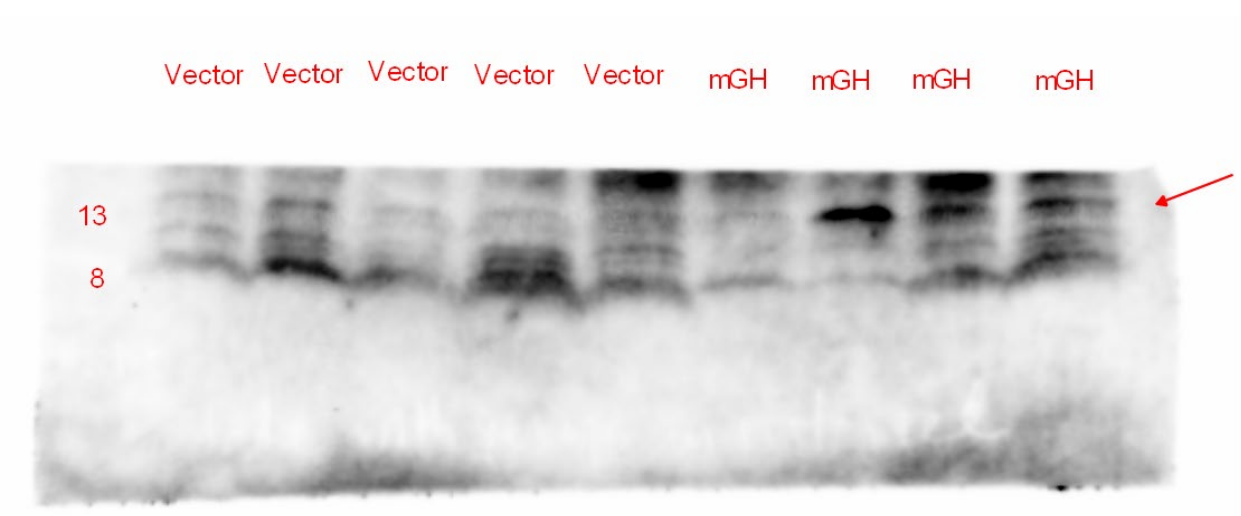


γH2AX

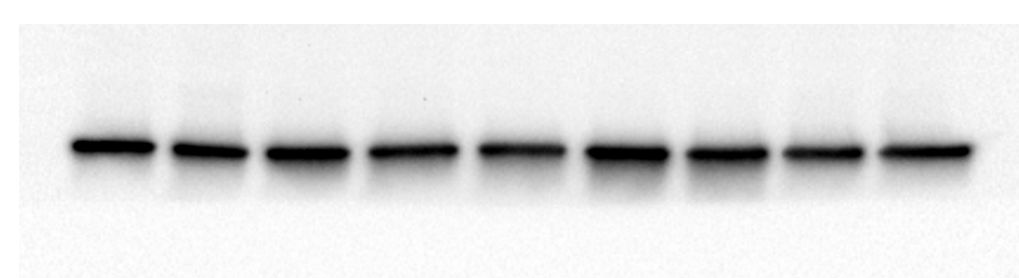

GAPDH-1 for H2AX

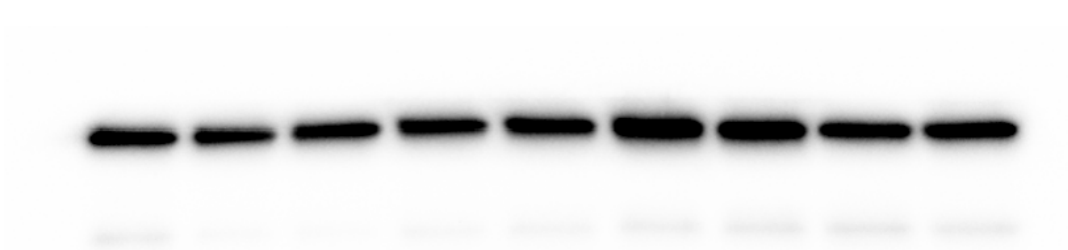

GAPDH-2 for Twist2,E cadherin, PCNA

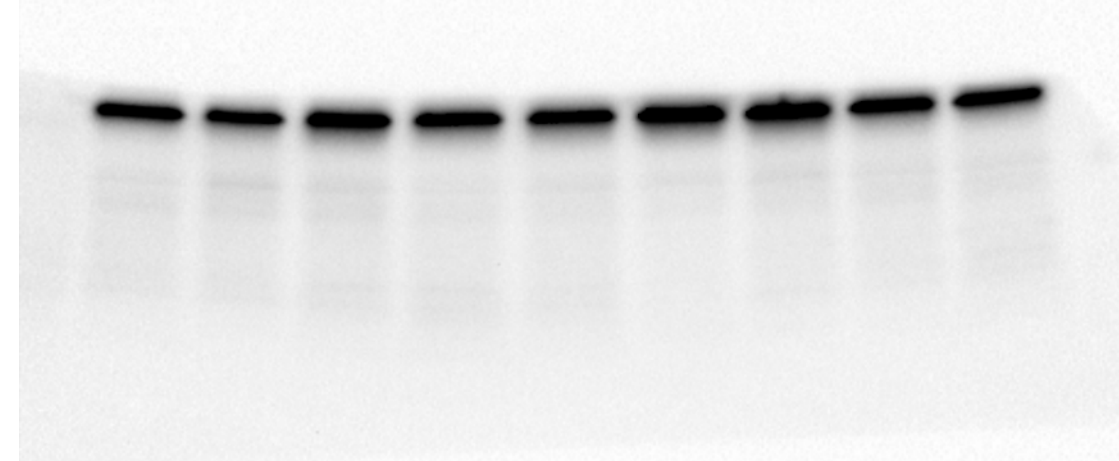

GAPDH-3 for Snai1

# Full unedited gels for Supplemental Figure 2G, S2L

## Supplemental Figure 2G

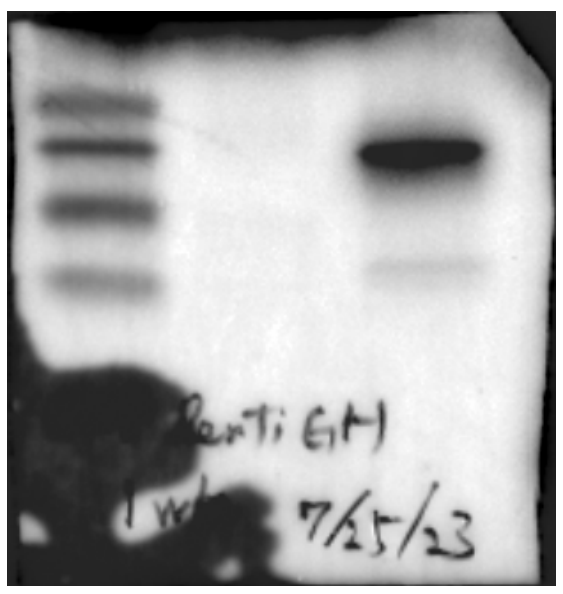

GH

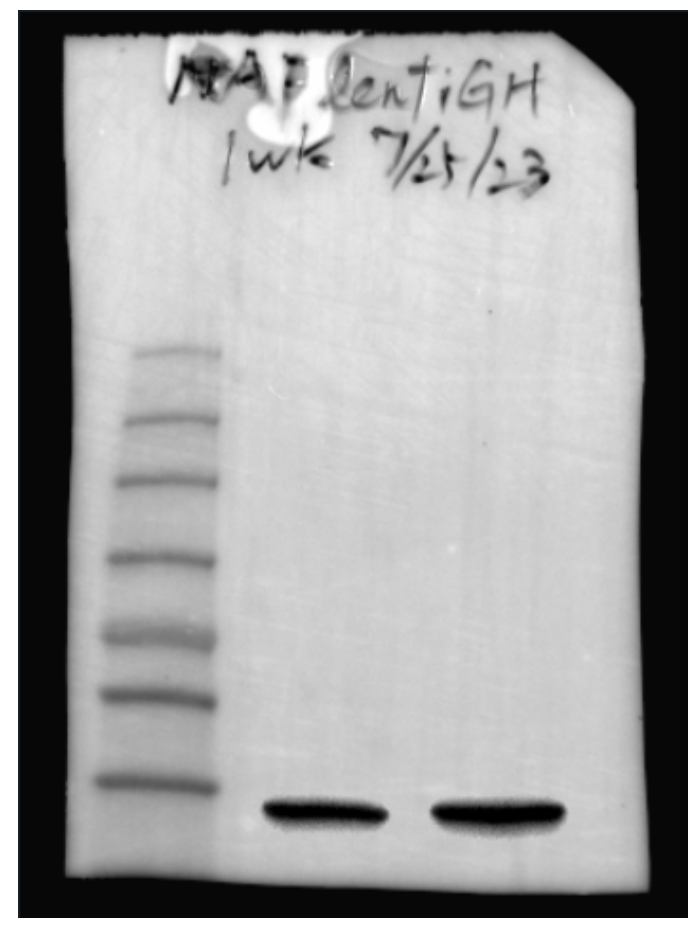

β Actin

---

## Supplemental Figure 2L

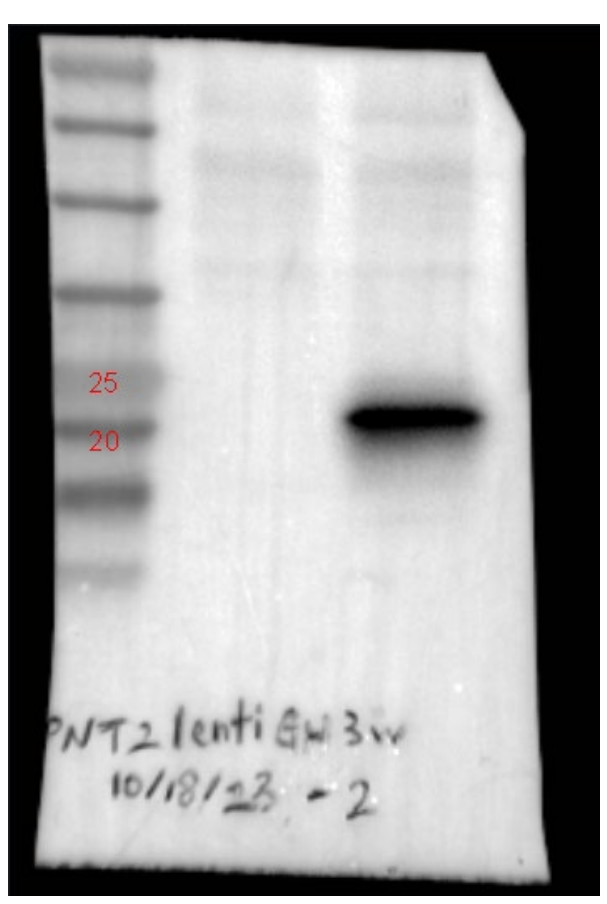


GH

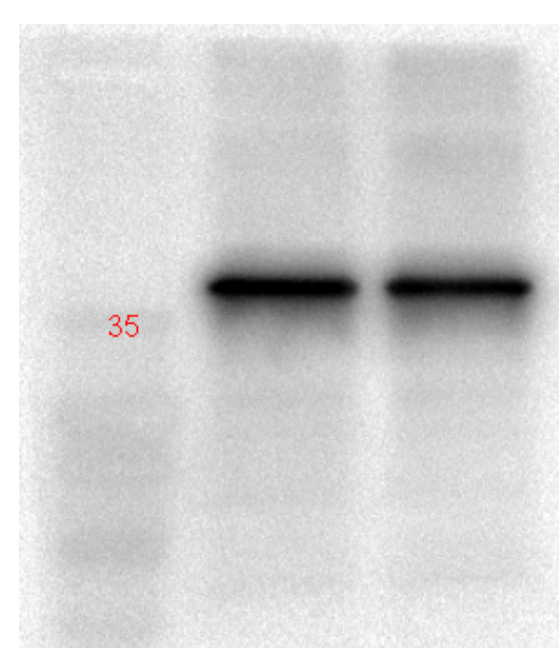


GAPDH

# Full unedited gels for Supplemental Figure 2N

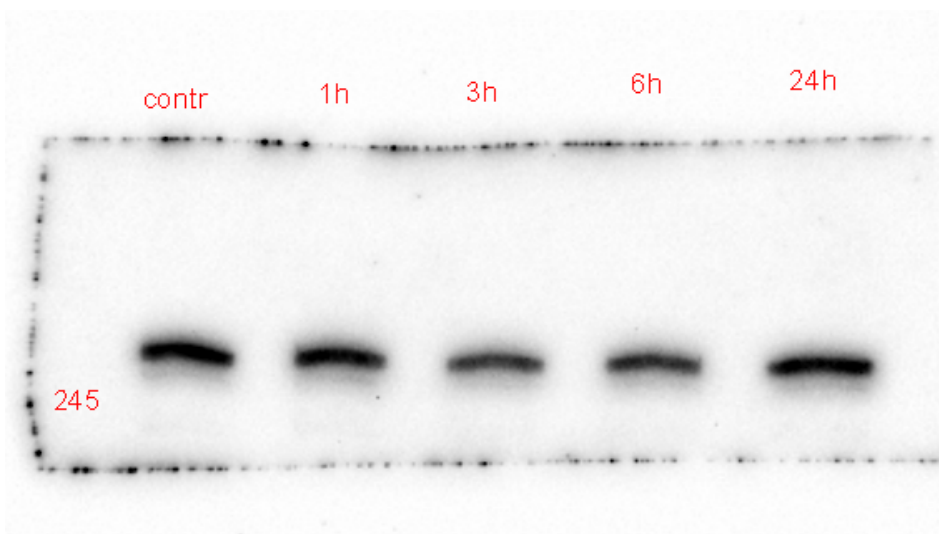


pATM

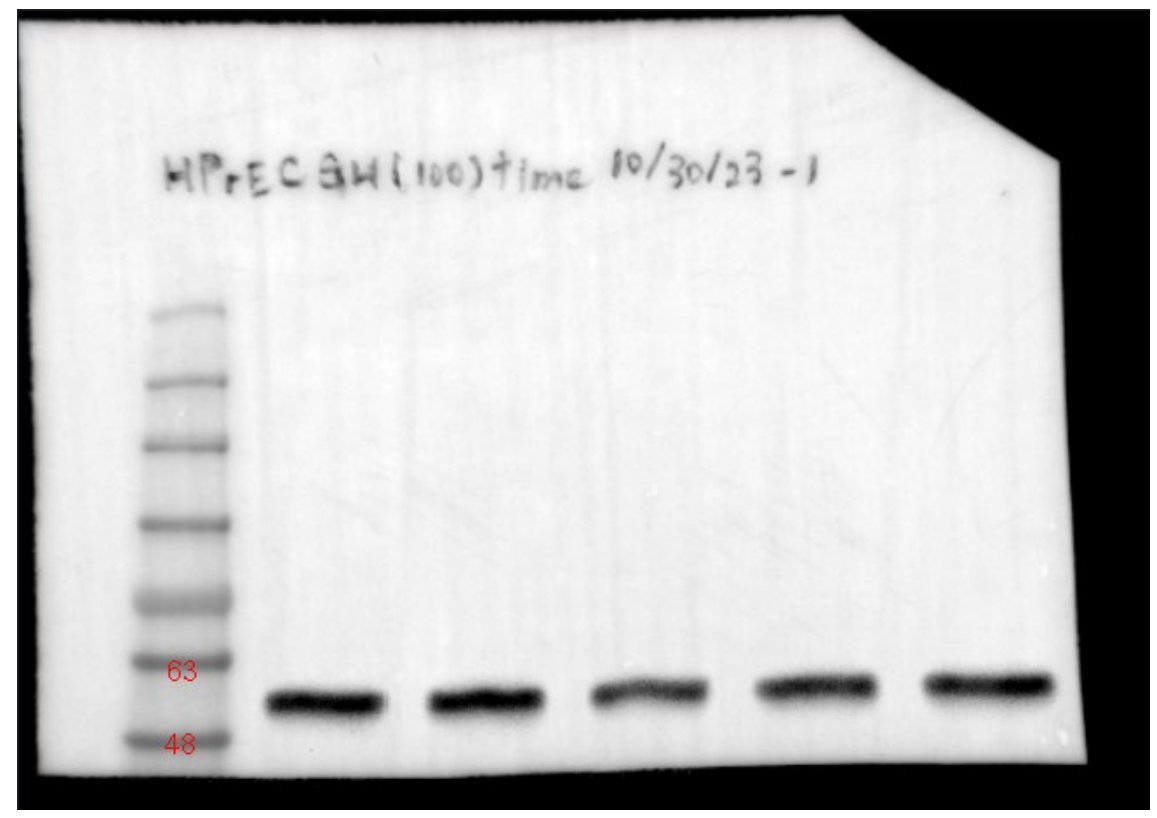


p53

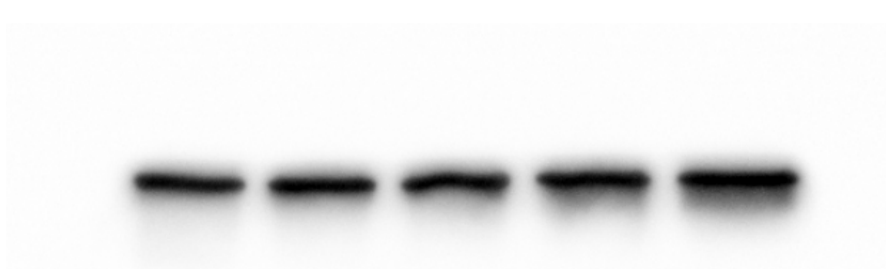

GAPDH-1

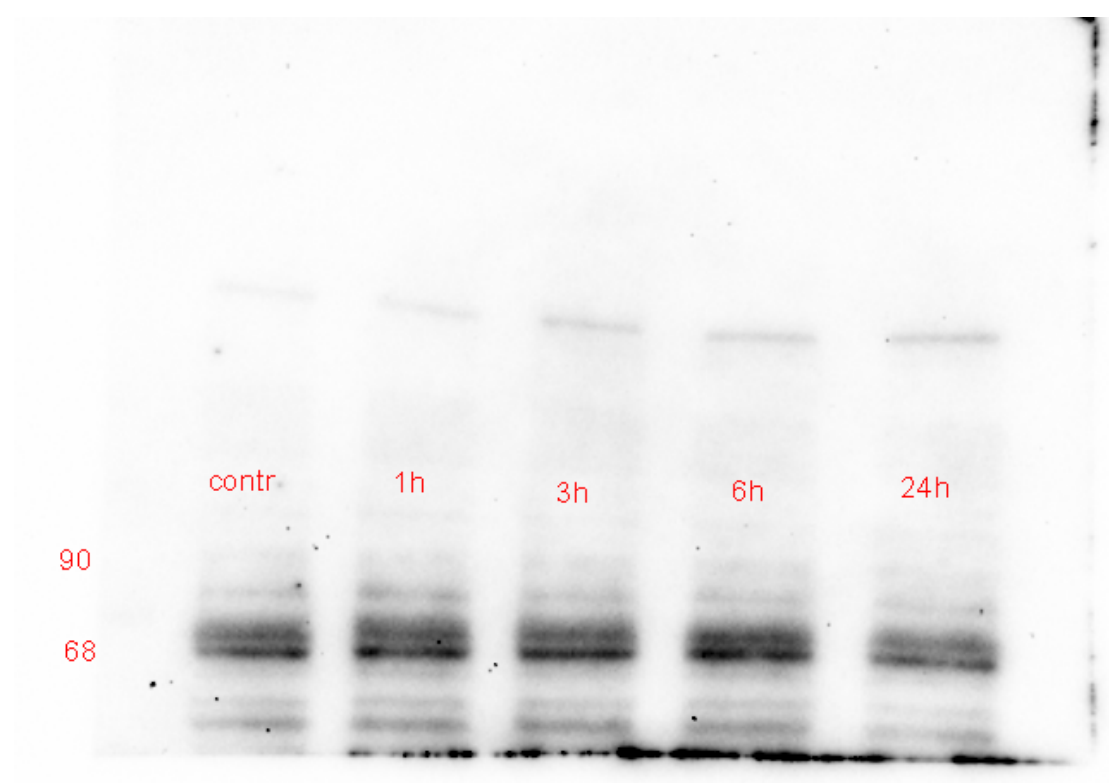


Wip1

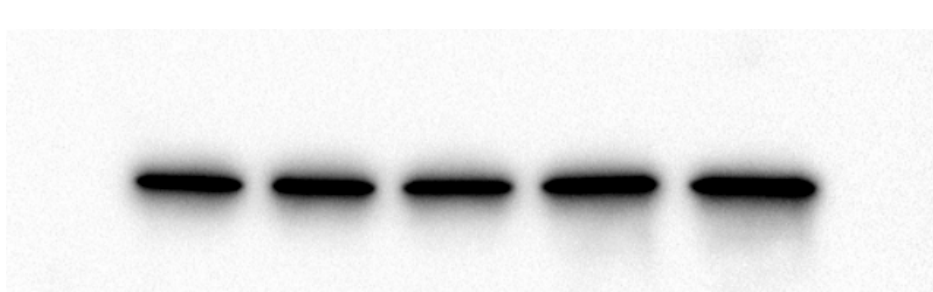

GAPDH-2

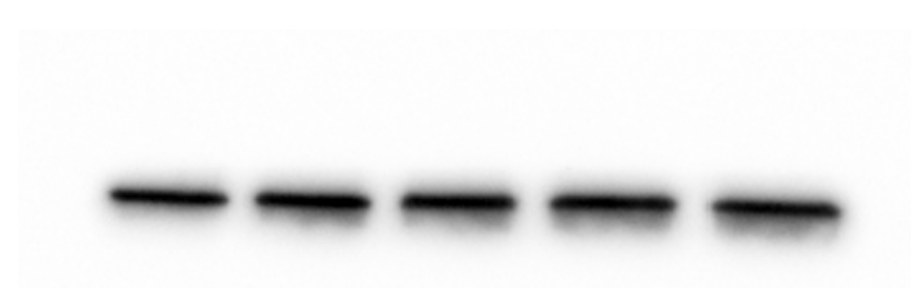

GAPDH-3

# Full unedited gels for Supplemental Figure 5A, 5C, 5E

## Supplemental Figure 5A

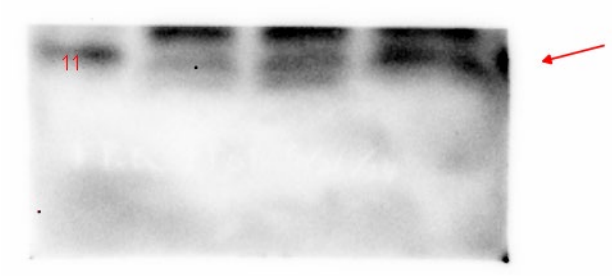

IGF-1

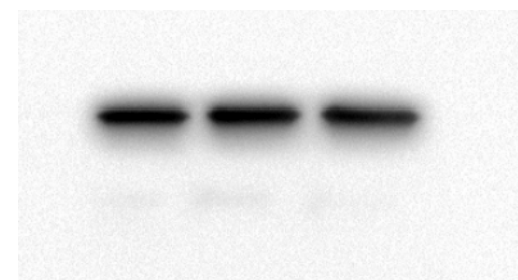

GAPDH

---

## Supplemental Figure 5C

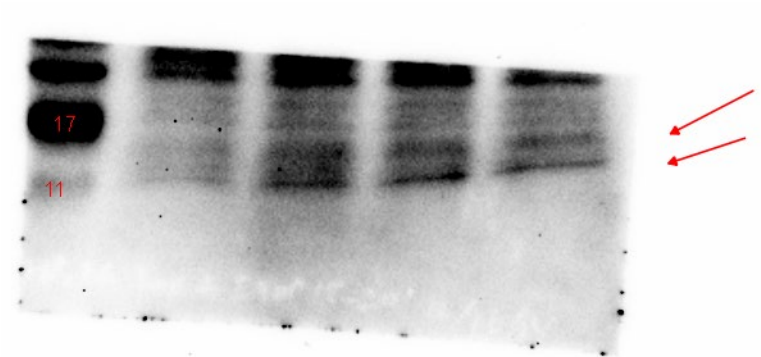

IGF-1

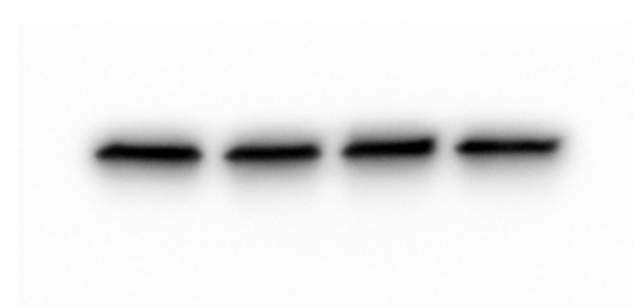

GAPDH

---

## Supplemental Figure 5E

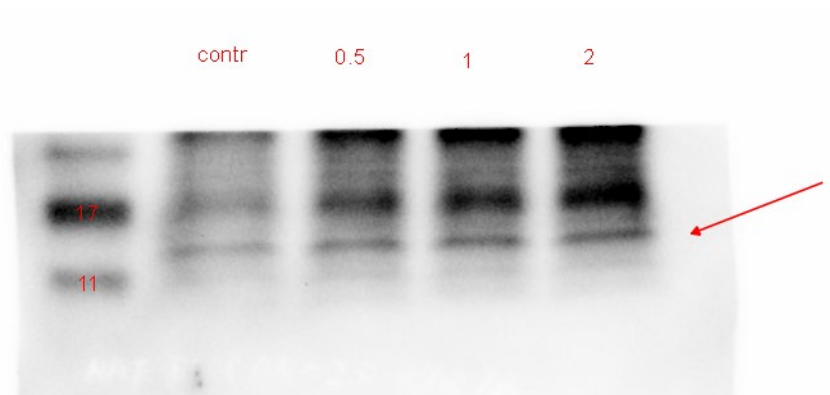


IGF-1

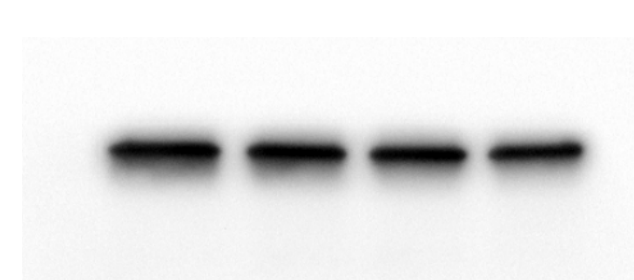

GAPDH

# Full unedited gels for Supplemental Figure 5G, 5I, 5K

## Supplemental Figure 5G

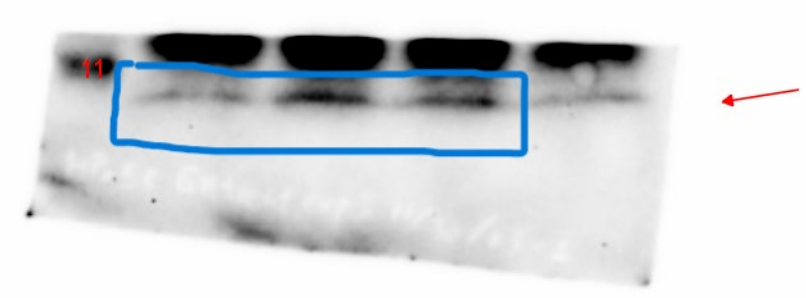

IGF-1

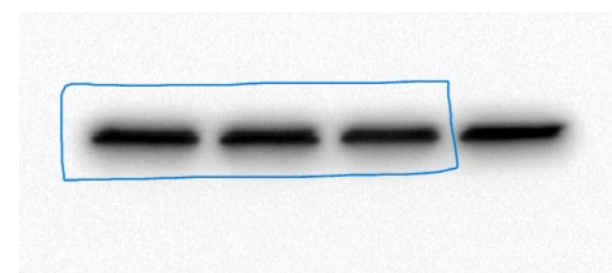

GAPDH

---

## Supplemental Figure 5I

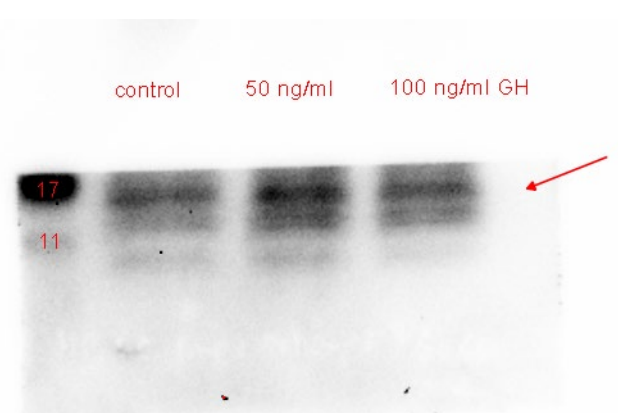


IGF-1

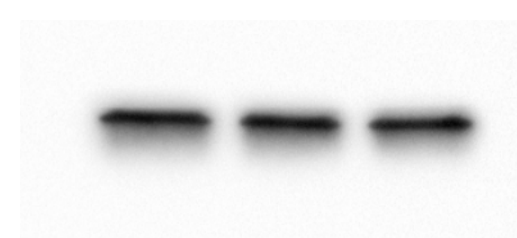

GAPDH

---

## Supplemental Figure 5K

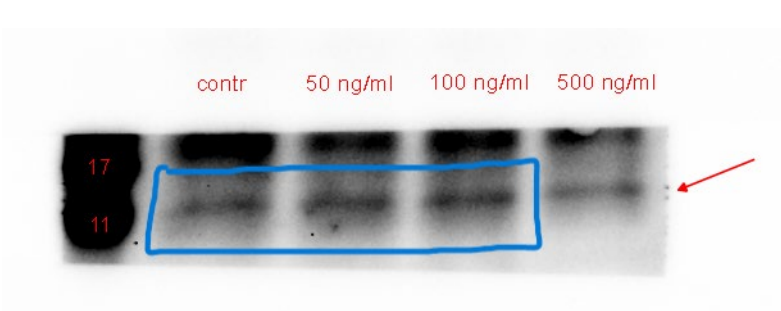


IGF-1

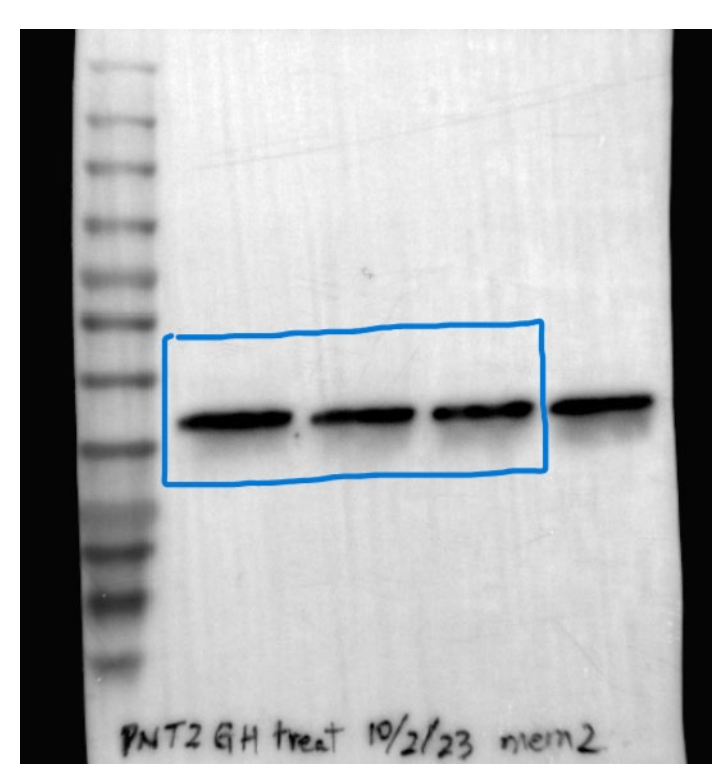


GAPDH

# Full unedited gels for Supplemental Figure 5M, 5O, 5Q

## Supplemental Figure 5M

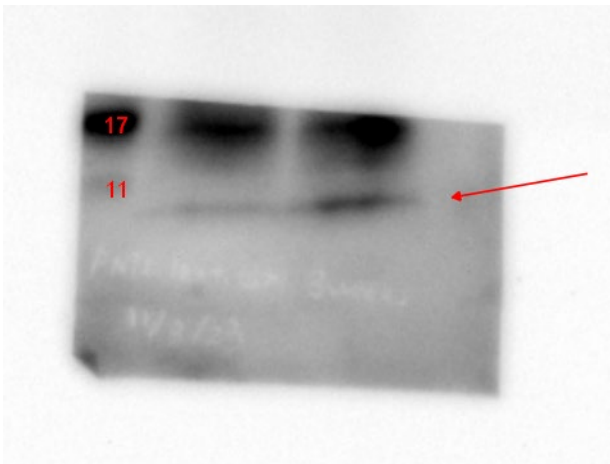


IGF-1

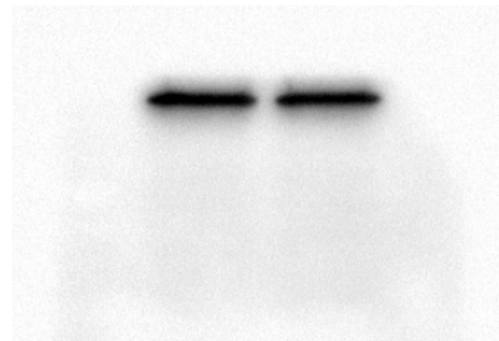

GAPDH

## Supplemental Figure 5O

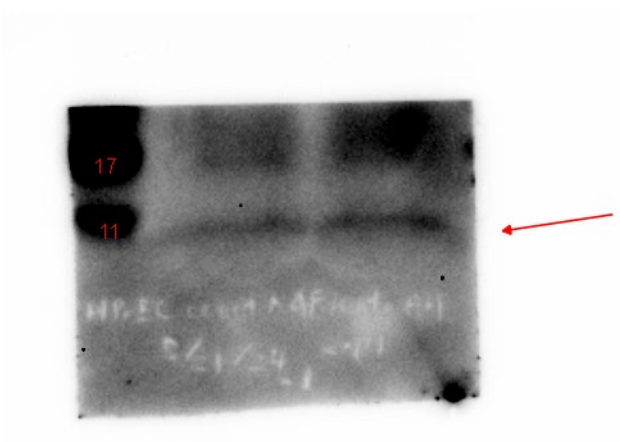


IGF-1

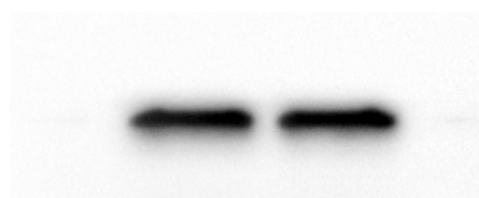

GAPDH

## Supplemental Figure 5Q

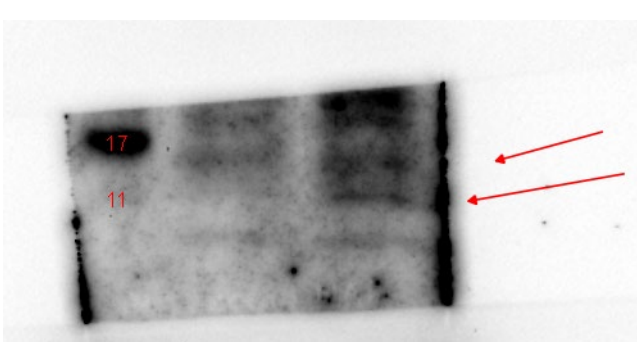


IGF-1

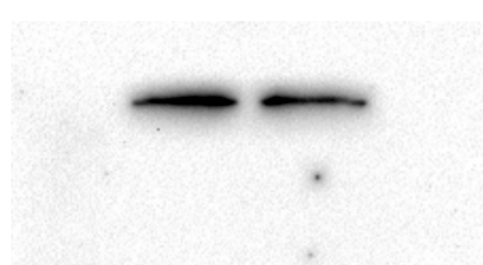

GAPDH
